# Warsaw set of emotional facial expression pictures: a validation study of facial display photographs

**DOI:** 10.3389/fpsyg.2014.01516

**Published:** 2015-01-05

**Authors:** Michal Olszanowski, Grzegorz Pochwatko, Krzysztof Kuklinski, Michal Scibor-Rylski, Peter Lewinski, Rafal K. Ohme

**Affiliations:** ^1^Department of Psychology, University of Social Sciences and HumanitiesWarsaw, Poland; ^2^Institute of Psychology, Polish Academy of SciencesWarsaw, Poland; ^3^Department of Communication, University of AmsterdamAmsterdam, Netherlands; ^4^Faculty in Wroclaw, University of Social Sciences and HumanitiesWroclaw, Poland

**Keywords:** facial expressions of emotion, facial expression recognition, basic emotions, emotion, validation, pictures, face perception, emotion recognition

## Abstract

Emotional facial expressions play a critical role in theories of emotion and figure prominently in research on almost every aspect of emotion. This article provides a background for a new database of basic emotional expressions. The goal in creating this set was to provide high quality photographs of genuine facial expressions. Thus, after proper training, participants were inclined to express “felt” emotions. The novel approach taken in this study was also used to establish whether a given expression was perceived as intended by untrained judges. The judgment task for perceivers was designed to be sensitive to subtle changes in meaning caused by the way an emotional display was evoked and expressed. Consequently, this allowed us to measure the purity and intensity of emotional displays, which are parameters that validation methods used by other researchers do not capture. The final set is comprised of those pictures that received the highest recognition marks (e.g., accuracy with intended display) from independent judges, totaling 210 high quality photographs of 30 individuals. Descriptions of the accuracy, intensity, and purity of displayed emotion as well as FACS AU's codes are provided for each picture. Given the unique methodology applied to gathering and validating this set of pictures, it may be a useful tool for research using face stimuli. The Warsaw Set of Emotional Facial Expression Pictures (WSEFEP) is freely accessible to the scientific community for non-commercial use by request at http://www.emotional-face.org.

## Introduction

The human face is considered to be a unique social stimulus that provides crucial information about another person. Information such as identity, gender, age, ethnicity, social origins, and physical attractiveness is easily accessible from the face, and plays a fundamental role in forming impressions about others. Moreover, a special place in facial communication is reserved for expression of emotions. Basic properties of facial expressions were described over a century ago by Darwin (1872/1988) and have since been the subject of interest for many researchers (see Ekman and Rosenberg, 2005). A review of recently published papers indicates that facial expressions figure prominently in research on almost every aspect of emotion, including psychophysiology (Dimberg et al., 2011), neural bases (Mattavelli et al., 2014), development (Mancini et al., 2013; Parker et al., 2013), perception (Barrett and Kensinger, 2010), social processes (Hareli et al., 2009; Schneider et al., 2013), emotion disorders (Bourke et al., 2010), and even human-computer interaction (Kharat and Dudul, 2009). Facial expressions are central to several leading theories of emotion (Tomkins, 1962; Ekman, 1992; Izard, 1993) and continue to be debated in emotion science, as their meaning, rules of display, and interpretation are still in question (Ekman, 1993, 1997; Fridlund, 1994; Russell, 1994; Wierzbicka, 1995). Even so, the observed cross-cultural easiness and unambiguity of so-called basic emotion expression, as well as the similarity of evoked reactions, makes it a crucial affective stimulus in multiple research purposes (see Ekman and Rosenberg, 2005). Therefore, access to standardized sets of pictures containing facial expressions of emotions is an important factor for improving research quality. This article reports the background information for a new database of basic emotional expressions: the Warsaw Set of Emotional Facial Expression Pictures (WSEFEP).

## Comparing sets of emotional expressions

One of the first and most frequently used sets is the Pictures of Facial Affect (POFA) set made by Ekman and Friesen (1976), alongside the Japanese and Caucasian Facial Expressions of Emotion (JACFEE) set and the Japanese and Caucasian Neutral Faces (JACNeuF) set created by Matsumoto and Ekman (1989; Biehl et al., 1997). POFA contains 110 black and white pictures of 16 models (Caucasians), and JACFEE includes pictures of 28 models: 14 Caucasians and 14 Japanese, with even gender split. Every model was photographed neutrally and shows one of the seven basic emotions: happiness, anger, fear, surprise, sadness, disgust, and contempt. Using the Facial Action Coding System (FACS; Ekman et al., 2002), a tool they independently developed, they were able to produce pictures of standardized facial expressions that were intended to represent “prototypical displays” of emotions.

Black and white photographs were also gathered for the Montreal Set of Facial Displays of Emotion (MSFDE) by Beaupre and Hess (2005). This set includes pictures of 32 models: Chinese, French-Canadians, and sub-Saharan Africans—eight for each group, four male and four female. Each individual was photographed while expressing five of the basic emotions (happiness, anger, fear, sadness, and disgust) and a neutral expression. Surprise was not included, and the pictures were standardized using FACS. One of the most elaborate sets is the Karolinska Directed Emotional Faces (KDEF; Goeleven et al., 2008) set, which consists of 4900 color pictures of 70 individuals. This set contains pictures of the six basic emotions and includes directness of the display with pictures taken from five different angles. The material was developed in 1998 by Daniel Lundqvist, Anders Flykt, and Arne Öhman at Karolinska Institute. Another example is the NimStim Face Stimulus Set, which was developed and first published in 2002 by The Research Network on Early Experience and Brain Development (Tottenham et al., 2009) and contains 646 pictures of 42 models: 25 Caucasian-American, two Latin-American, 10 African-American, and five Asian-American. Actors were instructed to pose eight expressions: happy, sad, angry, fearful, surprised, disgusted, neutral, and calm. For each expression, separate open- and closed-mouth versions were posed, except for surprise, which was only posed with an open mouth. The set was standardized according to the ratings of independent judges.

Also worth mentioning are several more recently developed sets. Tracy et al. (2009; UCDSEE) published a set of emotional expressions, but aside from the seven basic emotions (including contempt) they also added three “self-conscious emotions”: embarrassment, pride, and shame. The set features only two European American and two West-African models (four in total). Next is the Radboud Facial Database (RaFD; Langner et al., 2010), which features 49 child and adult models showing seven basic emotions. The RaFD includes averted and direct gaze orientations, and photographs taken from five different angles. Amsterdam Dynamic Facial Expression Set (ADFES, Van Der Schalk et al., 2011) includes a face-forward version and two different head-turning versions (faces turning toward and away from viewers), North-European and Mediterranean models (male and female, 22 in total), and nine discrete emotions (joy, anger, fear, sadness, surprise, disgust, contempt, pride, and embarrassment) filmed on a video camera. Kaulard et al. (2012) introduced the MPI Facial Expression Database that also contains conversational expressions in addition to emotions. The database contains 55 different facial expressions performed by 19 German participants. Finally, Ebner et al. (2010) published FACES—a quite unique dataset containing 171 faces of young as well as middle-aged and older Caucasian women and men—all posing with six facial expressions: neutrality, sadness, disgust, fear, anger, and happiness.

In most of the sets described above (e.g., JACFEE, MSFDE, ADFES, RaFD, UCDSEE, and FACES) facial displays were posed, which mean participants were first taught how to activate particular muscular units and show prototypical expressions (e.g., according to FACS descriptions) prior to photography sessions. However, current studies show the sensitivity of participants to genuine and posed emotional displays (McLellan et al., 2010, 2012). Moreover, we can observe the diversity of ways individuals typically express their emotions (Cohn et al., 2002). This may cause the expression of anger to include (but not necessarily lowering brows, while upper lids might be alternatively tightened or pulled up, upper and lower lips might be tightened together, or upper lips pulled up.

To overcome the mentioned limitations in the case of our own set, the WSEFEP, the main idea was to elicit emotional states and then photograph them. Thus, the stimuli would include the characteristics of genuine facial expressions that closely resemble the natural way of emotion expression by individuals, and therefore allow for the highest possible level of ecological validity. As a result, participants were inclined not to pose but instead express felt emotions, which were elicited during photo sessions. This was similar to the procedures of development for KDEF or MPI, where all participants received written instructions in advance. Our instructions entailed not only a description of the six different emotions and expressions that were acted during the photo session, but were also followed by guidance based on actor training methods (Stanislavski, 1936/1988). Additionally, visible parameters of facial muscle activity were not used until selection of the obtained material began.

Another property differentiating WSEFEP from other sets of emotional expressions is the process of selection and evaluation. The vast majority of the photographs from the described sets were first selected by their authors from a larger pool. Those photographs were then evaluated by independent judges and their evaluations were used to confirm proper categorization.

In our case, a different strategy was applied. The choices of competent judges constituted the initial selection, whilst the final set consisted of the photographs that received the highest marks from independent judges. We first selected several pictures representing a given emotion for each displayer and then asked judges to evaluate them. The final set consisted of photographs that received the highest marks and then additionally were coded by a certified FACS coder.

Moreover, we introduced a new method of validation in order to obtain a more precise description of the displayed emotions. The forced-choice method, which is criticized in the literature regarding basic emotion concepts (e.g., Russell, 1994; Nelson and Russell, 2013) and is also used in most of the picture validation studies, means that independent judges cannot choose precise equivalence of the displayed emotion. Thus, facial changes involving raised cheeks and pulled up lip corners could be classified as expressing joy just because it is the most proper label among the offered choices. However, independent judges may not find this label to be the most applicable. This could cause the subtle changes in meaning produced by the way an emotional display was evoked and expressed that were not captured by the previously used validation method.

The judgment task used in the present study was assumed to be sensitive to such differences and judges were not strictly limited to categories of basic emotion expression. They could also indicate if they considered the display to be a mix of emotions. Furthermore, this new method allowed us to describe displays in terms of their purity (accuracy with basic meaning) and intensity.

To summarize, the approach taken in this validation study was to establish whether a certain expression was perceived as intended by the displayer. We assumed that the independent judges' assessment of our stimuli would provide empirical support for the reliability and validity of this new set of facial expressions.

## Method for creating picture set of facial expressions of emotions

### Ethics statement

The expression database and the validation experiment described later in this manuscript used human volunteers. All actor-participants signed written consent, allowing for non-commercial use (i.e., scientific research only) and publication (i.e., non-commercial, scientific journals) of taken photos. All participants that took part in the validation experiment provided informed written consent prior to the experimental procedure. We analyzed only anonymous data. Participants and data from participants were treated according to the Declaration of Helsinki. The recording methods of the database and the subsequent validation experiment were approved by the local ethics committee of the first author's institution.

### Apparatus

A Canon EOS 30D camera with a prime portrait 85 mm lens was used during photography sessions. Sessions took place in an adapted room without daylight access. Lighting consisted of three halogen lamps with a total power of 3000 W (color temperature approximately 6000 K). The lamps were installed on stands in different points of the room and their light was additionally diffused in order to obtain natural face characteristics and increase visibility of subtle facial properties, e.g., wrinkles. The lighting and camera's properties allowed to record up to five frames per second—thus we were able to capture very rapid changes appearing on the face. The camera was placed approximately 2 m from the participant and about 4 m from the room's ending wall, which was covered with white woven fabric. The photographs were transformed using Adobe Photoshop Elements 8 software package.

### Preparing and elaborating photographed material

Concerning the specificity of elaboration, the procedure was conducted in a few stages. In the first stage, so-called “face banks” were used, and were gathered by an agency specializing in the recruitment of actors, extras, etc.. Native Polish applicants (*n* = 120) aged 20–30 were chosen and invited to individual meetings: 30-min photography sessions during which the expected work results were discussed. All of the participants provided signed consent to the recruitment agency and agreed to participate in the project before arriving to the laboratory. After the meeting they were informed that if they did not want to participate they are not obligated to continue the cooperation, and none of the taken photographs will be used in the future. The meetings also aimed to choose participants who had acting experience and positive motivation toward cooperation. After this stage, 60 people were selected and invited to take part in the project.

Natural emotional expression is involuntary, and bringing it under any control requires exercises allowing activation of particular muscles. Therefore, a set of exercises based on an actor training system, the Stanislavski method (1936/1988), was developed. The aim was to maximize the authenticity of expressed emotions in photography session conditions. The method assumes that realistic presentation of a given emotion should be based on the concept of emotional memory as a point of focus. Thus, actors must first recall an event when he or she felt given emotion, and then recall physical and psychological sensation. Additionally, this technique includes a method of “physical actions” in which emotions are produced through the use of actions. To do so, the actor performs a physical motion or a series of physical activities to create the desired emotional response for the character. For instance, feeling and expressing sadness presumes recalling sad personal experiences as well as some physical action, e.g., sighing and holding head in hands. The training consisted of three parts: (1) Theoretical presentation about the physiology of emotion and mimic expressions and demonstration of key elements essential for gaining a desired expression. During this stage, participants were also presented the theoretical foundations concerning the creation of the set, which facilitated understanding of the authors' intentions and communication during photography sessions. (2) Training workshops taking place under supervision of the set's authors, and (3) Training and exercises “at home.” The final stage was the photography session during which photographs of practiced expressions were registered. During the sessions, participants were first allowed to focus on the given emotion (so perform exercises evoking emotion) and then show facial expression of felt emotion to the camera. No beards, mustaches, earrings or eyeglasses, and preferably no visible make-up was allowed during this stage, yet matting foundation make-up was applied to avoid skin reflections.

After gathering the photographic material, primary evaluation was conducted. Photographs with appropriate activity of muscular/facial units were selected. Facial unit activity was specified according to descriptions of the FACS (Ekman et al., 2002) and characteristics of visible facial changes caused by contraction or relaxation of one or more muscles (so called Action Units) during emotional expression (see Table 1 for details). At this stage, approximately 1000 photographs of 46 people were selected by our competent judges. The photographs were subjected to digital processing for format, resolution, and tone standardization. Pictures were initially cropped and resized to 1725 × 1168 pixels and the color-tone was balanced for a white background.

**Table 1:** **Facial activity characteristics used for picture selection, based on FACS (Ekman et al., 2002)**.

**Expression**	**Facial activity**
Joy	Raised cheek and lip corner pulled up, inner brow raised, nasolabial deepened
	Optionally: jaw dropped, lip corner depressed
Anger	Brow lowered, lid tightened, upper lid raised, lip tightened
	Optionally: Upper lip raised, chin raised, lip pressed
Disgust	Nose wrinkled, upper lips raised
	Optionally: Lip corner depressed, lower lip depressed
Fear	Inner and outer brow raised, brow lowered, upper lid raised, lips parted and stretched, jaw dropped and mouth stretched
Surprise	Inner and outer brow raised, jaw dropped
Sadness	Inner brow raised, brow lowered, lip corner depressed

## Validation method

### Independent judges

The sample consisted of 1362 participants (1101 females and 261 males, age *M* = 26.6, *SD* = 11.6) who were our independent judges. They completed the inventory (validation) in answer to ads on Polish internet forums and invitations sent by e-mail (see below for the description of the inventory with items). Participants were not asked to indicate their ethnicity and nationality. However, using the Polish language limited the possible number of foreign participants.

### Validation procedure

**Figure 1:**
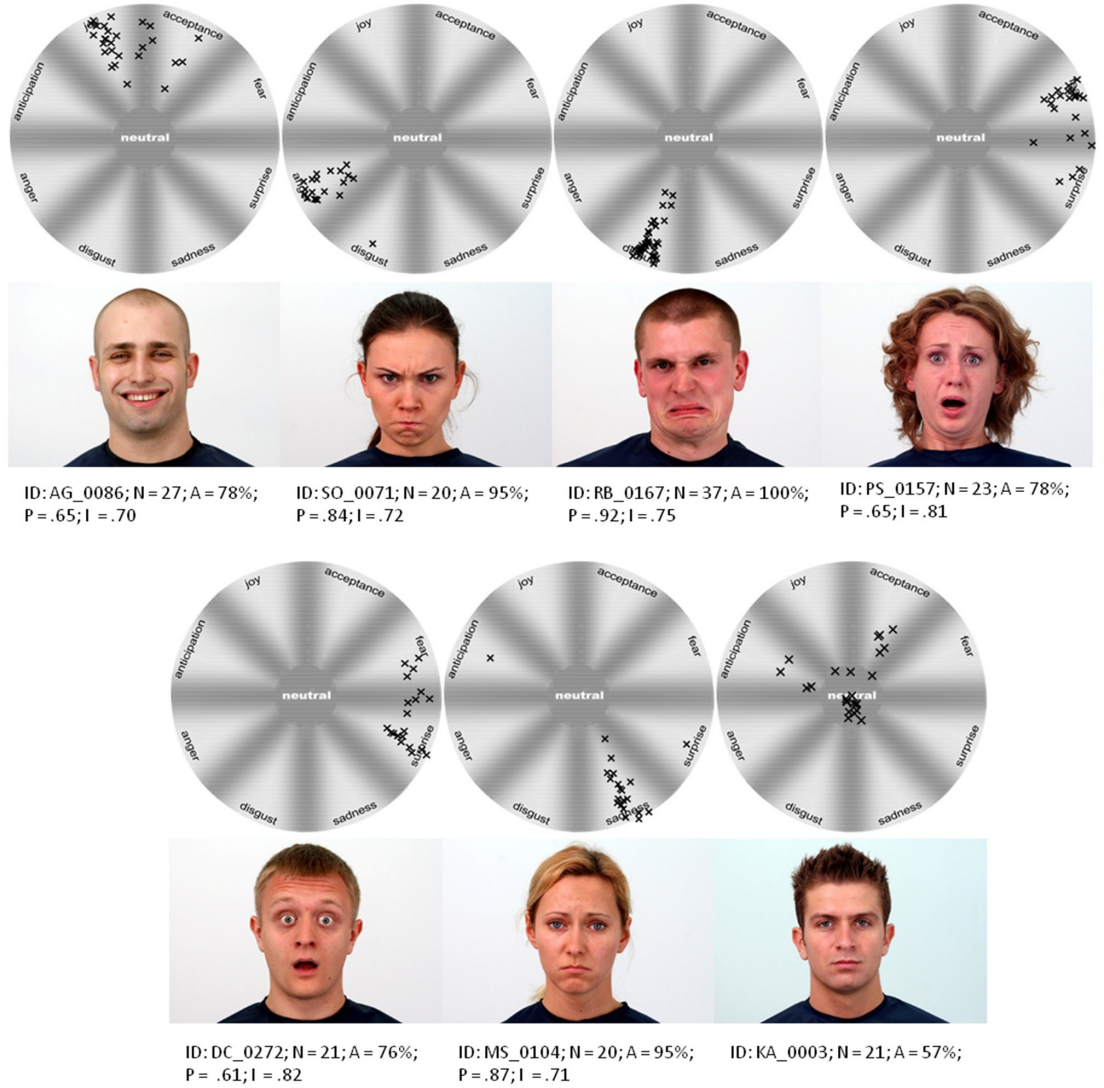
**Examples of photographs from WSEFEP with received ratings**. Each mark represents a rating from a single participant. The letters indicate: N, number participants evaluating picture; A, accuracy of displayed emotion; P, purity of displayed emotion, and I, intensity of displayed emotion.

We prepared an internet version of the inventory, in which a group of independent judges classified photographs into emotional categories and evaluated the intensity and purity of expression. Judges were asked to classify and evaluate individual photographs by pointing their mouse at a particular field in “the circle of emotions,” adapted based on Plutchik's circumplex model of emotions (1980)^1^. Applying this multidimensional form of classification allowed us to differentiate pictures according to clearness and intensity. The circle size was 500 × 500 pixels and the pixel map was created to precisely measure response values, and so the two-axis coordinates were saved as output data. In accordance with prepared instruction, the users could point at: (1) center of the field of a particular emotion category (which equally meant that in their opinion expression shown by the person in the photography is “clear”) or (2) field borders (which meant that the emotion is a mix of two neighboring emotions). Emotion intensity was measured at the same time: (1) showing the area closer to the circle meant low intensity of the expressed emotion or (2) closer to borders of the circle - high intensity of the expressed emotion. Emotional fields were all colored gradient gray, with darker areas at the borders between different emotions and brighter in the middle of the field. The area in the center of the circle meant neutral face expression. Additionally, the button named “other emotion” was located on the right site of the screen in case the participant would not be able to recognize the emotion. Fields outside of the wheel borders were deactivated, so only answers within the wheel or presses of the “other emotion” button were collected and allowed participants to continue to another trial. See Figure 1 for the visualization of our circle.

After receiving an instruction with examples, each participant evaluated 20 photographs randomly chosen from the database. A single photograph (resized to 800 × 542 pixels to fit the computer screen) was presented for 2 s and was followed by the circle of emotions, so that the examiner could select an answer by clicking on a chosen area of the circle. The participant had a maximum of 10 s to respond. If the time limit was reached, a message appeared indicating that the response time was exceeded and the next photograph was displayed. When evaluation of the photograph pool was finished the task could be continued, i.e., the participant assessed another 20 photographs or the task ended.

## Results/set validation

### Selecting photographs for the set

Independent judges' evaluations helped in further selection of photographs for the final set. The authors choose photographs of 40 individuals (16 females and 14 males) with neutral face and all six basic emotional expressions. The primary property of the chosen photographs was high conformity with the judges' marks (i.e., highest proportion of indications classifying expression as belonging to a particular category) and secondly the highest parameters of evaluating “purity” and “intensity.” Chosen photographs were FACS coded by certified specialist (see Appendix in Supplementary Material). Exemplary photographs with parameters of evaluations received from independent judges are presented in Figure 1.

**Figure 2:**
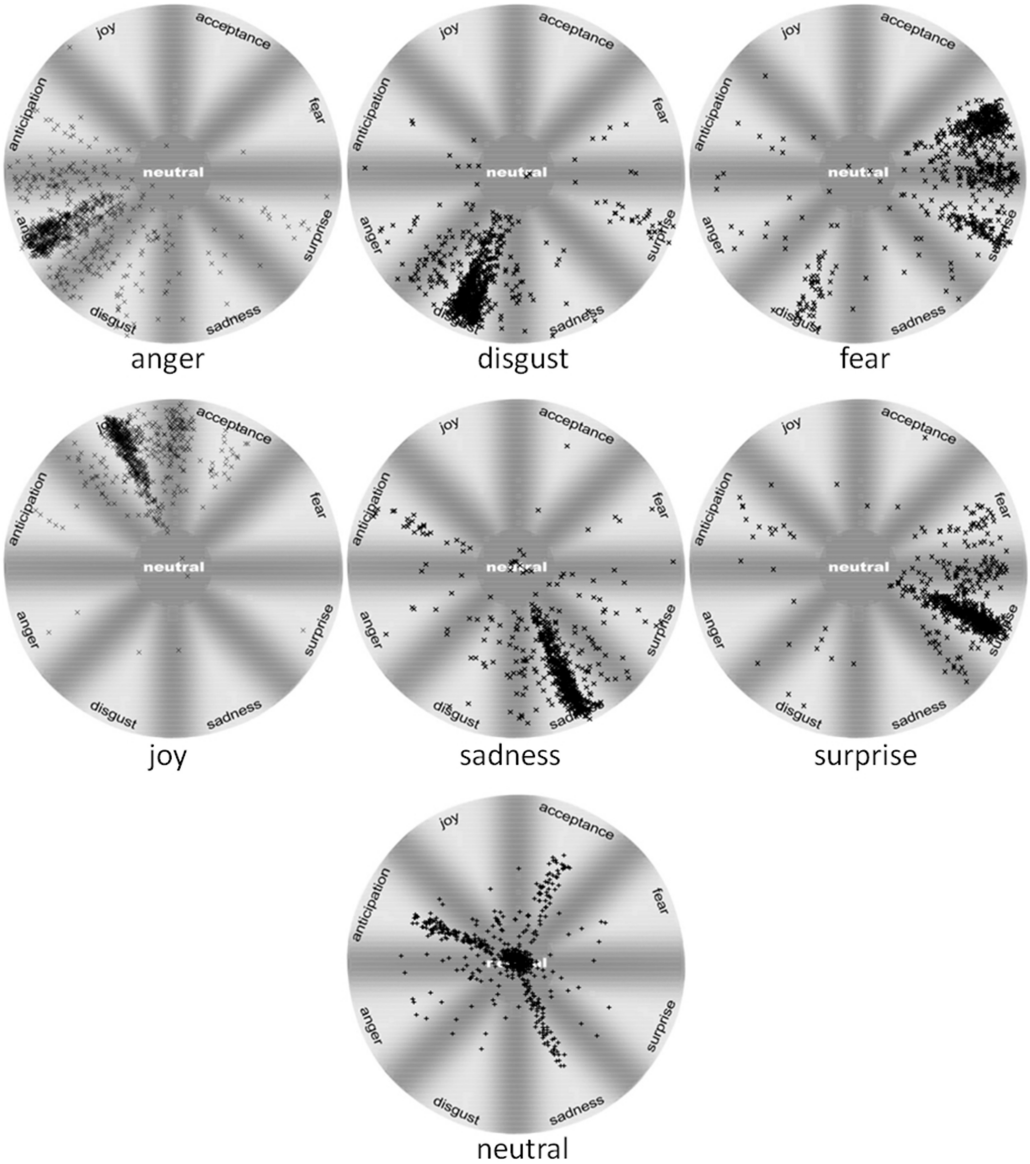
**Ratings distribution for facial expression of anger, disgust, fear, joy, sadness, surprise, and neutral faces from WSEFEP**.

Agreement ratings were first calculated for the chosen photographs and indicated how many independent judges chose the targeted emotion. The data showed the percentage of judges who pointed their answer within the field of intended display. The average agreement level for all photographs included in the set was 82.35% (*SD* = 13.68), with the highest agreement for disgust (*M* = 90.7%, *SD* = 6.19) and surprise (*M* = 89.02%, *SD* = 6.42). Other emotional displays received agreement of: joy *M* = 87.59%, *SD* = 8.38; anger *M* = *86.41*%, *SD* = 9.73; sadness *M* = 86.87%, *SD* = 11.69; fear *M* = 68.85%, *SD* = 13.14; and neutral *M* = 63.01%, *SD* = 13.35. One-Way ANOVA testing, carried out to compare agreement ratings, was significant *F*_(6, 203)_ = 28.49, *p* < 0.001. The *post-hoc* tests showed that agreement ratings for fear and neutral display were lower compared to all other emotions (in all cases: *p* > 0.001), while in the case of joy, anger, sadness, disgust, and surprise the level of recognition did not differ. See Figure 2 for details regarding answer distribution.

Distribution of answers within the intended display showed that the neutral display was frequently confused with anticipation (15%), sadness (8%), and acceptance (7%), while fear was confused with surprise (23%) and disgust (6%). Among other emotional displays that had high recognition accuracy, joy was confused with acceptance (11%), anger with disgust (8%) and anticipation (4%), sadness with anticipation, surprise, and disgust (each slightly above 3%), surprise with fear (6%), and finally disgust with surprise (3%). Categories, which were not mentioned, were confused rarely (less than 3% of total answers for a given display).

**Figure 3:**
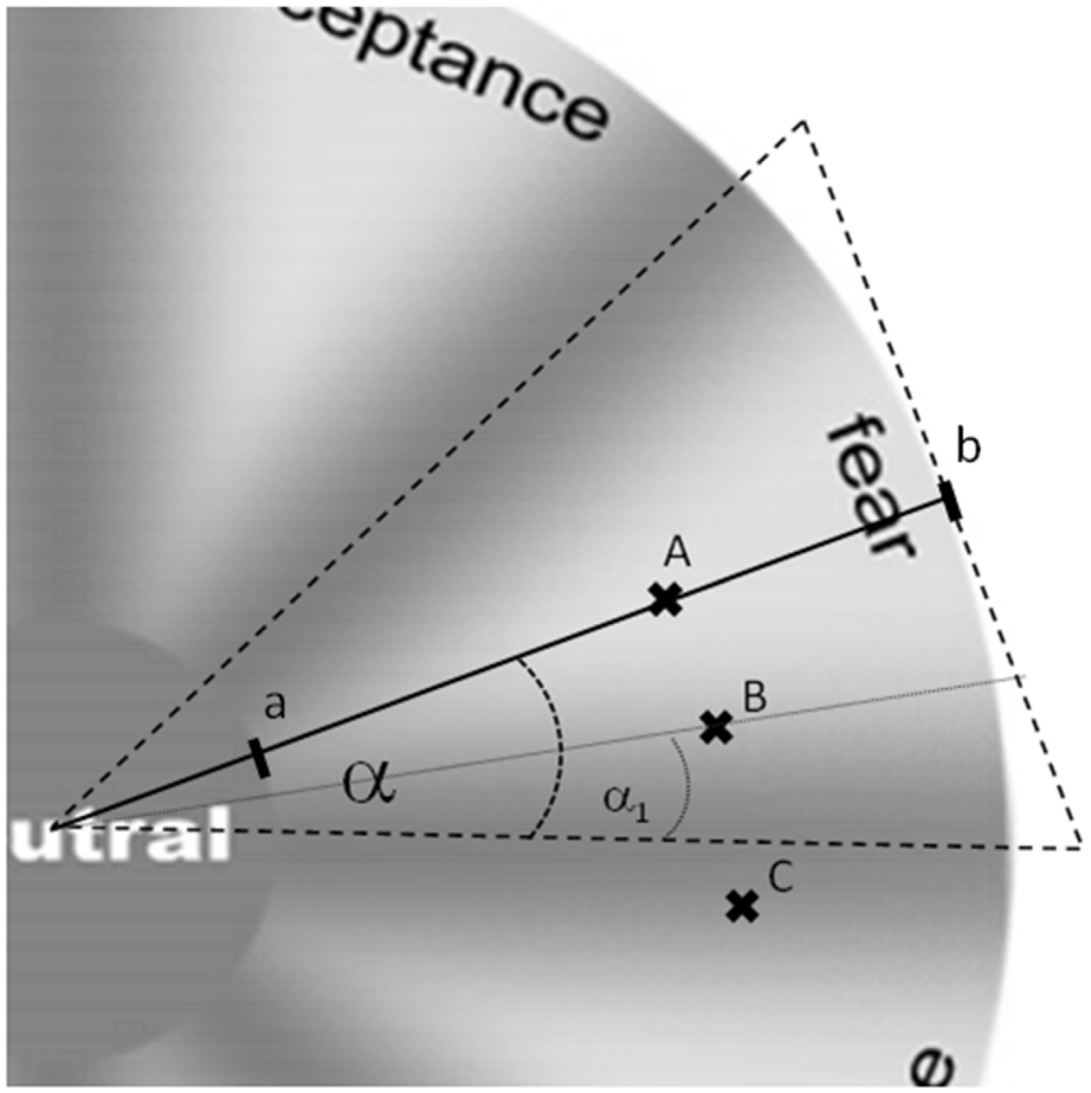
**Examples of purity and intensity scores**. Purity scores were based on relative difference of angles between the radius line directly crossing the middle of the field of a given emotion field (e.g., fear) and the radius line crossing through the answer point. In this example the answer “A” received 1 point as it is located directly on the line. Answer “B” received 5 point as α_1_ is 5 smaller than α. Answer “C” received 0 points as it is located outside of the emotion field (displayed emotion is not recognized as intended). Intensity scores were calculated as the relative distance of segment “ab” starting from point “a” (border of internal wheel labeled neutral scored 0) to the wheel border—point “b” (scored 1). Thus, answer “A” was scored 58 on the intensity scale.

The second parameter calculated from validation data was purity. This was an index showing whether the displayed emotion was considered clear or mixed with other emotions (the higher the rate, the purer the display). Score, ranging from 0 = extremely unclear expression to 1 = extremely clear, was calculated for each answer, based on angle (alpha) measured from the radius line crossing through the middle of the emotion field. One point was given if the answer was on the line and 0 points when the answer was given outside of the field of intended emotion. Score calculation for each answer was based on angle (alpha) measured from the middle line. Neutral displays were not measured for this parameter. See Figure 3 for detailed explanations.

Overall, the mean purity of all emotional displays included in the set was 0.72 (*SD* = 0.12). One-Way ANOVA comparing different emotions was significant *F*_(5, 174)_ = 18.07, *p* < 0.001, with only fear expressions (*M* = 0.58, *SD* = 0.12) being considered as less pure than all other emotional displays (*p* < 0.001): joy (*M* = 0.74, *SD* = 0.09), anger (*M* = 0.72, *SD* = 0.09), sadness (*M* = 0.77, *SD* = 0.11), disgust (*M* = 0.78, *SD* = 0.07), and surprise (*M* = 0.76, *SD* = 0.08). There were no significant differences between all other emotions.

Finally, we measured the intensity coefficient (0 = very low intensity to 1 = very high intensity) of the displayed emotion, based on distance from the middle circle. In this case, we assigned 1 point if the answer was given on the external border of the wheel and 0 points if the answer was given on the internal border (inside the wheel labeled as “neutral”). Mean intensity of displayed emotions were rated at 0.69 (*SD* = 0.08). One-Way ANOVA testing comparing emotional categories was significant *F*_(5, 174)_ = 10.15, *p* < 0.001. *Post-hoc* tests showed that fear displays (*M* = 0.75, *SD* = 0.05) were rated as more intensive than sadness (*M* = 0.64, *SD* = 0.08), *p* > 0.001, anger (*M* = 0.67, *SD* = 0.06), and joy (*M* = 0.68, *SD* = 0.08), *p* > 0.01. Additionally, sadness intensity (*M* = 0.64, *SD* = 0.08) was rated as lower than surprise (*M* = 0.73, *SD* = 0.05), *p* > 0.001, and disgust (*M* = 0.70, *SD* = 0.08), *p* > 0.01.

## General discussion

We have successfully developed an emotional facial expression database that represents six different emotional displays (joy, anger, sadness, fear, surprise, and disgust) presented by 30 subjects. The final set consists of the photographs that received the highest marks from independent judges. Importantly, the images are all of the same quality and represent genuine expressions that are as close to natural ones as possible.

Our analysis showed that the emotions displayed in the WSEFEP are very well recognized. Although, displayers were encouraged to spontaneous displays, the judges agreement rate found between intended and chosen expressions was high (82%). This parameter for WSEFEP is similar or higher to that reported in other sets (e.g., RaFD 82%, ADFES 76% and JACFEE 78% for displays of basic emotion) in which participants were taught to activate particular muscular units and show prototypical expressions. Moreover, muscular activation coded with FACS (Ekman et al., 2002) confirm that obtained facial displays are variants of basic emotions.

Additional ratings for purity of displayed emotions indicate that the displayed emotions were relatively close in distance to the expected prototypes. In addition, the intensity measures of the displayed emotions reflect the quality of the pictures, and allows for prediction of potential impact on participants exposed to these stimuli. The ratings, which we have collected on a number of critical variables, should be of interest to researchers using these faces and will allow for subsets of faces to be matched on these variables.

WSEFEP is a tool for research using face stimuli that provides a set of face images with detailed and comprehensive evaluations. The data presented in this article should raise experimenters' confidence about the validity and reliability of these expressions, as far as independent judges who are untrained in a face processing perceive the expressions. Clearly, there are other facial characteristics important for face processing, like attractiveness, symmetry of faces, masculinity/femininity, and other dimensions of affective characteristics like valence or arousal. Although, these were not systematically varied in the current database, future studies should gather data regarding these characteristics to further broaden the applicability of WSEFEP. Moreover, the photographic material gathered during sessions allows for development of further sub-sets containing expression at different intensities and with gaze directed away from camera.

To our knowledge this is the first time such a method of validation, as described in this paper, was used. This is one of the main contributors of WSEFEP to the field of facial expression and emotion as in comparison to other existing sets. Importantly, authors of any future sets may use our judgment task method and the circle of emotion to validate their own photographs of facial expressions of emotions. Moreover, future line of studies could possibly focus on comparing different techniques to obtain the best way of measuring facial expression recognition.

## Author contributions

Michal Olszanowski and Grzegorz Pochwatko—prepared and elaborate photographic material, designed the study and analyzed the data, wrote the manuscript, Krzysztof Kuklinski and Michał Ścibor-Rylski - prepared and elaborate photographic material, designed the study, Peter Lewinski—FACS-coded facial expression pictures and supported writing, Rafal K. Ohme—supervised the project.

### Conflict of interest statement

The authors declare that the research was conducted in the absence of any commercial or financial relationships that could be construed as a potential conflict of interest.

## Footnotes

^1^Plutchik (1980) suggested eight primary bipolar emotions: joy vs. sadness; anger vs. fear; trust vs. disgust; and surprise vs. anticipation. Additionally, his circumplex model makes connections between the idea of an emotion circle and a color wheel. Like colors, primary emotions can be expressed at different intensities and can mix with one another to form different emotions. Thus, for example, anger and disgust combine to contempt, while joy and trust result in love.

## Supplementary material

Data Sheet 1 **Validation data for complete picture dataset with Action Units (FACS) codes**.

Data Sheet 2 **WSEFEP - complete pictures dataset**.
