# Supplementary material for: Warsaw set of emotional facial expression pictures: a validation study of facial display photographs
Source: Front Psychol. 2015 Jan 5;5:1516. doi: 10.3389/fpsyg.2014.01516 (PMC4283518; doi:10.3389/fpsyg.2014.01516)
Supplement: Data Sheet 1 — Validation data for complete picture dataset with Action Units (FACS) codes. [file DataSheet1.DOCX]

| **Displayer ID** | **Picture ID** | **Male/ Female** | **Display** | **Agreement (%)** | **Putity (0-1)** | **Intensity (0-1)** | **N** | **FACS** |
| --- | --- | --- | --- | --- | --- | --- | --- | --- |
| AD | AD_7885.jpg | F | neutral | 53 | x | x | 17 | 7B |
| AD | AD_7950.jpg | F | joy | 92 | 0,77 | 0,72 | 36 | 1A+2A+6D+12D+25D+26B |
| AD | AD_8286.jpg | F | disgust | 80 | 0,66 | 0,73 | 30 | 4B+7B+9D+10C+17C+58D |
| AD | AD_8397.jpg | F | surprise | 87 | 0,71 | 0,77 | 31 | 1C+2C+5C+25D+26E+58B |
| AD | AD_8432.jpg | F | fear | 57 | 0,43 | 0,82 | 23 | 1D+2D+5D+25D+26E+58E |
| AD | AD_8595.jpg | F | sadness | 86 | 0,75 | 0,54 | 29 | 15C+17C+43A+54B |
| AD | AD_9681.jpg | F | anger | 97 | 0,81 | 0,73 | 29 | 5C+7D+17B+18C+24D+54B |
| AG | AG_0011.jpg | M | neutral | 65 | x | x | 17 | 7B |
| AG | AG_0086.jpg | M | joy | 78 | 0,65 | 0,7 | 27 | 6D+12D+25C+26A |
| AG | AG_0283.jpg | M | surprise | 84 | 0,67 | 0,71 | 19 | 1C+2C+4B+5C+25C+26D |
| AG | AG_0424.jpg | M | fear | 60 | 0,49 | 0,82 | 20 | 1D+2D+4C+5D+20D+25D+26B |
| AG | AG_0666.jpg | M | disgust | 96 | 0,86 | 0,74 | 27 | 4C+7B+9D+15A+17B+L25A |
| AG | AG_1314.jpg | M | anger | 86 | 0,73 | 0,74 | 35 | 4D+5D+17D+24C+38C+54A |
| AG | AG_1460.jpg | M | sadness | 100 | 0,92 | 0,71 | 16 | 1A+17B |
| DC | DC_0014.jpg | M | neutral | 71 | x | x | 24 | 0 |
| DC | DC_0139.jpg | M | joy | 85 | 0,69 | 0,65 | 34 | 6E+12E+25C+26A |
| DC | DC_0272.jpg | M | surprise | 76 | 0,61 | 0,82 | 21 | 1E+2E+5E+25C+26E |
| DC | DC_0616.jpg | M | disgust | 91 | 0,75 | 0,57 | 22 | 4B+9C+17B+25A |
| DC | DC_0952.jpg | M | fear | 72 | 0,58 | 0,72 | 21 | 1B+2B+5E+20C+25D+26E+38B+58C |
| DC | DC_1317.jpg | M | anger | 100 | 0,89 | 0,73 | 24 | 4C+5C+23C+24C |
| DC | DC_1399.jpg | M | sadness | 85 | 0,71 | 0,56 | 20 | 4A+5C+17D |
| HW | HW_0006.jpg | M | neutral | 40 | x | x | 20 | 7B |
| HW | HW_0068.jpg | M | joy | 93 | 0,8 | 0,69 | 29 | 6D+12D+25D |
| HW | HW_0452.jpg | M | disgust | 81 | 0,71 | 0,67 | 26 | 9D+17B |
| HW | HW_1612.jpg | M | anger | 79 | 0,68 | 0,68 | 29 | 4D+5B+17C+23C+24C+38B |
| HW | HW_2057.jpg | M | surprise | 94 | 0,84 | 0,78 | 32 | 1B+2B+5A+25C+26D |
| HW | HW_2219.jpg | M | fear | 87 | 0,75 | 0,83 | 15 | 1C+2C+4B+5C+17B+20B+25B+58B |
| HW | HW_2478.jpg | M | sadness | 93 | 0,86 | 0,7 | 27 | 1B+4B+11A+17A |
| JG | JG_0024.jpg | M | joy | 93 | 0,84 | 0,67 | 15 | 6C+7B+12D+25C |
| JG | JG_0134.jpg | M | disgust | 100 | 0,91 | 0,69 | 23 | 4D+6B+7D+10C+17A+25A |
| JG | JG_1226.jpg | M | neutral | 80 | x | x | 15 | 0 |
| JG | JG_1329.jpg | M | surprise | 86 | 0,66 | 0,75 | 14 | 1B+2B+5D+25C+26B |
| JG | JG_1465.jpg | M | fear | 63 | 0,52 | 0,72 | 16 | 1D+2D+5C+16B+25D+39C |
| JG | JG_1632.jpg | M | sadness | 90 | 0,73 | 0,56 | 10 | 1A+4A+15B+17B |
| JG | JG_2291.jpg | M | anger | 67 | 0,56 | 0,71 | 21 | 4A+5B+7B+10D+15D+17D+23E |
| JS | JS_0008.jpg | F | neutral | 84 | x | x | 19 | 0 |
| JS | JS_0281.jpg | F | joy | 88 | 0,67 | 0,49 | 25 | 6C+12D+25C |
| JS | JS_0491.jpg | F | surprise | 83 | 0,66 | 0,71 | 23 | R1C+5C+25C+26B |
| JS | JS_0744.jpg | F | disgust | 83 | 0,68 | 0,7 | 30 | 7B+9D+17C+25A |
| JS | JS_1601.jpg | F | fear | 74 | 0,65 | 0,83 | 23 | 1A+4B+5E+25D+26C |
| JS | JS_2296.jpg | F | anger | 96 | 0,77 | 0,66 | 23 | 4C+5B+11C+17E+24D+28 |
| JS | JS_2987.jpg | F | sadness | 90 | 0,86 | 0,44 | 20 | 15D+17C |
| KA | KA_0003.jpg | M | neutral | 57 | x | x | 21 | 7A+12A |
| KA | KA_0043.jpg | M | joy | 90 | 0,78 | 0,78 | 31 | 6E+12E+25D |
| KA | KA_0535.jpg | M | disgust | 90 | 0,74 | 0,72 | 21 | 6D+9E+17D+25B |
| KA | KA_0884.jpg | M | surprise | 96 | 0,81 | 0,7 | 28 | 1E+2E+5D+25C+26B |
| KA | KA_1134.jpg | M | fear | 74 | 0,64 | 0,75 | 34 | 1C+2D+5C+10C+20C+25D |
| KA | KA_1616.jpg | M | anger | 91 | 0,74 | 0,71 | 33 | 5B+10B+17C+24C |
| KA | KA_2396.jpg | M | sadness | 89 | 0,85 | 0,62 | 19 | 1A+4A+5A+15B+17D |
| KL | KL_0024.jpg | F | neutral | 65 | x | x | 26 | 0 |
| KL | KL_0092.jpg | F | joy | 83 | 0,71 | 0,62 | 30 | 6B+12D+25B |
| KL | KL_0324.jpg | F | surprise | 92 | 0,69 | 0,73 | 26 | 1B+2C+5D+25C+26B |
| KL | KL_0697.jpg | F | disgust | 84 | 0,75 | 0,6 | 25 | 4D+6B+7D+9D+23D |
| KL | KL_0900.jpg | F | fear | 63 | 0,52 | 0,72 | 19 | 4B+5D+17B+21+25C |
| KL | KL_1182.jpg | F | anger | 85 | 0,71 | 0,67 | 27 | 24E |
| KL | KL_1438.jpg | F | sadness | 95 | 0,83 | 0,69 | 19 | 1C+4B+15C+17B |
| KM | KM_0017.jpg | M | neutral | 59 | x | x | 17 | 7B+25B |
| KM | KM_0137.jpg | M | joy | 94 | 0,79 | 0,74 | 31 | 6D+12E+25E |
| KM | KM_0620.jpg | M | anger | 89 | 0,82 | 0,73 | 28 | 4E+6B+7B+17D+23D+24B |
| KM | KM_1295.jpg | M | sadness | 74 | 0,63 | 0,62 | 23 | 1B+15D+17C |
| KM | KM_1583.jpg | M | disgust | 89 | 0,78 | 0,66 | 36 | 1C+2C+6D+7B+9E+17D |
| KM | KM_1797.jpg | M | surprise | 100 | 0,89 | 0,77 | 28 | 1C+2E+5E+25D+26D |
| KM | KM_1980.jpg | M | fear | 75 | 0,63 | 0,77 | 24 | 1D+2E+5E+20C+25D+38C |
| KO | KO_0031.jpg | F | neutral | 78 | x | x | 27 | 0 |
| KO | KO_0251.jpg | F | anger | 79 | 0,65 | 0,63 | 28 | 1B+4C+7D+17D+23E |
| KO | KO_0277.jpg | F | joy | 84 | 0,77 | 0,77 | 38 | 6E+12D+25D |
| KO | KO_0484.jpg | F | disgust | 94 | 0,81 | 0,7 | 34 | 4D+6D+7E+9E+25D |
| KO | KO_0624.jpg | F | surprise | 89 | 0,77 | 0,68 | 35 | 5D+25B |
| KO | KO_0665.jpg | F | sadness | 82 | 0,77 | 0,57 | 17 | 7A+11B+15B+17C |
| KO | KO_1082.jpg | F | fear | 72 | 0,55 | 0,75 | 18 | 1C+4D+5E+20B+25C+38 |
| KP | KP_0051.jpg | F | joy | 97 | 0,79 | 0,72 | 29 | 6C+12D+25E |
| KP | KP_0082.jpg | F | neutral | 94 | x | x | 17 | 10A |
| KP | KP_0225.jpg | F | surprise | 80 | 0,73 | 0,74 | 20 | 1E+2D+5D+25D+26C |
| KP | KP_0351.jpg | F | disgust | 94 | 0,81 | 0,71 | 35 | 9E+17D |
| KP | KP_0760.jpg | F | anger | 100 | 0,83 | 0,71 | 28 | 4D+11B+17D+24D |
| KP | KP_0991.jpg | F | sadness | 48 | 0,4 | 0,66 | 21 | 1B+4B+7C+11B+15C |
| KP | KP_1148.jpg | F | fear | 84 | 0,72 | 0,84 | 19 | 1D+2C+4B+5E+20E+21+25D+38C |
| KS | KS_0252.jpg | F | surprise | 96 | 0,9 | 0,77 | 27 | 1D+2D+5D+25D+26C |
| KS | KS_0624.jpg | F | fear | 82 | 0,67 | 0,8 | 28 | 1E+4C+5E+11B+25E+26B |
| KS | KS_0993.jpg | F | disgust | 100 | 0,78 | 0,8 | 29 | 7C+9E+17C+21 |
| KS | KS_2052.jpg | F | sadness | 100 | 0,87 | 0,62 | 22 | 1B+7C+11C+15C |
| KS | KS_2161.jpg | F | neutral | 63 | x | x | 27 | 0 |
| KS | KS_3640.jpg | F | anger | 76 | 0,67 | 0,73 | 42 | 4B+15D+17D+23D |
| KS | KS_4222.jpg | F | joy | 72 | 0,55 | 0,53 | 25 | 6D+12D |
| MB | MB_0026.jpg | F | neutral | 68 | x | x | 25 | 0 |
| MB | MB_0048.jpg | F | joy | 100 | 0,8 | 0,77 | 31 | 6D+12E+25E |
| MB | MB_0400.jpg | F | surprise | 86 | 0,7 | 0,75 | 21 | 1D+2E+5D+25C+26B |
| MB | MB_1031.jpg | F | fear | 74 | 0,61 | 0,81 | 31 | 2B+5D+20D+25C |
| MB | MB_1210.jpg | F | anger | 85 | 0,71 | 0,66 | 13 | 4D+5C+17C+23E |
| MB | MB_2133.jpg | F | disgust | 93 | 0,85 | 0,83 | 30 | 4C+7B+9E+21+25D |
| MB | MB_2362.jpg | F | sadness | 100 | 0,88 | 0,7 | 21 | 1A+4A+15C+17B |
| MG | MG_0330.jpg | M | joy | 92 | 0,78 | 0,74 | 36 | 6D+7B+12D+25D |
| MG | MG_0345.jpg | M | neutral | 76 | x | x | 17 | 0 |
| MG | MG_0754.jpg | M | sadness | 95 | 0,84 | 0,78 | 19 | 1E+4D+7B+17D |
| MG | MG_0928.jpg | M | disgust | 97 | 0,86 | 0,65 | 33 | 7C+9E+25C |
| MG | MG_1069.jpg | M | surprise | 92 | 0,81 | 0,66 | 24 | 1D+2C+5D+25C+26C |
| MG | MG_1280.jpg | M | fear | 77 | 0,62 | 0,61 | 22 | 1C+4C+25C |
| MG | MG_1317.jpg | M | anger | 88 | 0,79 | 0,58 | 26 | 4D+L11C+15C+17D+24D |
| MJ | MJ_0066.jpg | F | joy | 78 | 0,68 | 0,65 | 23 | 6D+12E+25C |
| MJ | MJ_0152.jpg | F | anger | 97 | 0,82 | 0,66 | 31 | 4D+7C+11C+23D |
| MJ | MJ_0271.jpg | F | fear | 42 | 0,34 | 0,73 | 26 | 1D+2C+4C+5C+7B+11C+20B+25C |
| MJ | MJ_0332.jpg | F | surprise | 92 | 0,75 | 0,74 | 26 | 1B+R2C+5C+25D+26C |
| MJ | MJ_0346.jpg | F | neutral | 48 | x | x | 23 | 0 |
| MJ | MJ_0370.jpg | F | disgust | 92 | 0,75 | 0,78 | 36 | 4C+6C+9E+17D+19+25C |
| MJ | MJ_0484.jpg | F | sadness | 88 | 0,71 | 0,72 | 26 | 1D+4D+7C+15E+17B |
| MK | MK_0001.jpg | M | neutral | 58 | x | x | 24 | 12A |
| MK | MK_0040.jpg | M | joy | 76 | 0,58 | 0,61 | 29 | 12D+25B |
| MK | MK_0173.jpg | M | anger | 82 | 0,64 | 0,63 | 33 | 4D+7B+23D |
| MK | MK_0255.jpg | M | surprise | 95 | 0,86 | 0,8 | 20 | 1E+2E+5D+25D+26B |
| MK | MK_0306.jpg | M | sadness | 86 | 0,76 | 0,47 | 21 | 1D+4C+15C |
| MK | MK_0364.jpg | M | fear | 63 | 0,53 | 0,76 | 24 | 1E+2E+5E+20B+25C |
| MK | MK_0496.jpg | M | disgust | 84 | 0,71 | 0,69 | 32 | 4D+L7D+9E+17C |
| MK1 | MK1_0007.jpg | F | neutral | 63 | x | x | 27 | 0 |
| MK1 | MK1_0087.jpg | F | joy | 97 | 0,81 | 0,74 | 38 | 6E+7C+12E+16C+25E |
| MK1 | MK1_0411.jpg | F | disgust | 89 | 0,79 | 0,76 | 19 | 6C+7C+9E+17C |
| MK1 | MK1_0461.jpg | F | anger | 95 | 0,8 | 0,6 | 20 | 4E+7C+17C+24D |
| MK1 | MK1_0746.jpg | F | fear | 56 | 0,46 | 0,71 | 27 | 1C+R2D+5D+20C+25C |
| MK1 | MK1_1427.jpg | F | surprise | 81 | 0,76 | 0,74 | 32 | 1D+2E+5E+25D+26C |
| MK1 | MK1_1982.jpg | F | sadness | 100 | 0,9 | 0,72 | 23 | 1D+4C+15C |
| MR | MR_0013.jpg | F | neutral | 67 | x | x | 21 | L12A |
| MR | MR_0055.jpg | F | joy | 68 | 0,53 | 0,62 | 25 | 6B+12D+25B |
| MR | MR_0619.jpg | F | surprise | 86 | 0,76 | 0,71 | 25 | 1E+2E+25C |
| MR | MR_0959.jpg | F | fear | 79 | 0,65 | 0,76 | 29 | 1D+2E+4C+5C+11B+20C+25B |
| MR | MR_1669.jpg | F | disgust | 100 | 0,88 | 0,78 | 22 | 7D+9E+17E+25A |
| MR | MR_2450.jpg | F | anger | 93 | 0,66 | 0,64 | 15 | 7C+17D+23C |
| MR | MR_2767.jpg | F | sadness | 97 | 0,8 | 0,69 | 35 | 1D+4B+15C |
| MR1 | MR1_0006.jpg | F | neutral | 65 | x | x | 23 | 14A+25A |
| MR1 | MR1_0132.jpg | F | joy | 97 | 0,9 | 0,76 | 33 | 6C+12E+25E |
| MR1 | MR1_0821.jpg | F | sadness | 78 | 0,67 | 0,65 | 23 | 7B+15C+17C |
| MR1 | MR1_1199.jpg | F | disgust | 92 | 0,77 | 0,74 | 26 | 4C+7D+9E+25E |
| MR1 | MR1_1418.jpg | F | surprise | 95 | 0,75 | 0,73 | 20 | 1D+2E+5C+25B+26B |
| MR1 | MR1_1519.jpg | F | fear | 67 | 0,55 | 0,65 | 33 | 1D+2D+5C+20B+25C |
| MR1 | MR1_1802.jpg | F | anger | 100 | 0,8 | 0,65 | 28 | 4D+17D+24E+34 |
| MR2 | MR2_0014.jpg | M | neutral | 71 | x | x | 14 | B24B |
| MR2 | MR2_0063.jpg | M | joy | 79 | 0,64 | 0,52 | 34 | 6D+7D+13D+25C |
| MR2 | MR2_0580.jpg | M | surprise | 93 | 0,76 | 0,73 | 28 | 1E+2E+5D+25B+26B |
| MR2 | MR2_0896.jpg | M | anger | 76 | 0,55 | 0,52 | 21 | 4D+23D |
| MR2 | MR2_1290.jpg | M | disgust | 85 | 0,75 | 0,63 | 27 | 6D+L7E+9E+17D |
| MR2 | MR2_1829.jpg | M | fear | 90 | 0,74 | 0,78 | 29 | 1E+2D+4D+5D+20C+25C |
| MR2 | MR2_2086.jpg | M | sadness | 95 | 0,85 | 0,72 | 21 | 1E+4C+15C+17C |
| MS | MS_0004.jpg | F | neutral | 65 | x | x | 31 | 14A |
| MS | MS_0104.jpg | F | sadness | 95 | 0,87 | 0,71 | 20 | 1C+4C+L11B+15B+17A |
| MS | MS_0226.jpg | F | fear | 35 | 0,24 | 0,66 | 26 | 1D+4C+5C+20D+21+25C |
| MS | MS_0291.jpg | F | joy | 79 | 0,65 | 0,67 | 42 | 6D+7B+12D+25D |
| MS | MS_0431.jpg | F | surprise | 100 | 0,88 | 0,7 | 34 | 1B+2B+5D+25C+26C |
| MS | MS_0627.jpg | F | disgust | 80 | 0,67 | 0,61 | 30 | 7C+10D+17C+21+25A |
| MS | MS_0695.jpg | F | anger | 67 | 0,53 | 0,63 | 39 | 7C+10C+17B+23C |
| OG | OG_6108.jpg | F | neutral | 85 | x | x | 20 | 0 |
| OG | OG_6189.jpg | F | joy | 96 | 0,85 | 0,64 | 28 | 6D+12D+25E |
| OG | OG_6390.jpg | F | disgust | 97 | 0,83 | 0,55 | 34 | 4C+7C+9D+25C |
| OG | OG_6484.jpg | F | anger | 90 | 0,75 | 0,67 | 41 | 4E+5C+7C+23D+24D+34 |
| OG | OG_6566.jpg | F | sadness | 69 | 0,61 | 0,63 | 16 | 1D+4D+7C+11B+17C |
| OG | OG_7620.jpg | F | fear | 53 | 0,49 | 0,64 | 30 | 1E+2E+5E+11B+20D+21+25C |
| OG | OG_7702.jpg | F | surprise | 89 | 0,78 | 0,64 | 19 | 1D+2D+5C+25C+26C |
| PA | PA_0006.jpg | M | neutral | 80 | x | x | 25 | 5A |
| PA | PA_0112.jpg | M | joy | 90 | 0,76 | 0,77 | 31 | 6E+12D+25D |
| PA | PA_0394.jpg | M | fear | 76 | 0,61 | 0,77 | 17 | 1E+2E+5E+20E+21+25D+26C |
| PA | PA_0840.jpg | M | surprise | 81 | 0,66 | 0,75 | 26 | 1E+2E+5D+25C+26B |
| PA | PA_0967.jpg | M | anger | 83 | 0,66 | 0,64 | 40 | 4E+7C+11D+17C+24E |
| PA | PA_1348.jpg | M | sadness | 87 | 0,74 | 0,67 | 23 | 1D+4B+11C+15C+17C |
| PA | PA_1701.jpg | M | disgust | 90 | 0,83 | 0,78 | 31 | 6C+7D+9E+25C |
| PB | PB_0001.jpg | M | neutral | 50 | x | x | 26 | 0 |
| PB | PB_0144.jpg | M | sadness | 77 | 0,72 | 0,71 | 26 | 1C+7A+11C+15A |
| PB | PB_0269.jpg | M | fear | 57 | 0,53 | 0,81 | 21 | 1E+2E+5E+20D+21+25C |
| PB | PB_0314.jpg | M | anger | 86 | 0,71 | 0,78 | 29 | 7B+9D+17D+24D |
| PB | PB_0442.jpg | M | disgust | 94 | 0,75 | 0,76 | 33 | 7B+9D+17B+21+25A |
| PB | PB_0499.jpg | M | joy | 90 | 0,76 | 0,63 | 31 | 6D+12E+25D |
| PB | PB_1383.jpg | M | surprise | 83 | 0,69 | 0,71 | 23 | 1D+2D+5D+25C+26C |
| PO | PO_0015.jpg | M | neutral | 59 | x | x | 17 | 0 |
| PO | PO_0124.jpg | M | joy | 95 | 0,77 | 0,68 | 37 | 6E+12D+25C |
| PO | PO_0553.jpg | M | surprise | 86 | 0,76 | 0,76 | 29 | 1E+2E+5E+25D+26C |
| PO | PO_0673.jpg | M | disgust | 81 | 0,7 | 0,66 | 42 | 6C+7D+9E+17C |
| PO | PO_0843.jpg | M | sadness | 97 | 0,86 | 0,68 | 31 | 1C+4C+15+17C |
| PO | PO_0951.jpg | M | anger | 76 | 0,64 | 0,8 | 33 | 4D+7C+10C+15C+17D+24C |
| PO | PO_1030.jpg | M | fear | 52 | 0,41 | 0,69 | 25 | 1D+2B+5E+20B+25C |
| PS | PS_0100.jpg | F | anger | 93 | 0,77 | 0,6 | 40 | 4C+5D+17B+23D+38B |
| PS | PS_0157.jpg | F | fear | 78 | 0,65 | 0,81 | 23 | 1E+5D+21+25D+26D |
| PS | PS_0216.jpg | F | neutral | 52 | x | x | 21 | 0 |
| PS | PS_0236.jpg | F | joy | 88 | 0,71 | 0,72 | 41 | 6D+12D+25D |
| PS | PS_0282.jpg | F | surprise | 93 | 0,82 | 0,58 | 28 | 1C+5D+25C |
| PS | PS_0719.jpg | F | disgust | 91 | 0,8 | 0,72 | 34 | L2C+7D+10D+17C |
| PS | PS_0746.jpg | F | sadness | 94 | 0,84 | 0,71 | 34 | 1C+4B+11B+15C+17B |
| RA | RA_0057.jpg | M | neutral | 72 | x | x | 23 | 0 |
| RA | RA_0317.jpg | M | joy | 91 | 0,88 | 0,7 | 26 | 6E+12D+25D |
| RA | RA_1215.jpg | M | sadness | 67 | 0,69 | 0,53 | 21 | 15C |
| RA | RA_1786.jpg | M | anger | 83 | 0,63 | 0,67 | 21 | 4D+7B+17E+24E+38 |
| RA | RA_2267.jpg | M | disgust | 89 | 0,73 | 0,74 | 26 | 7D+9E+17D |
| RA | RA_2800.jpg | M | surprise | 83 | 0,77 | 0,78 | 27 | 1E+2E+5E+25C |
| RA | RA_3483.jpg | M | fear | 76 | 0,81 | 0,64 | 18 | 1D+2E+5E+16D+25D |
| RB | RB_0006.jpg | M | neutral | 50 | x | x | 20 | 7B |
| RB | RB_0167.jpg | M | disgust | 100 | 0,92 | 0,75 | 37 | 9D+17D+21+25B |
| RB | RB_0255.jpg | M | surprise | 85 | 0,7 | 0,74 | 27 | 1E+2E+5D+25D+26C |
| RB | RB_0329.jpg | M | joy | 88 | 0,78 | 0,72 | 33 | 6D+12D+25D |
| RB | RB_0392.jpg | M | fear | 70 | 0,57 | 0,77 | 23 | 5E+20E+25C |
| RB | RB_0458.jpg | M | sadness | 86 | 0,75 | 0,55 | 28 | 11B+15C |
| RB | RB_0586.jpg | M | anger | 68 | 0,61 | 0,75 | 19 | 4C+5B+10C+17C+23D+24C |
| SO | SO_0028.jpg | F | joy | 97 | 0,85 | 0,8 | 34 | 6D+12E+25E+26C |
| SO | SO_0052.jpg | F | disgust | 89 | 0,71 | 0,62 | 27 | 9+25B |
| SO | SO_0071.jpg | F | anger | 95 | 0,84 | 0,72 | 20 | 4E+17C+23C+24C |
| SO | SO_0223.jpg | F | surprise | 100 | 0,94 | 0,72 | 27 | 5D+25C |
| SO | SO_0893.jpg | F | sadness | 84 | 0,7 | 0,66 | 19 | 1C+4C+15C+17C |
| SO | SO_1515.jpg | F | fear | 75 | 0,62 | 0,76 | 28 | 1D+4C+5C+21+25D+26D |
| SO | SO_2188.jpg | F | neutral | 91 | x | x | 33 | 0 |
| SS | SS_0018.jpg | F | joy | 77 | 0,58 | 0,64 | 22 | 6B+12E+25D |
| SS | SS_0032.jpg | F | surprise | 88 | 0,83 | 0,86 | 24 | 1C+2C+5D+22D+25C |
| SS | SS_0084.jpg | F | disgust | 93 | 0,85 | 0,78 | 30 | 9D+17D |
| SS | SS_0151.jpg | F | neutral | 81 | x | x | 21 | 17A |
| SS | SS_0302.jpg | F | anger | 92 | 0,71 | 0,61 | 24 | 4D+7B+17D+23C+24C |
| SS | SS_0539.jpg | F | sadness | 79 | 0,63 | 0,65 | 24 | 1B+15C |
| SS | SS_1188.jpg | F | fear | 84 | 0,69 | 0,78 | 31 | 1D+4A+5D+11B+25D |
