# Supplementary figures and images for: Warsaw set of emotional facial expression pictures: a validation study of facial display photographs (part 2 of 2)
Source: Front Psychol. 2015 Jan 5;5:1516. doi: 10.3389/fpsyg.2014.01516 (PMC4283518; doi:10.3389/fpsyg.2014.01516)

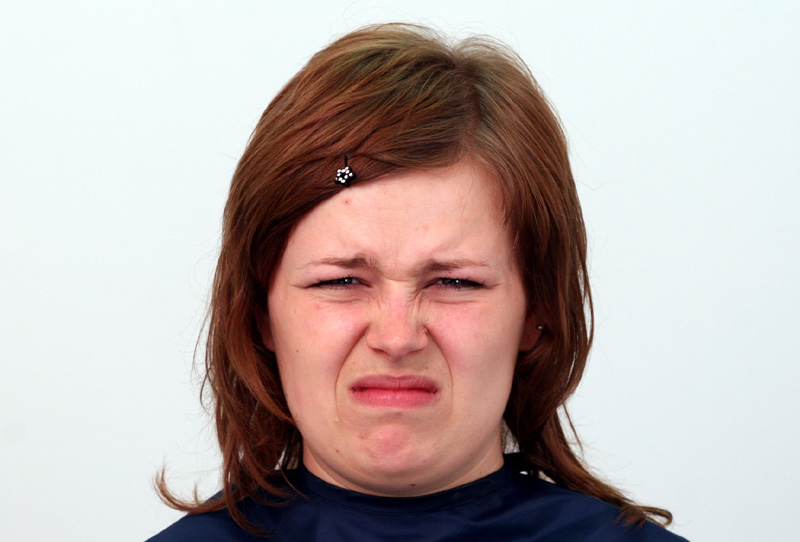

Supplement: Data Sheet 2 — WSEFEP - complete pictures dataset. [file DataSheet2.ZIP › MK1_0411-lo.jpg]

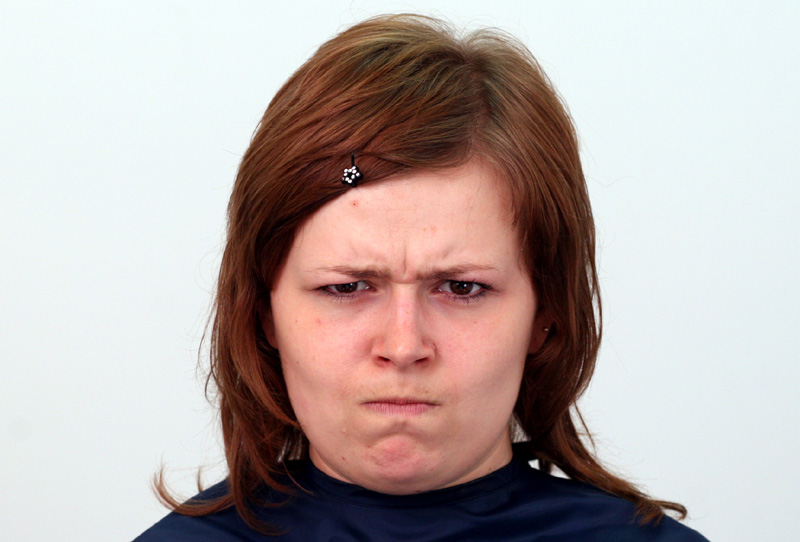

Supplement: Data Sheet 2 — WSEFEP - complete pictures dataset. [file DataSheet2.ZIP › MK1_0461-lo.jpg]

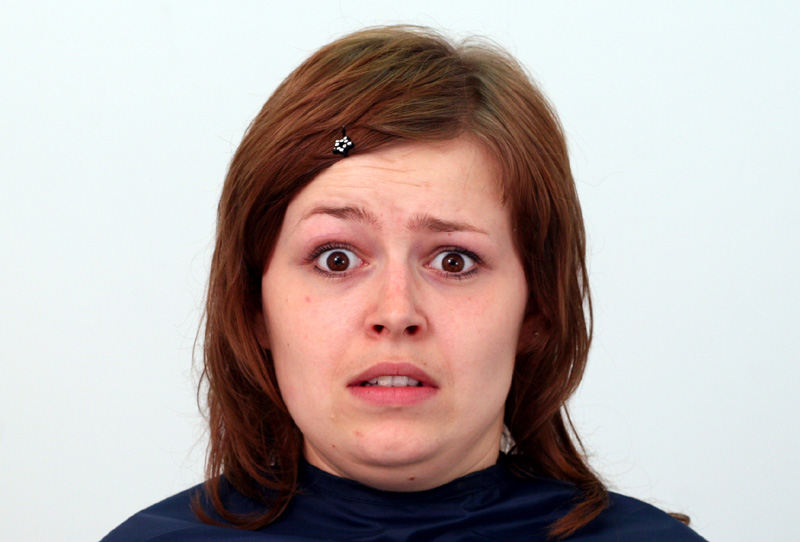

Supplement: Data Sheet 2 — WSEFEP - complete pictures dataset. [file DataSheet2.ZIP › MK1_0746-lo.jpg]

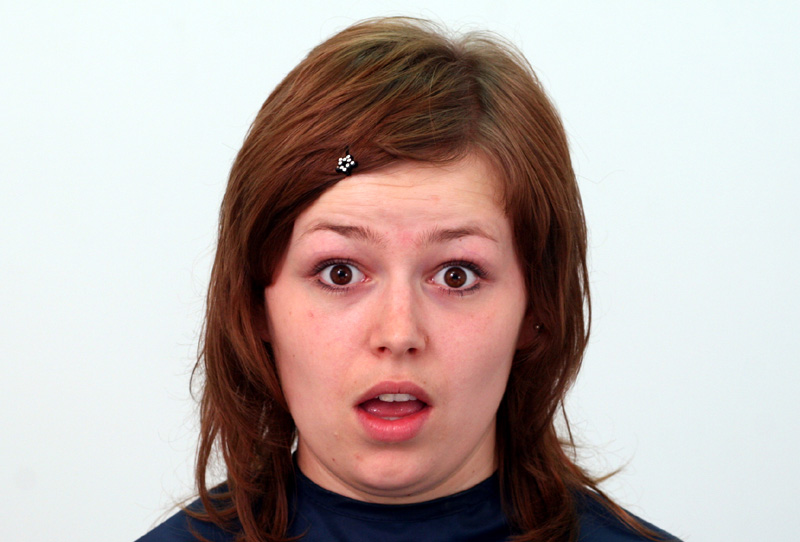

Supplement: Data Sheet 2 — WSEFEP - complete pictures dataset. [file DataSheet2.ZIP › MK1_1427-lo.jpg]

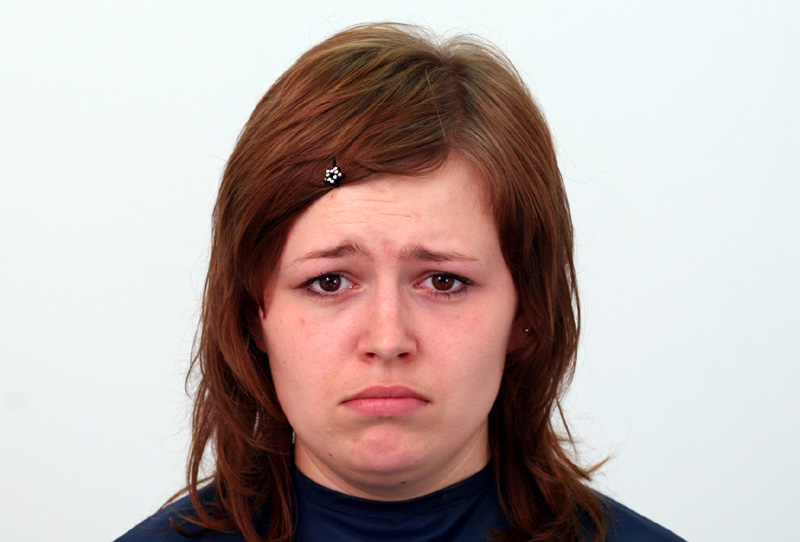

Supplement: Data Sheet 2 — WSEFEP - complete pictures dataset. [file DataSheet2.ZIP › MK1_1982-lo.jpg]

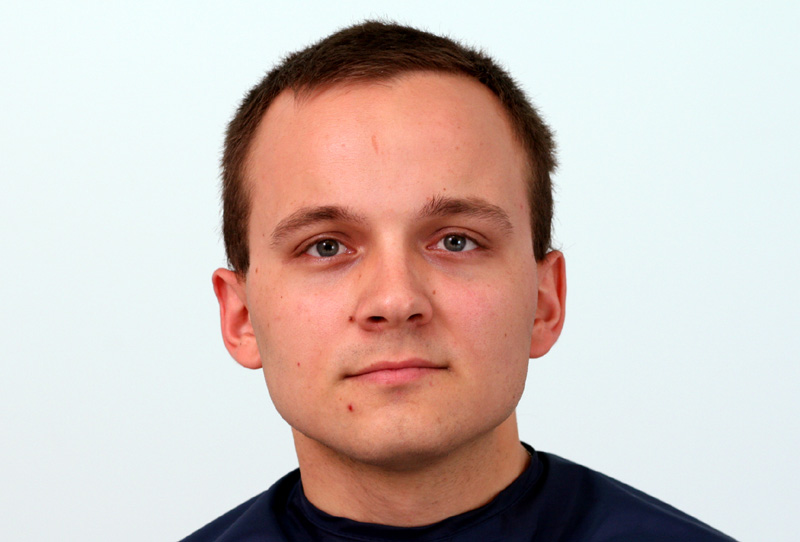

Supplement: Data Sheet 2 — WSEFEP - complete pictures dataset. [file DataSheet2.ZIP › MK_0001-lo.jpg]

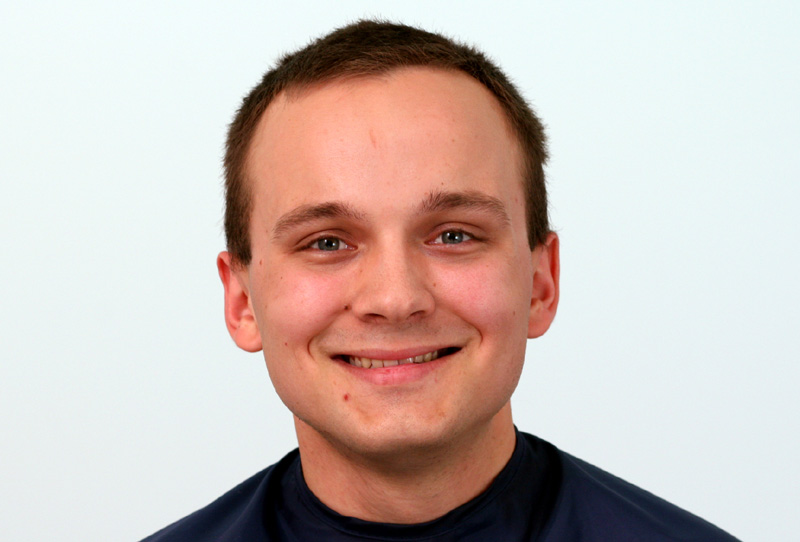

Supplement: Data Sheet 2 — WSEFEP - complete pictures dataset. [file DataSheet2.ZIP › MK_0040-lo.jpg]

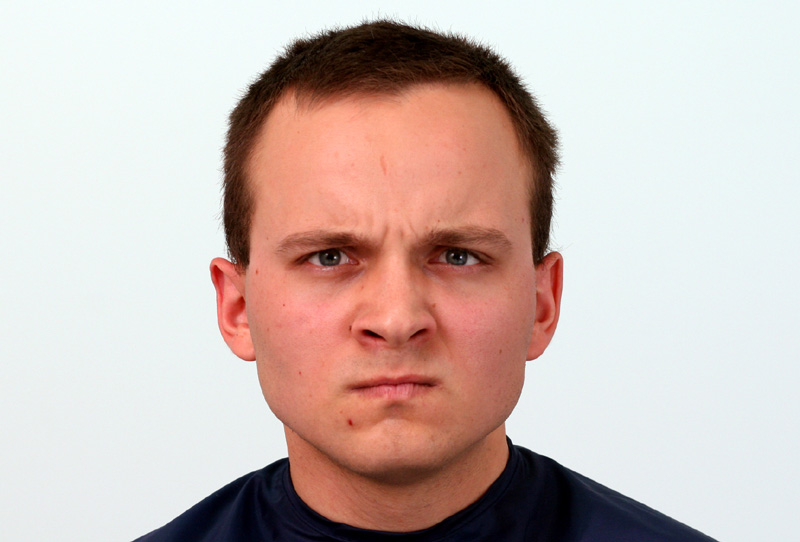

Supplement: Data Sheet 2 — WSEFEP - complete pictures dataset. [file DataSheet2.ZIP › MK_0173-lo.jpg]

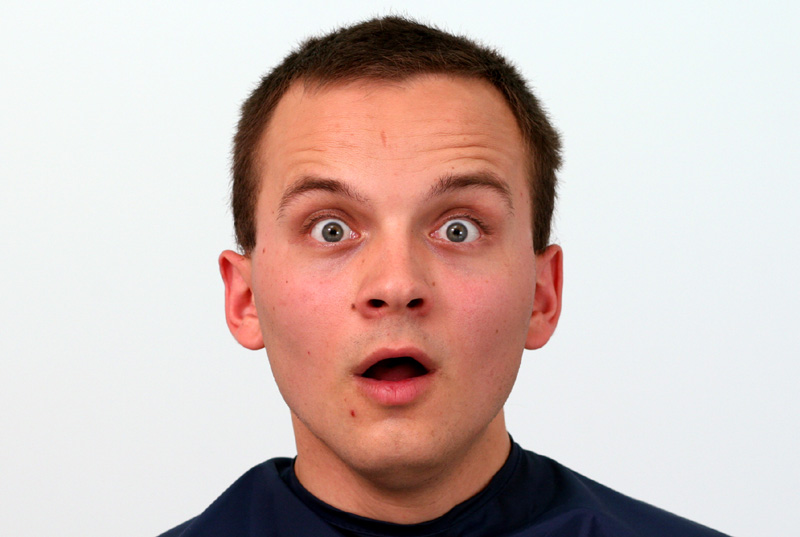

Supplement: Data Sheet 2 — WSEFEP - complete pictures dataset. [file DataSheet2.ZIP › MK_0255-lo.jpg]

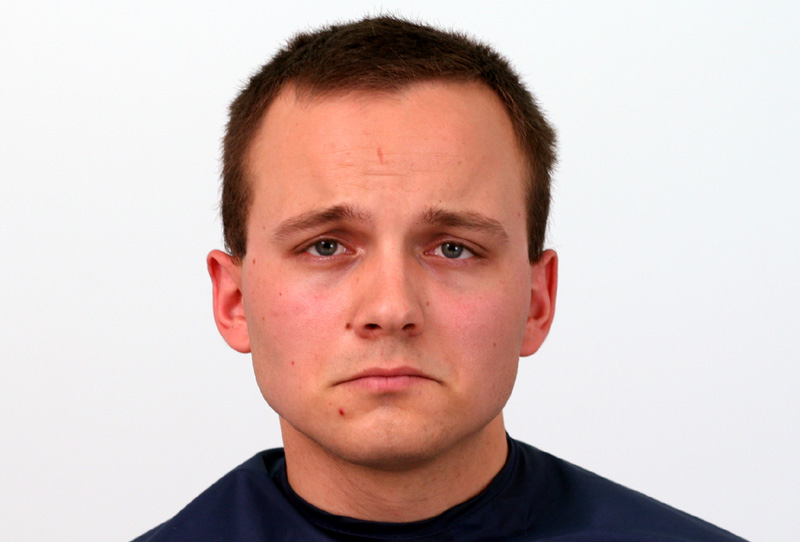

Supplement: Data Sheet 2 — WSEFEP - complete pictures dataset. [file DataSheet2.ZIP › MK_0306-lo.jpg]

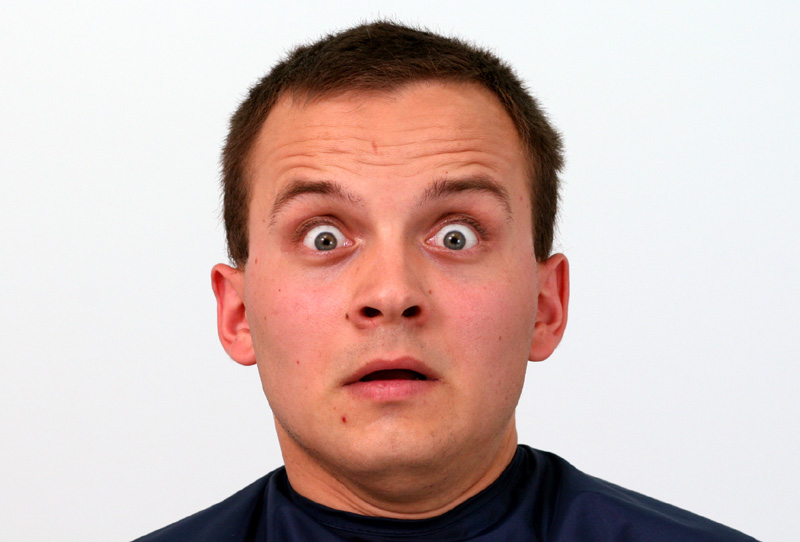

Supplement: Data Sheet 2 — WSEFEP - complete pictures dataset. [file DataSheet2.ZIP › MK_0364-lo.jpg]

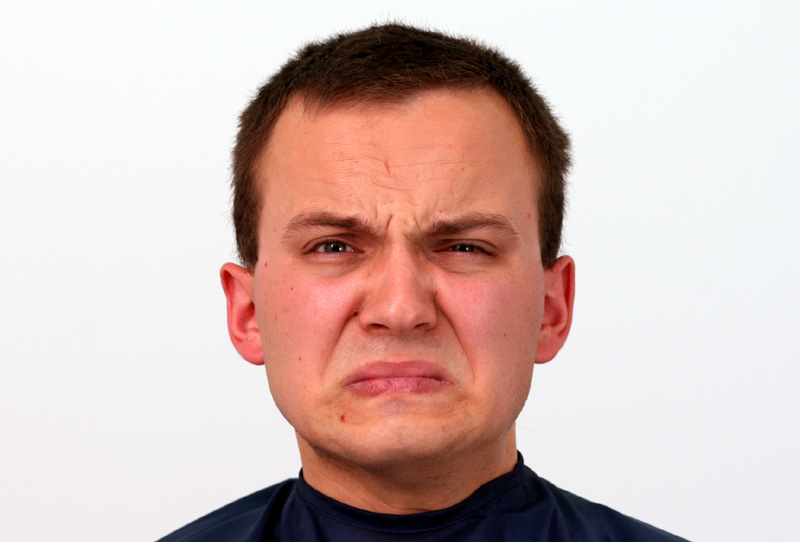

Supplement: Data Sheet 2 — WSEFEP - complete pictures dataset. [file DataSheet2.ZIP › MK_0496-lo.jpg]

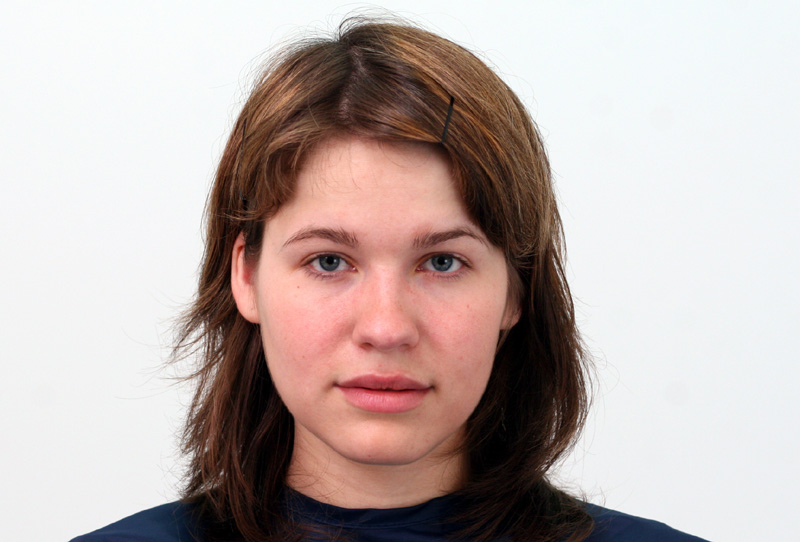

Supplement: Data Sheet 2 — WSEFEP - complete pictures dataset. [file DataSheet2.ZIP › MR1_0006-lo.jpg]

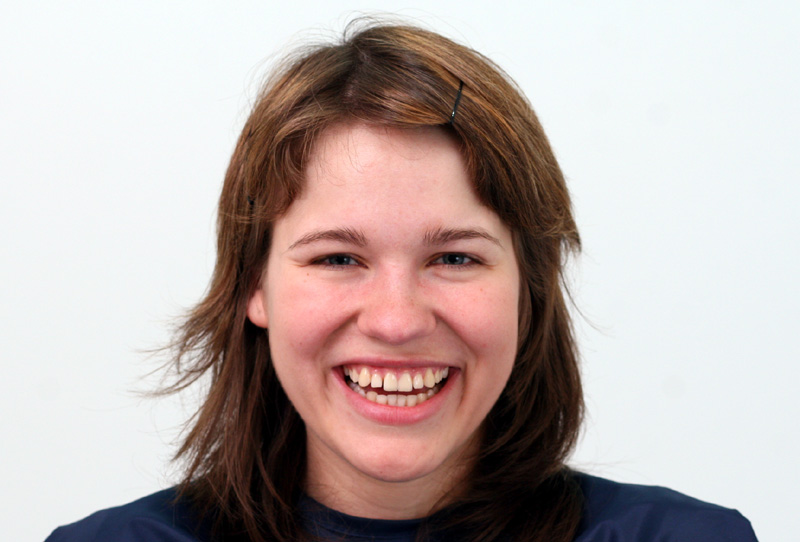

Supplement: Data Sheet 2 — WSEFEP - complete pictures dataset. [file DataSheet2.ZIP › MR1_0132-lo.jpg]

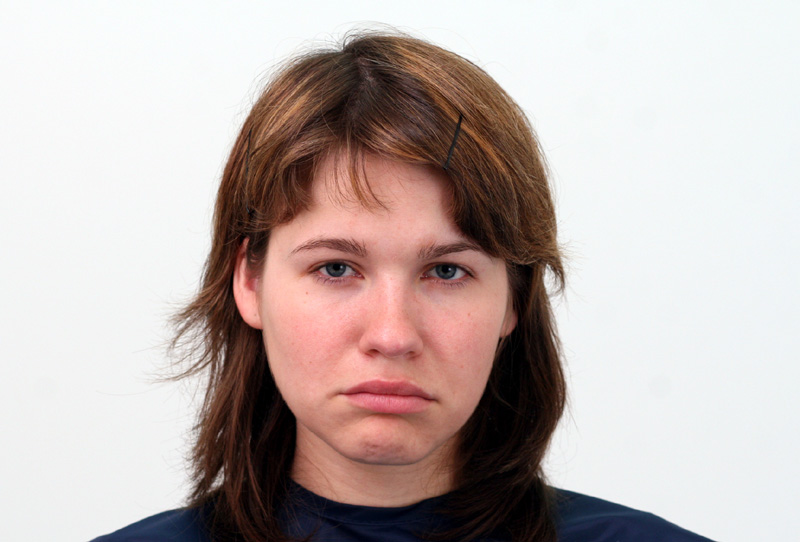

Supplement: Data Sheet 2 — WSEFEP - complete pictures dataset. [file DataSheet2.ZIP › MR1_0821-lo.jpg]

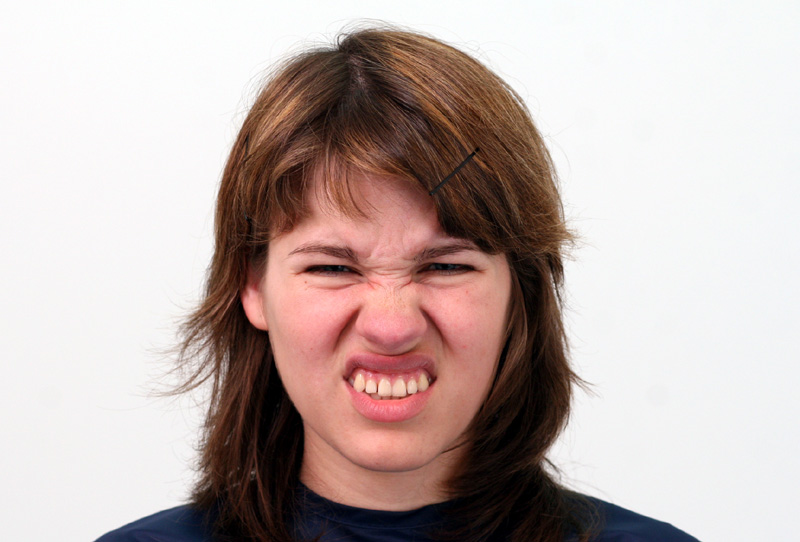

Supplement: Data Sheet 2 — WSEFEP - complete pictures dataset. [file DataSheet2.ZIP › MR1_1199-lo.jpg]

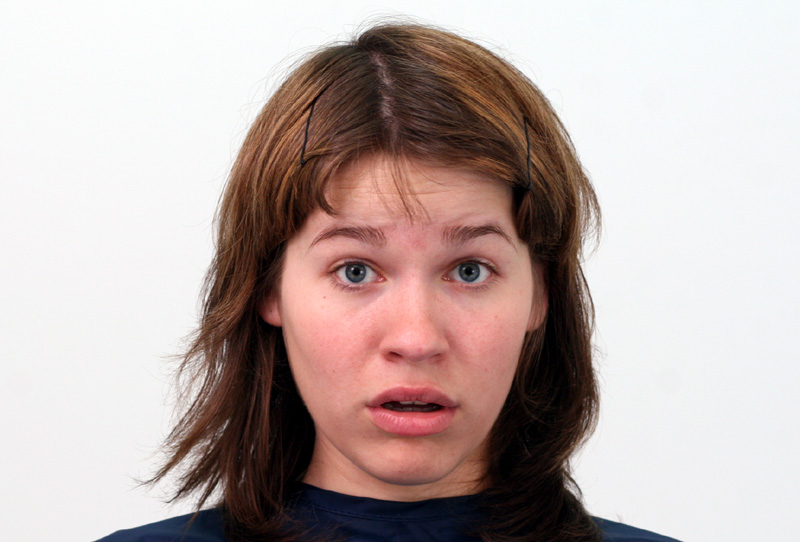

Supplement: Data Sheet 2 — WSEFEP - complete pictures dataset. [file DataSheet2.ZIP › MR1_1418-lo.jpg]

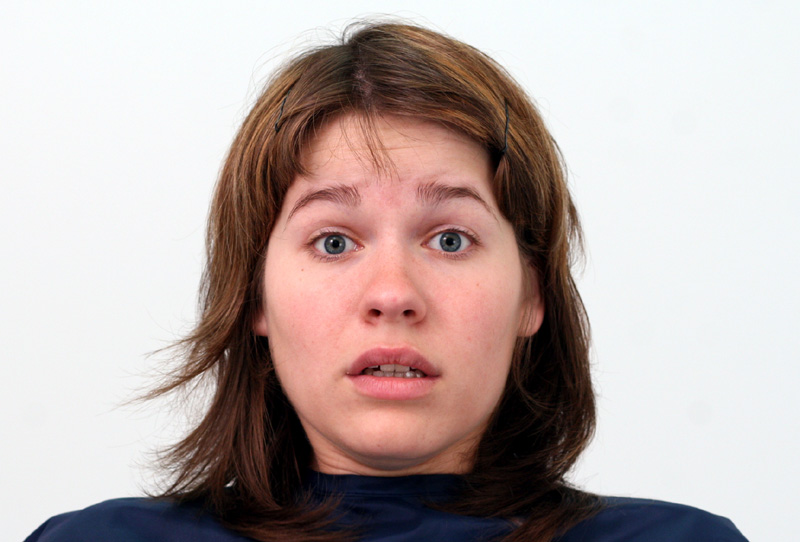

Supplement: Data Sheet 2 — WSEFEP - complete pictures dataset. [file DataSheet2.ZIP › MR1_1519-lo.jpg]

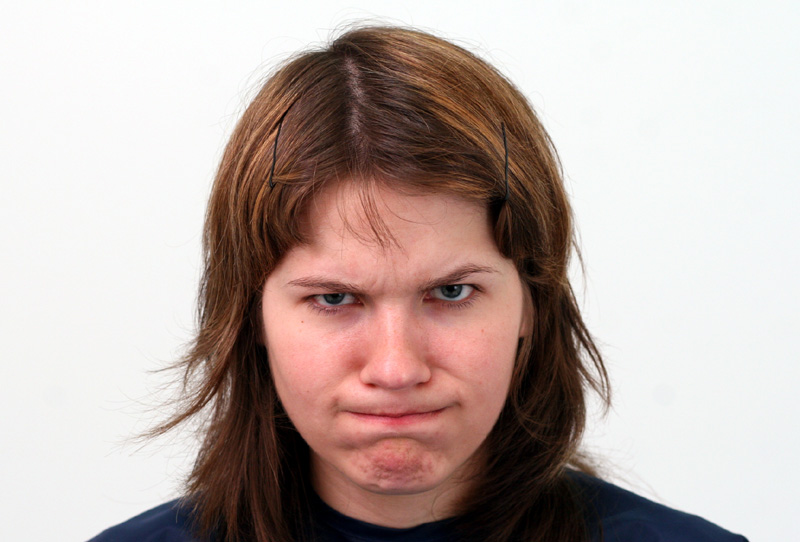

Supplement: Data Sheet 2 — WSEFEP - complete pictures dataset. [file DataSheet2.ZIP › MR1_1802-lo.jpg]

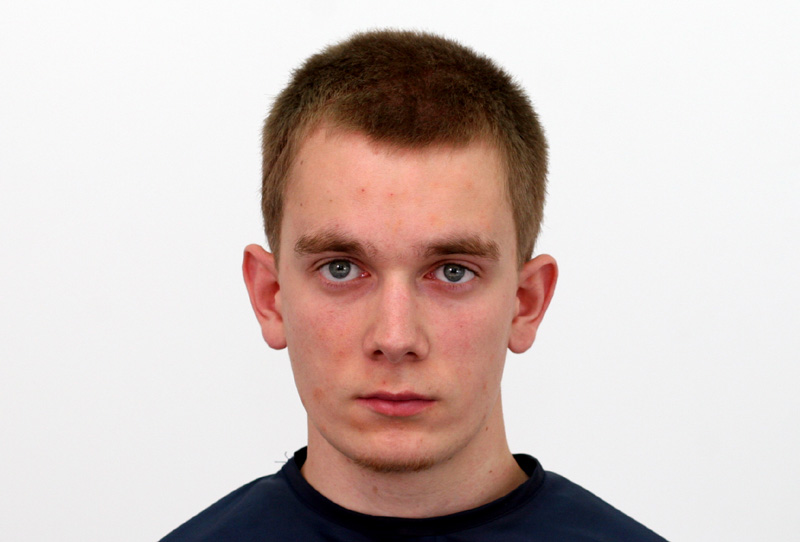

Supplement: Data Sheet 2 — WSEFEP - complete pictures dataset. [file DataSheet2.ZIP › MR2_0014-lo.jpg]

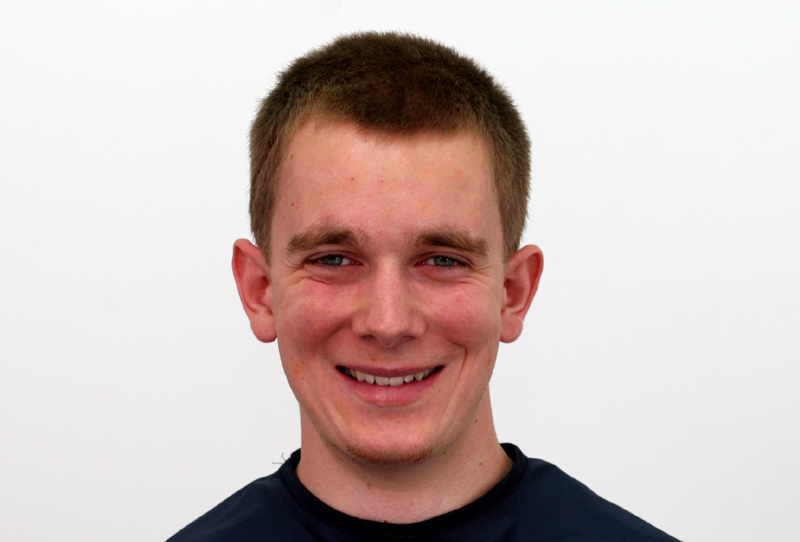

Supplement: Data Sheet 2 — WSEFEP - complete pictures dataset. [file DataSheet2.ZIP › MR2_0063-lo.jpg]

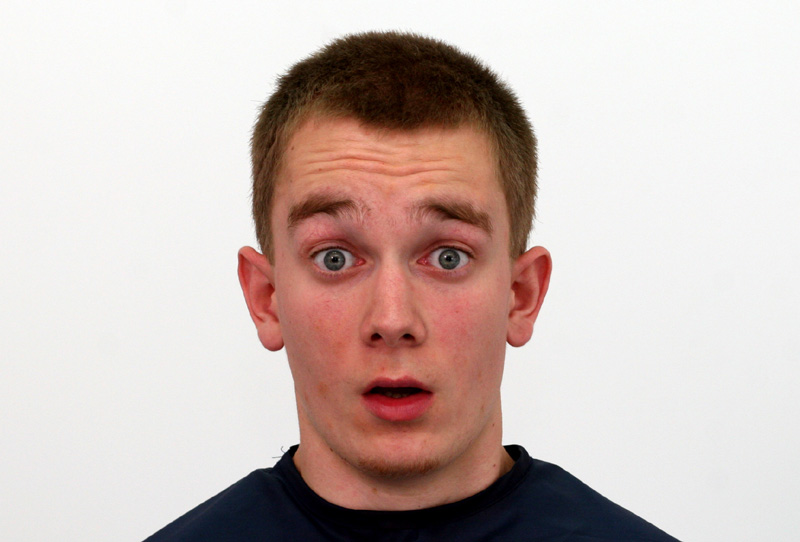

Supplement: Data Sheet 2 — WSEFEP - complete pictures dataset. [file DataSheet2.ZIP › MR2_0580-lo.jpg]

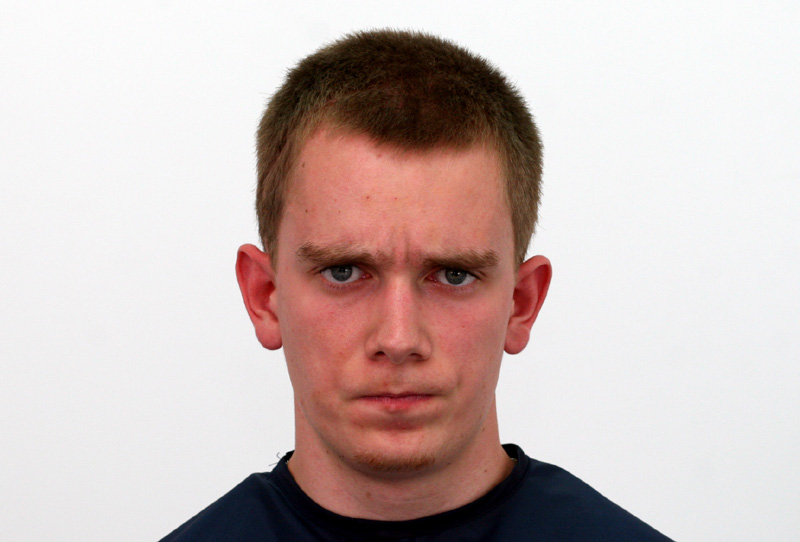

Supplement: Data Sheet 2 — WSEFEP - complete pictures dataset. [file DataSheet2.ZIP › MR2_0896-lo.jpg]

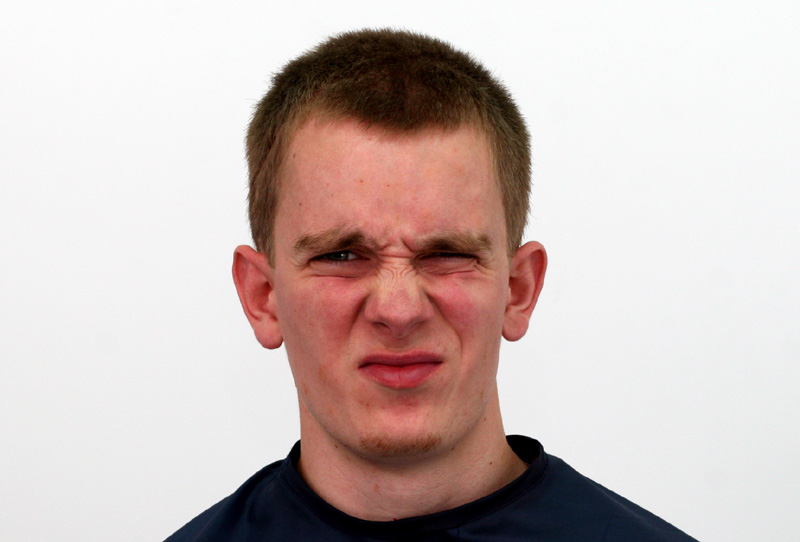

Supplement: Data Sheet 2 — WSEFEP - complete pictures dataset. [file DataSheet2.ZIP › MR2_1290-lo.jpg]

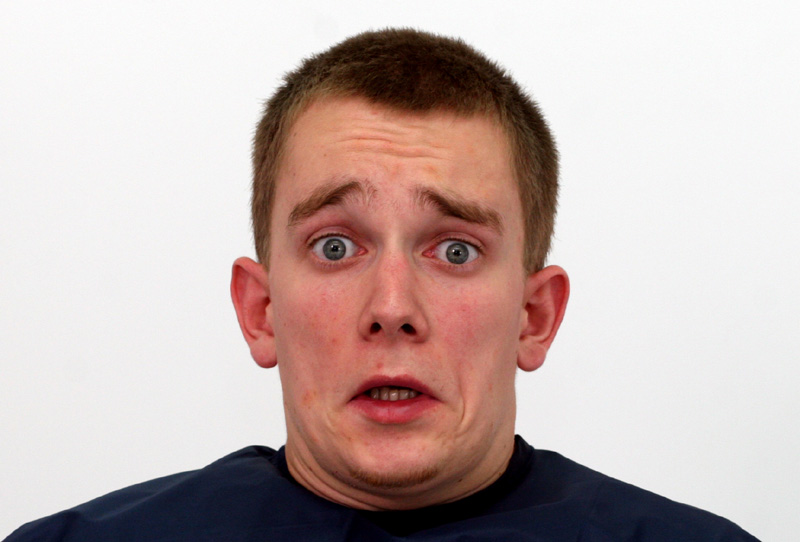

Supplement: Data Sheet 2 — WSEFEP - complete pictures dataset. [file DataSheet2.ZIP › MR2_1829-lo.jpg]

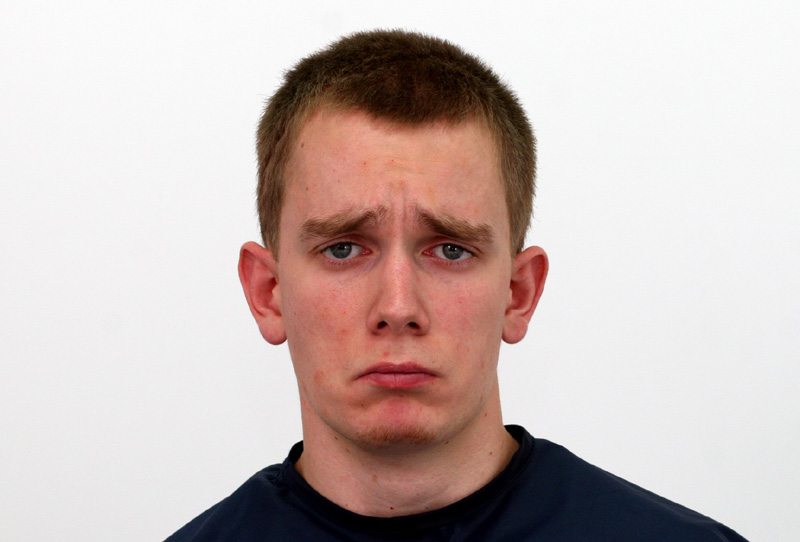

Supplement: Data Sheet 2 — WSEFEP - complete pictures dataset. [file DataSheet2.ZIP › MR2_2086-lo.jpg]

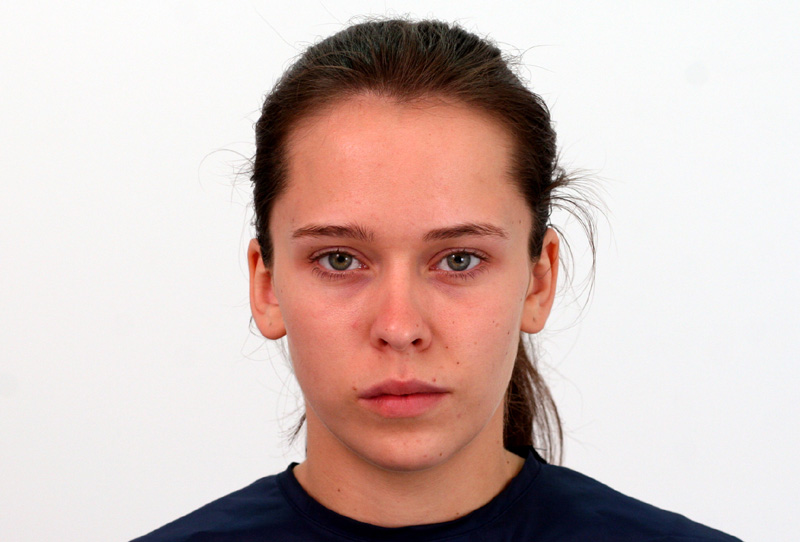

Supplement: Data Sheet 2 — WSEFEP - complete pictures dataset. [file DataSheet2.ZIP › MR_0013-lo.jpg]

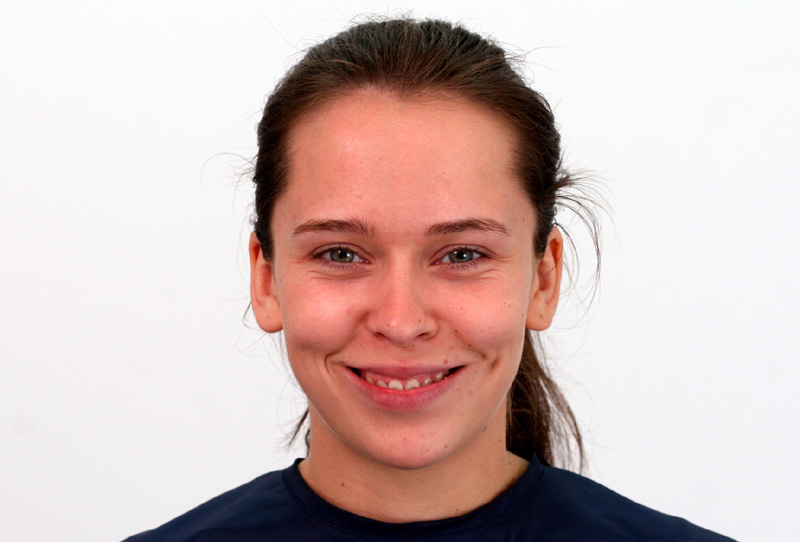

Supplement: Data Sheet 2 — WSEFEP - complete pictures dataset. [file DataSheet2.ZIP › MR_0055-lo.jpg]

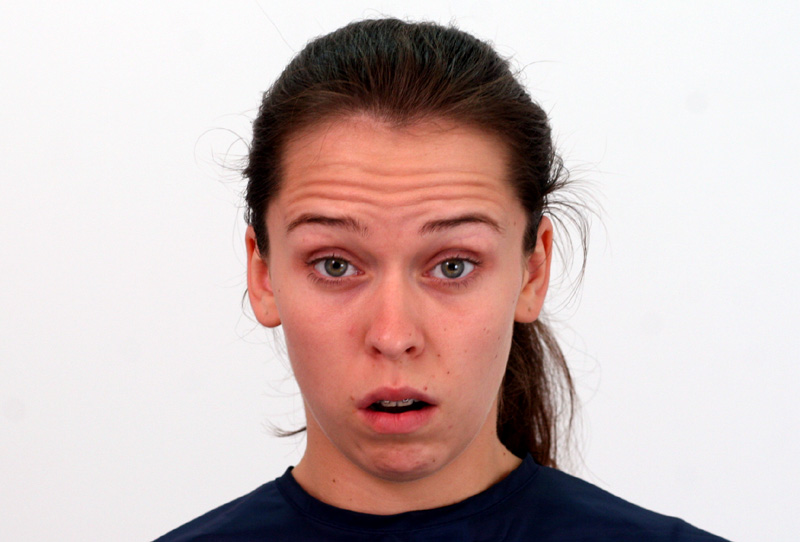

Supplement: Data Sheet 2 — WSEFEP - complete pictures dataset. [file DataSheet2.ZIP › MR_0619-lo.jpg]

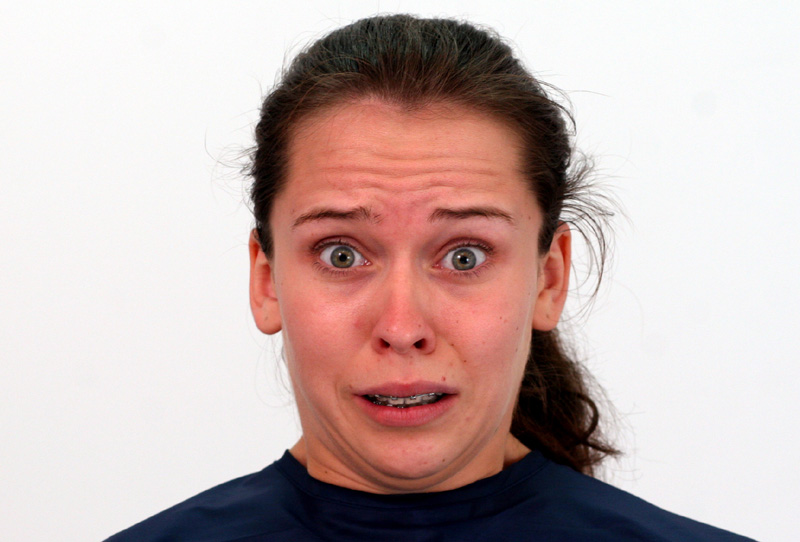

Supplement: Data Sheet 2 — WSEFEP - complete pictures dataset. [file DataSheet2.ZIP › MR_0959-lo.jpg]

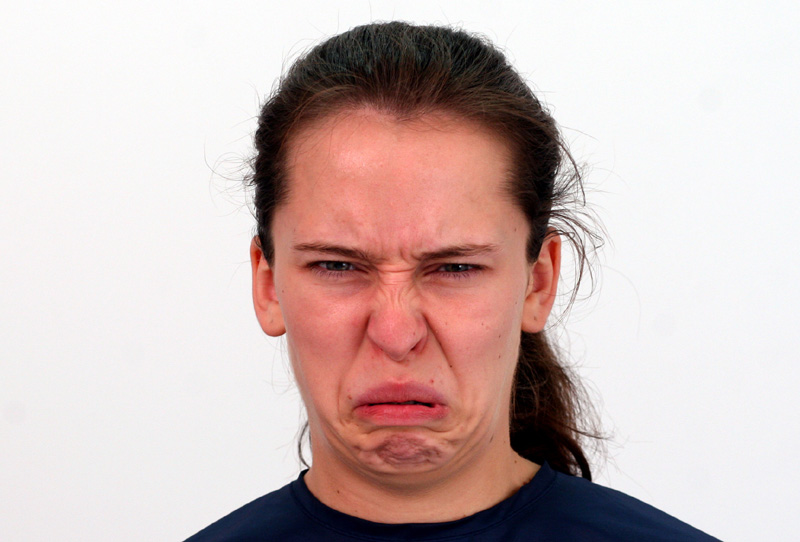

Supplement: Data Sheet 2 — WSEFEP - complete pictures dataset. [file DataSheet2.ZIP › MR_1669-lo.jpg]

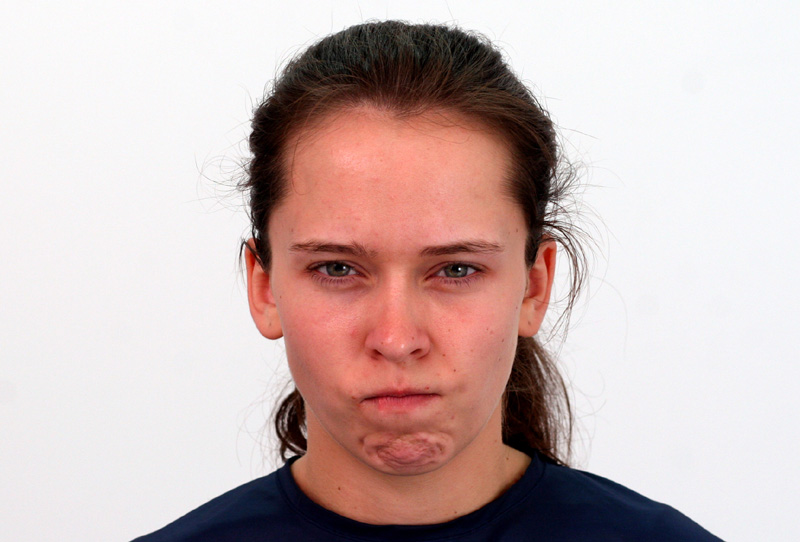

Supplement: Data Sheet 2 — WSEFEP - complete pictures dataset. [file DataSheet2.ZIP › MR_2450-lo.jpg]

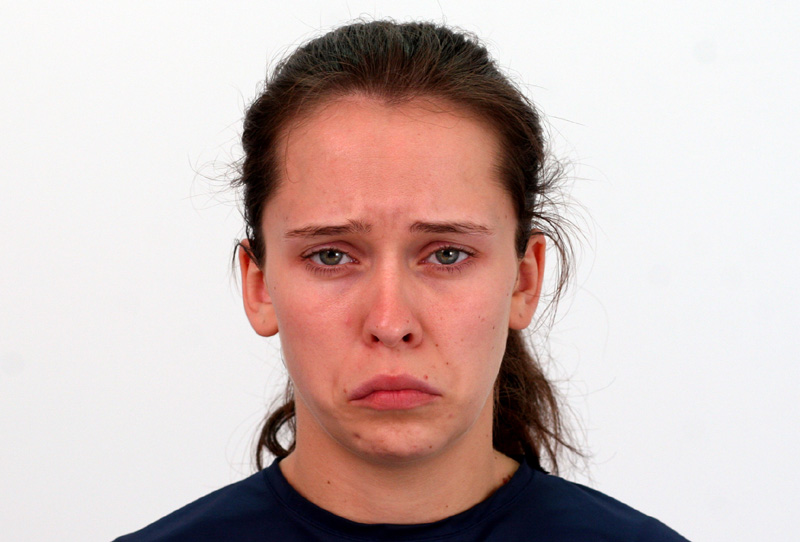

Supplement: Data Sheet 2 — WSEFEP - complete pictures dataset. [file DataSheet2.ZIP › MR_2767-lo.jpg]

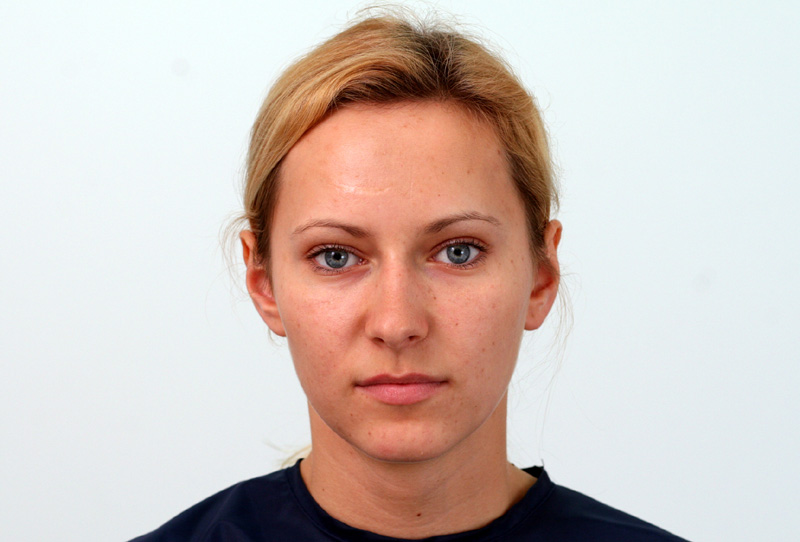

Supplement: Data Sheet 2 — WSEFEP - complete pictures dataset. [file DataSheet2.ZIP › MS_0004-lo.jpg]

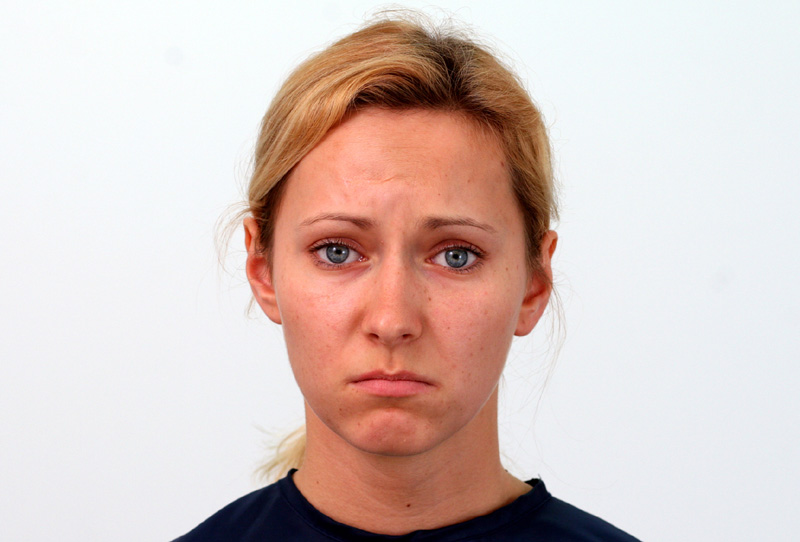

Supplement: Data Sheet 2 — WSEFEP - complete pictures dataset. [file DataSheet2.ZIP › MS_0104-lo.jpg]

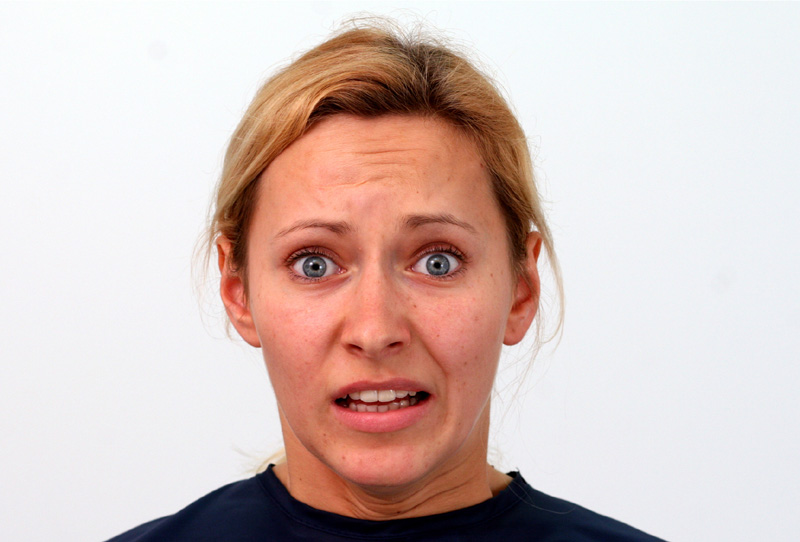

Supplement: Data Sheet 2 — WSEFEP - complete pictures dataset. [file DataSheet2.ZIP › MS_0226-lo.jpg]

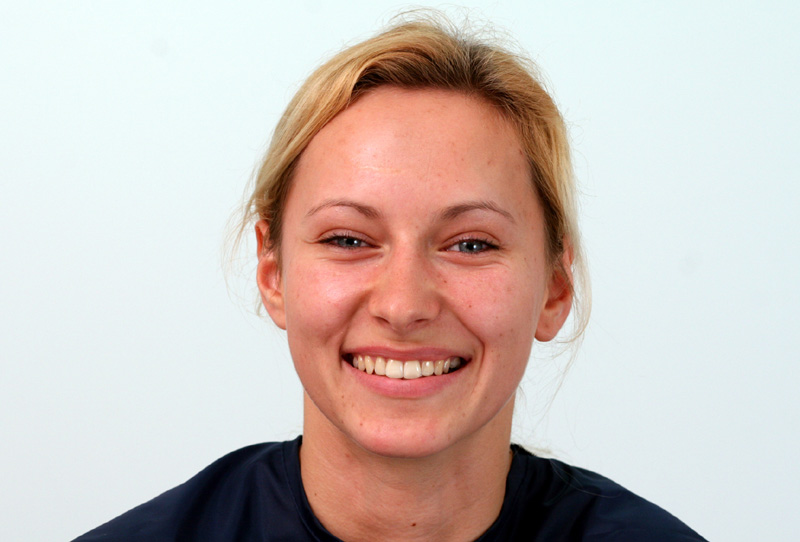

Supplement: Data Sheet 2 — WSEFEP - complete pictures dataset. [file DataSheet2.ZIP › MS_0291-lo.jpg]

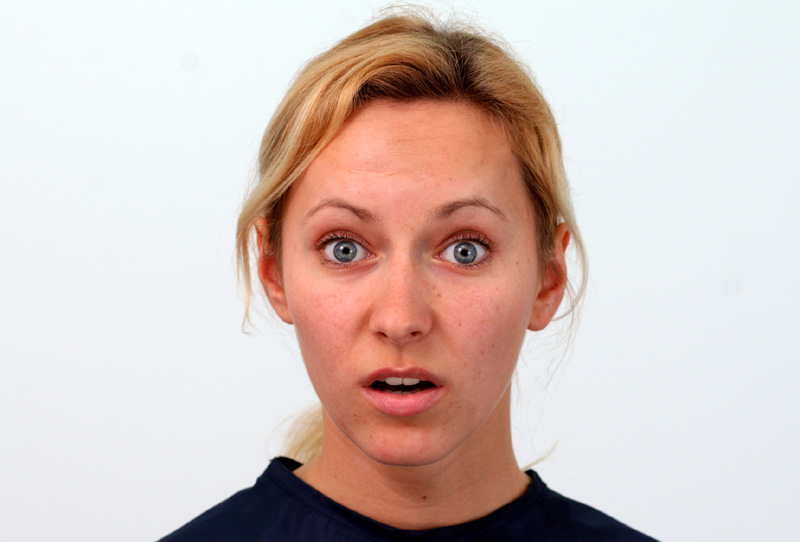

Supplement: Data Sheet 2 — WSEFEP - complete pictures dataset. [file DataSheet2.ZIP › MS_0431-lo.jpg]

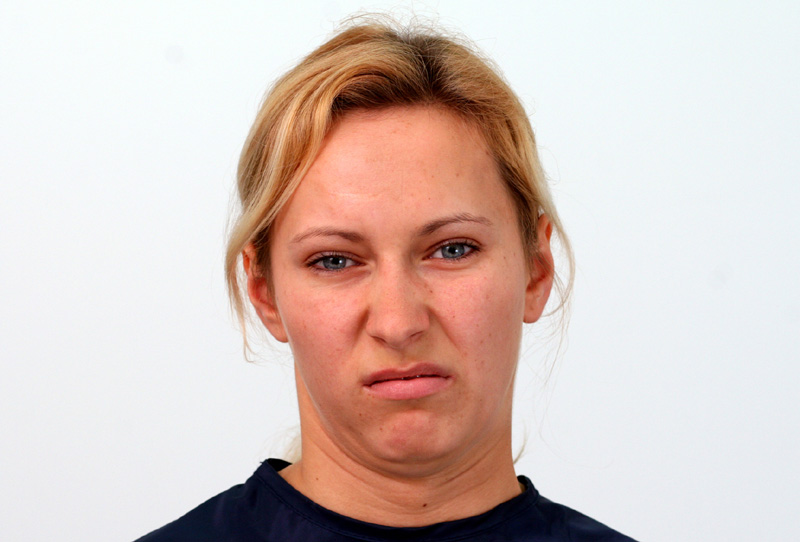

Supplement: Data Sheet 2 — WSEFEP - complete pictures dataset. [file DataSheet2.ZIP › MS_0627-lo.jpg]

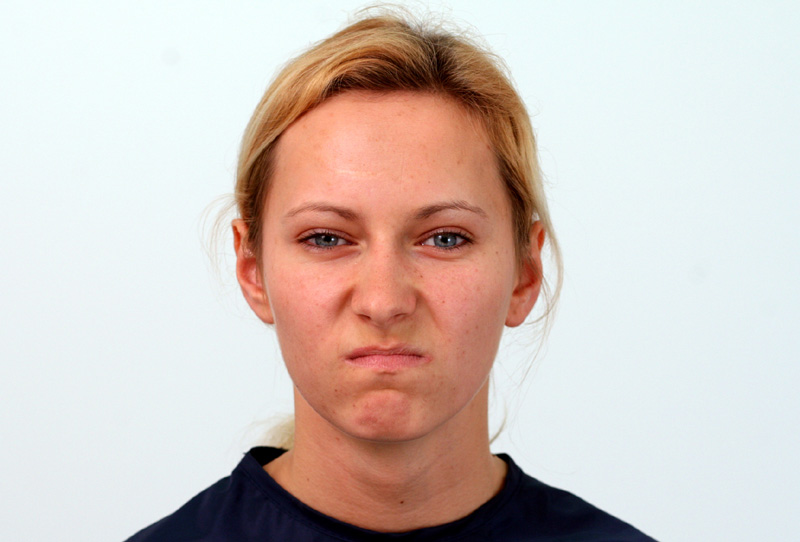

Supplement: Data Sheet 2 — WSEFEP - complete pictures dataset. [file DataSheet2.ZIP › MS_0695-lo.jpg]

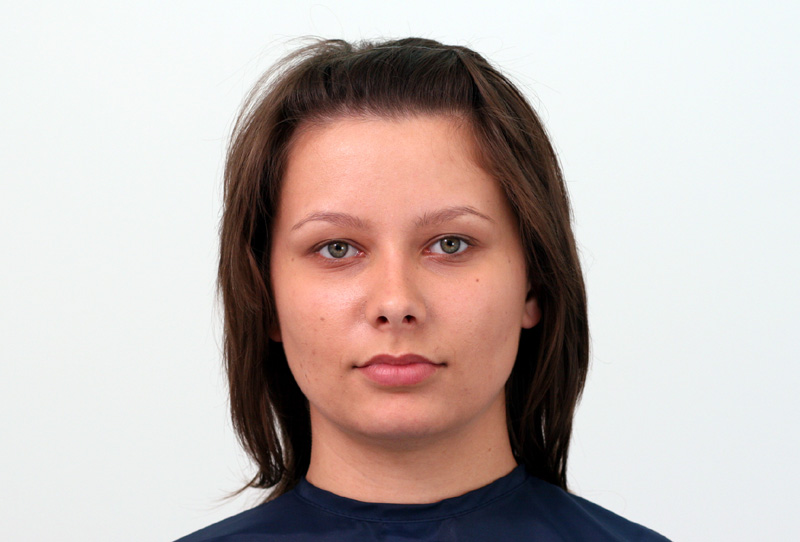

Supplement: Data Sheet 2 — WSEFEP - complete pictures dataset. [file DataSheet2.ZIP › OG_6108-lo.jpg]

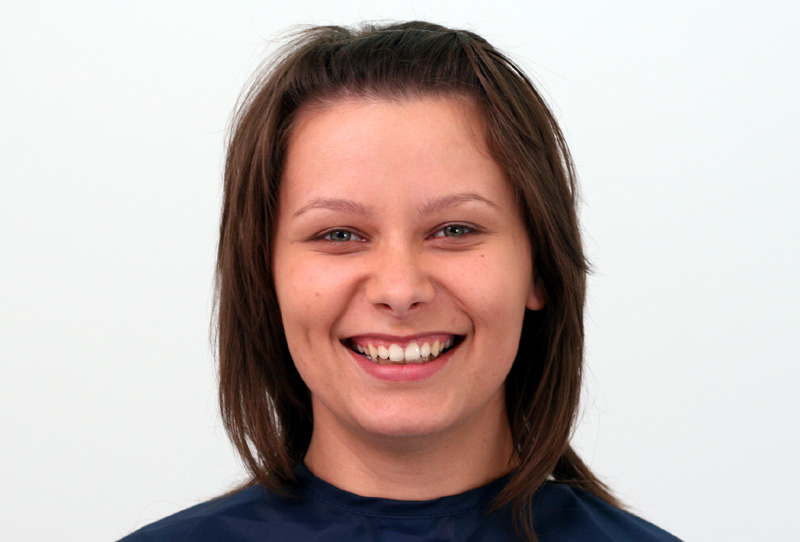

Supplement: Data Sheet 2 — WSEFEP - complete pictures dataset. [file DataSheet2.ZIP › OG_6189-lo.jpg]

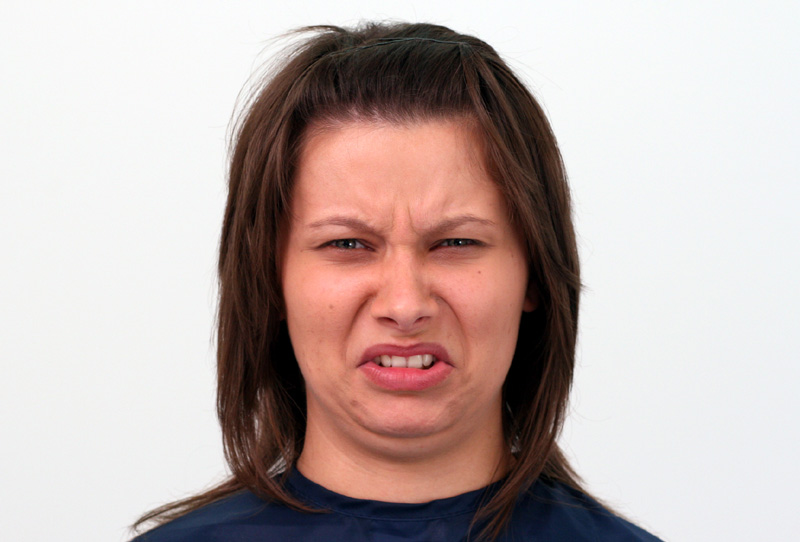

Supplement: Data Sheet 2 — WSEFEP - complete pictures dataset. [file DataSheet2.ZIP › OG_6390-lo.jpg]

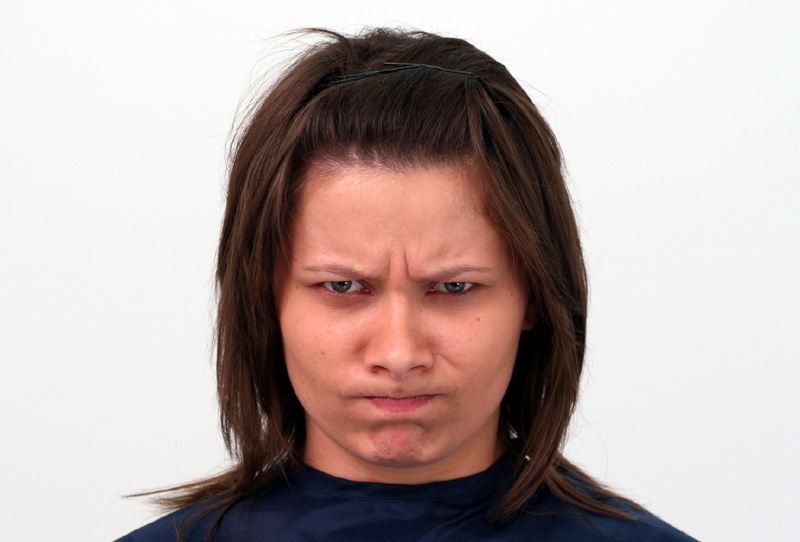

Supplement: Data Sheet 2 — WSEFEP - complete pictures dataset. [file DataSheet2.ZIP › OG_6484-lo.jpg]

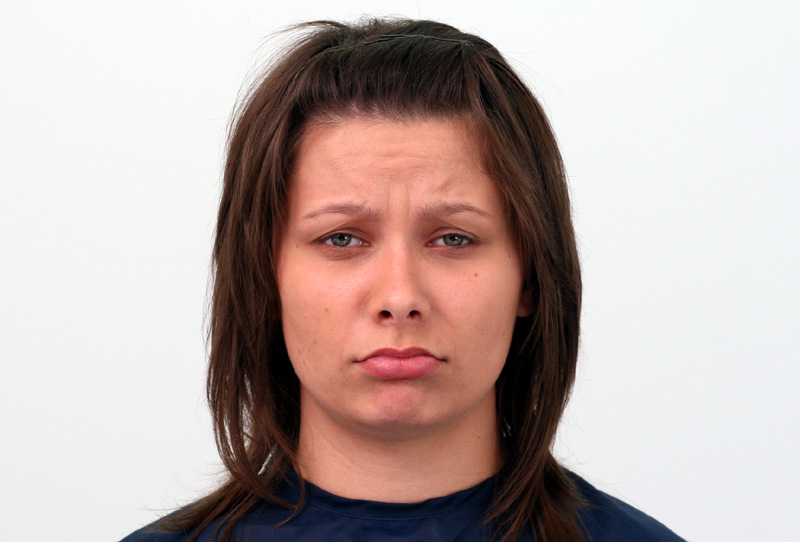

Supplement: Data Sheet 2 — WSEFEP - complete pictures dataset. [file DataSheet2.ZIP › OG_6566-lo.jpg]

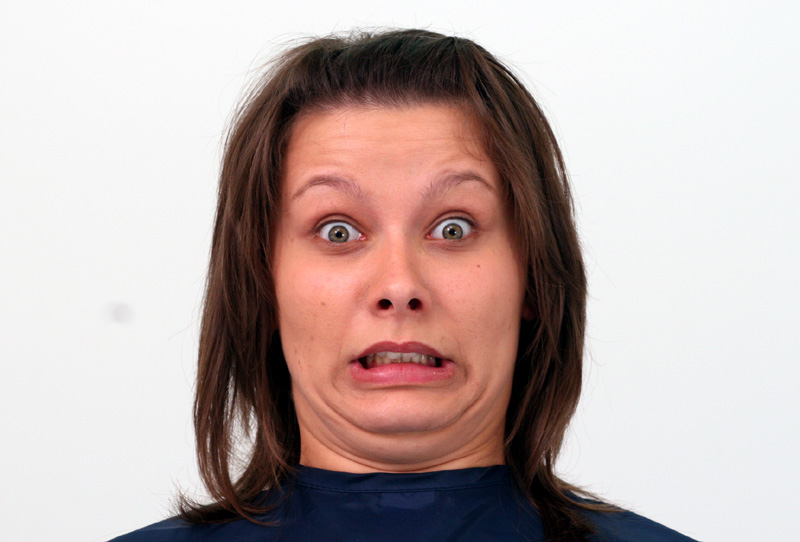

Supplement: Data Sheet 2 — WSEFEP - complete pictures dataset. [file DataSheet2.ZIP › OG_7620-lo.jpg]

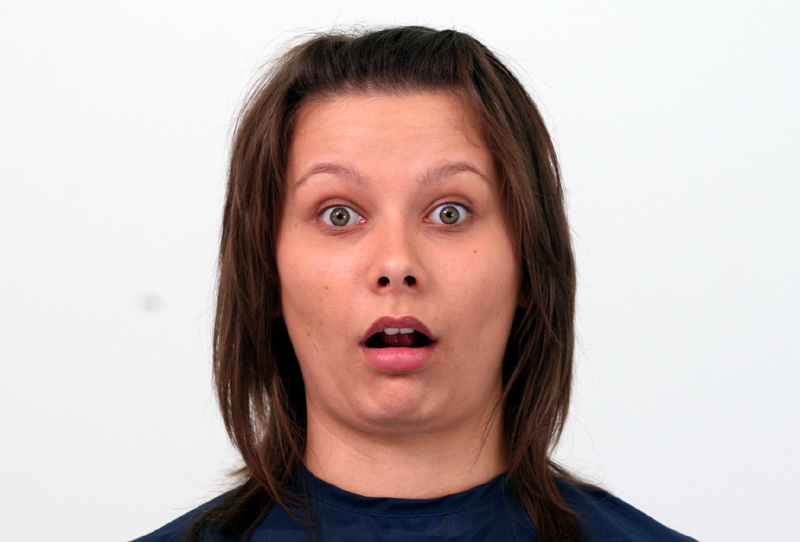

Supplement: Data Sheet 2 — WSEFEP - complete pictures dataset. [file DataSheet2.ZIP › OG_7702-lo.jpg]

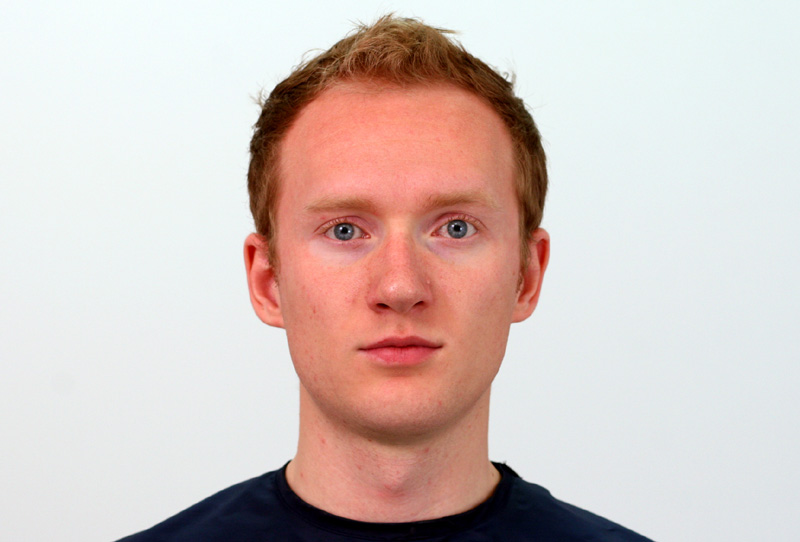

Supplement: Data Sheet 2 — WSEFEP - complete pictures dataset. [file DataSheet2.ZIP › PA_0006-lo.jpg]

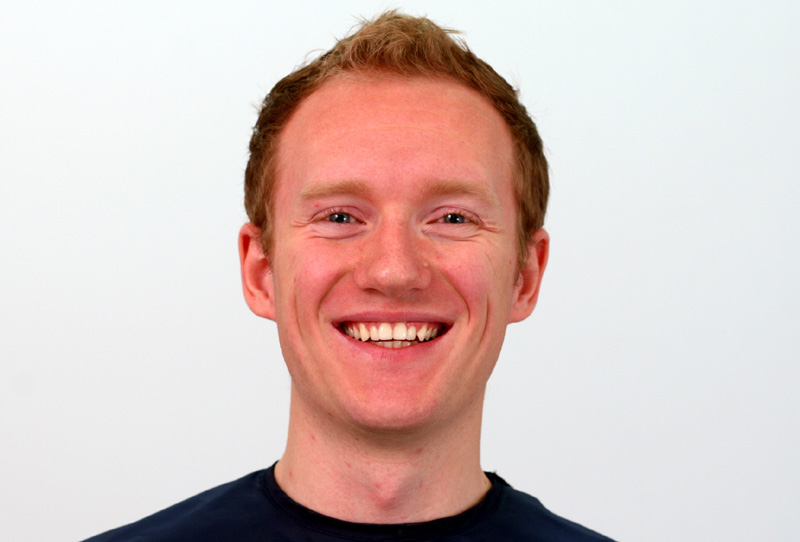

Supplement: Data Sheet 2 — WSEFEP - complete pictures dataset. [file DataSheet2.ZIP › PA_0112-lo.jpg]

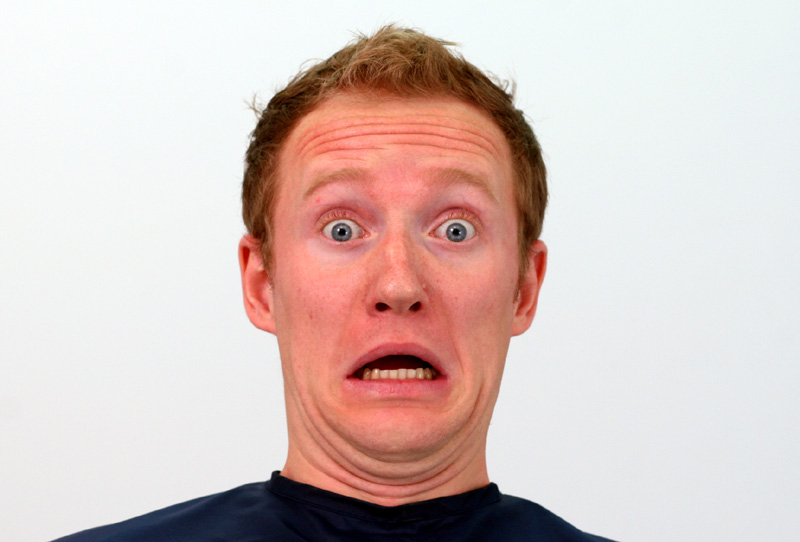

Supplement: Data Sheet 2 — WSEFEP - complete pictures dataset. [file DataSheet2.ZIP › PA_0394-lo.jpg]

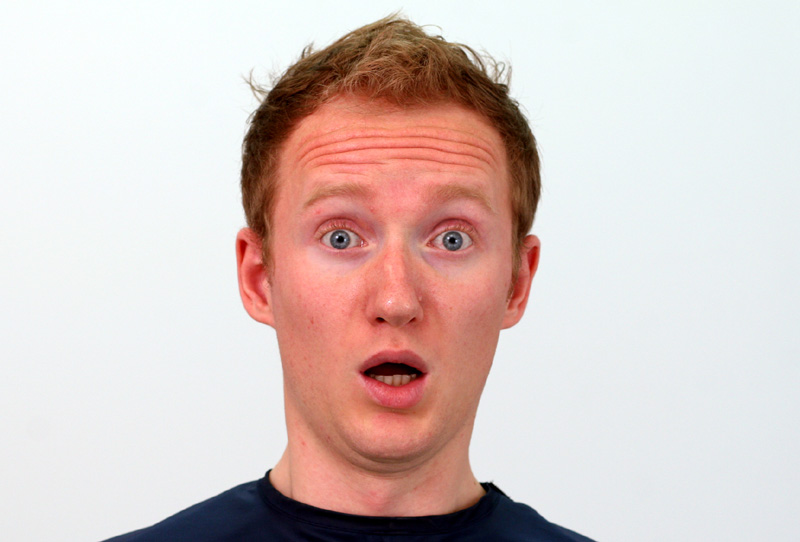

Supplement: Data Sheet 2 — WSEFEP - complete pictures dataset. [file DataSheet2.ZIP › PA_0840-lo.jpg]

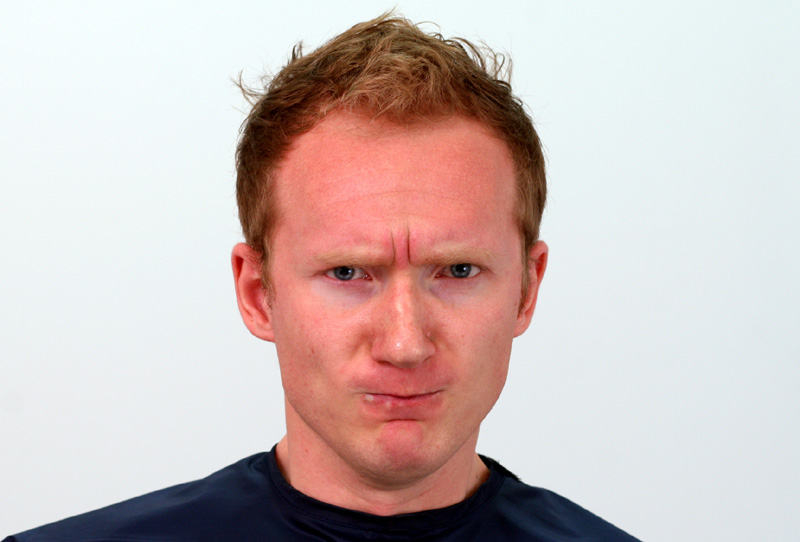

Supplement: Data Sheet 2 — WSEFEP - complete pictures dataset. [file DataSheet2.ZIP › PA_0967-lo.jpg]

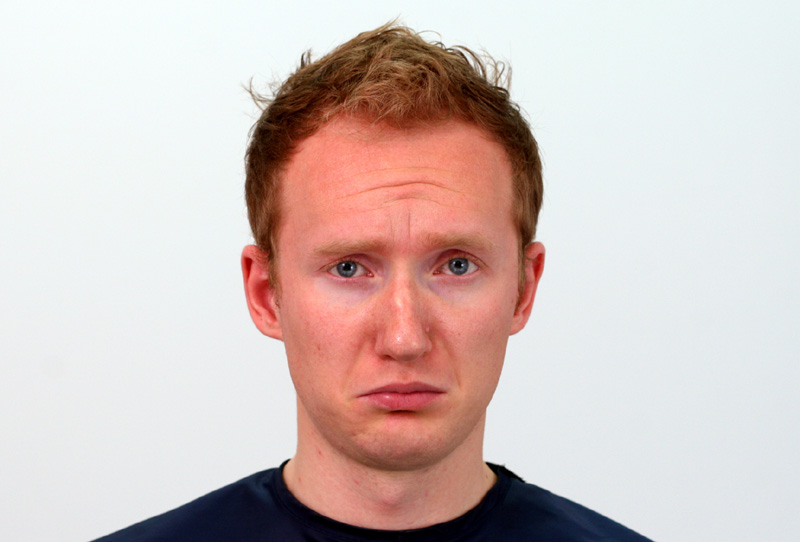

Supplement: Data Sheet 2 — WSEFEP - complete pictures dataset. [file DataSheet2.ZIP › PA_1348-lo.jpg]

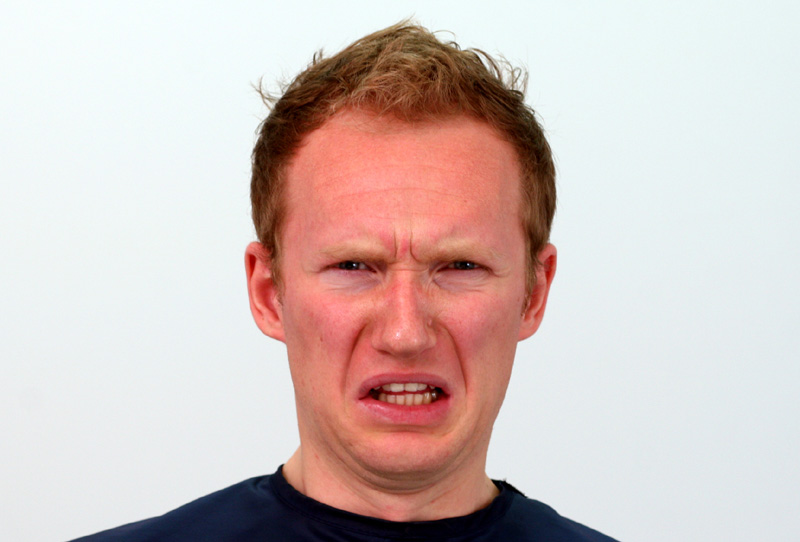

Supplement: Data Sheet 2 — WSEFEP - complete pictures dataset. [file DataSheet2.ZIP › PA_1701-lo.jpg]

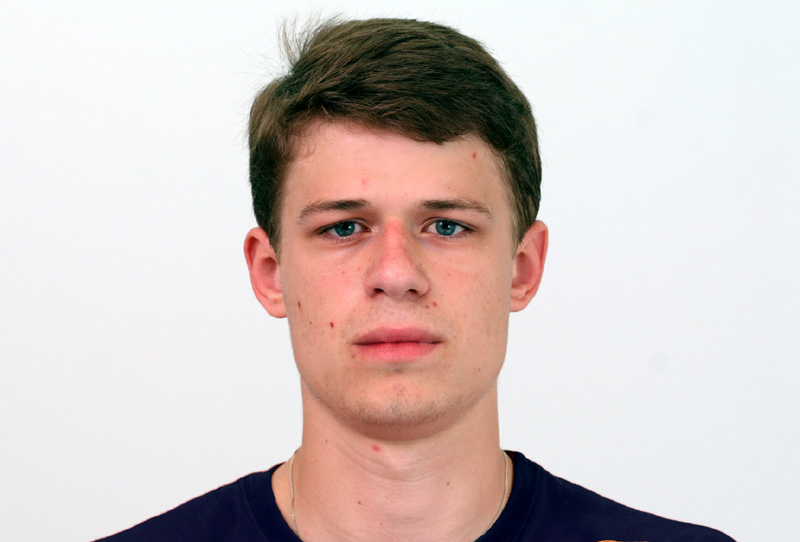

Supplement: Data Sheet 2 — WSEFEP - complete pictures dataset. [file DataSheet2.ZIP › PB_0001-lo.jpg]

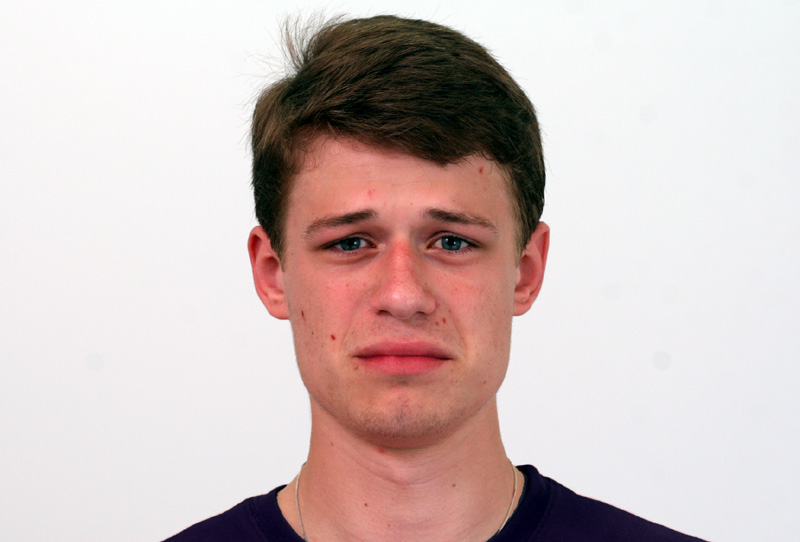

Supplement: Data Sheet 2 — WSEFEP - complete pictures dataset. [file DataSheet2.ZIP › PB_0144-lo.jpg]

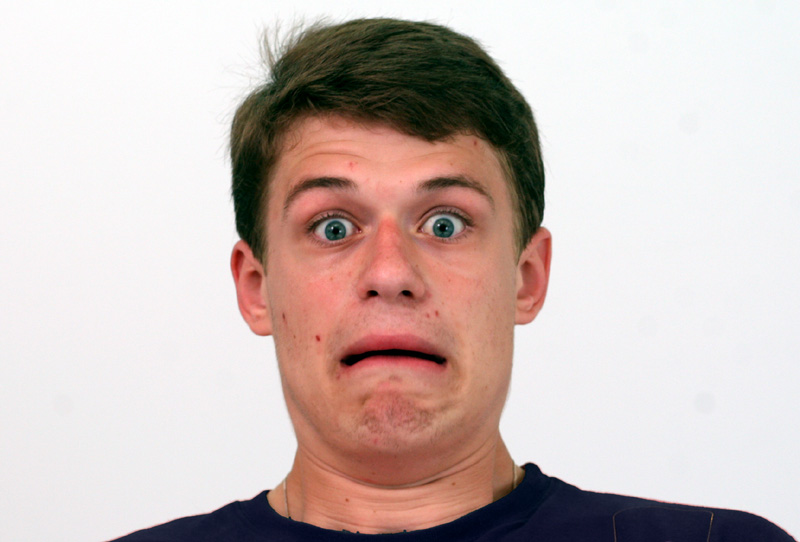

Supplement: Data Sheet 2 — WSEFEP - complete pictures dataset. [file DataSheet2.ZIP › PB_0269-lo.jpg]

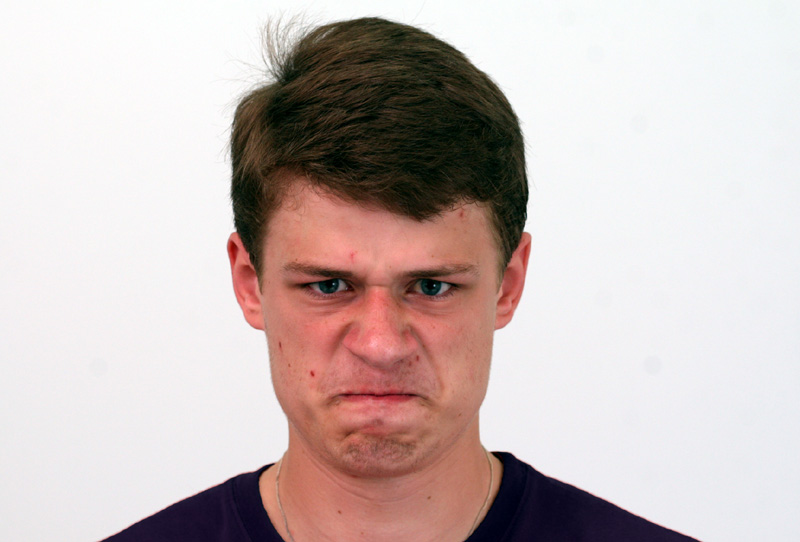

Supplement: Data Sheet 2 — WSEFEP - complete pictures dataset. [file DataSheet2.ZIP › PB_0314-lo.jpg]

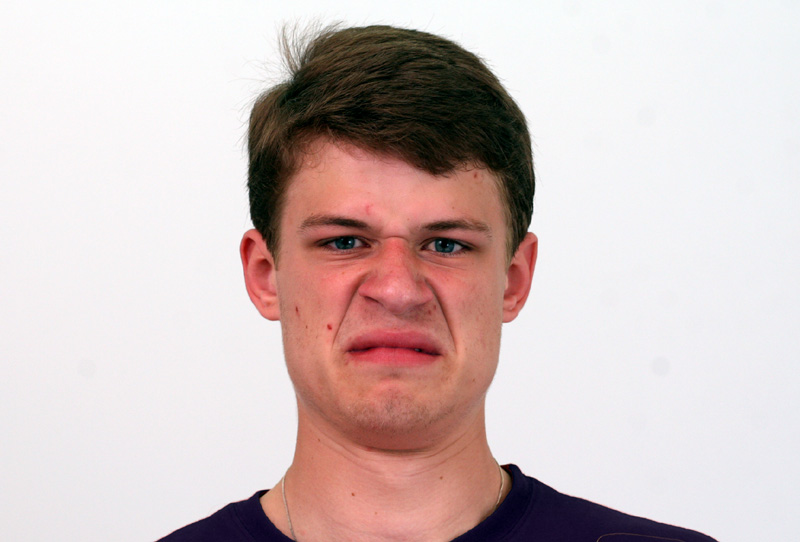

Supplement: Data Sheet 2 — WSEFEP - complete pictures dataset. [file DataSheet2.ZIP › PB_0442-lo.jpg]

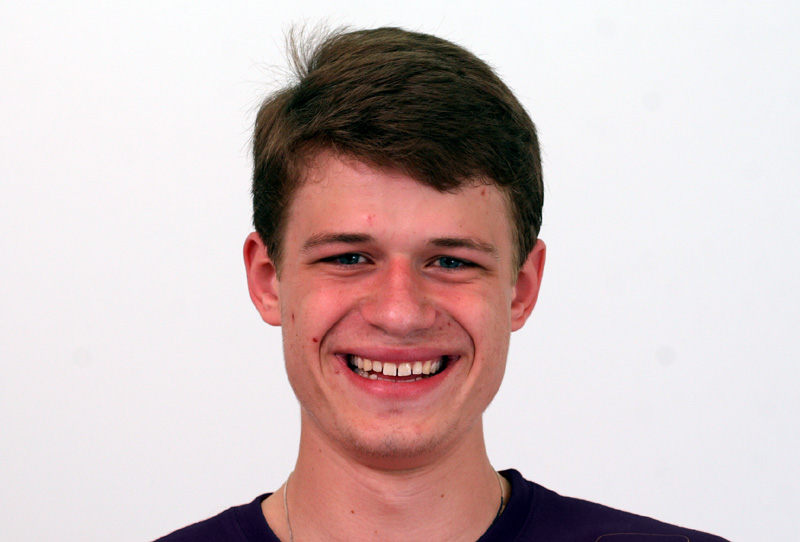

Supplement: Data Sheet 2 — WSEFEP - complete pictures dataset. [file DataSheet2.ZIP › PB_0499-lo.jpg]

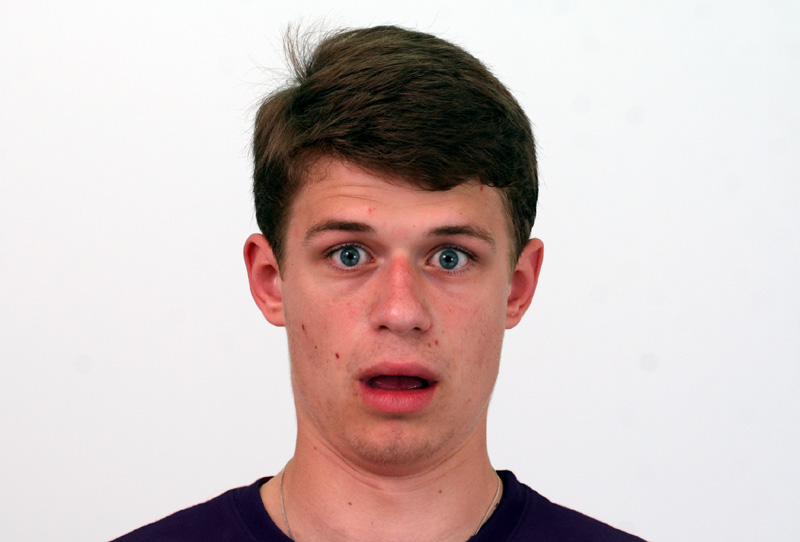

Supplement: Data Sheet 2 — WSEFEP - complete pictures dataset. [file DataSheet2.ZIP › PB_1383-lo.jpg]

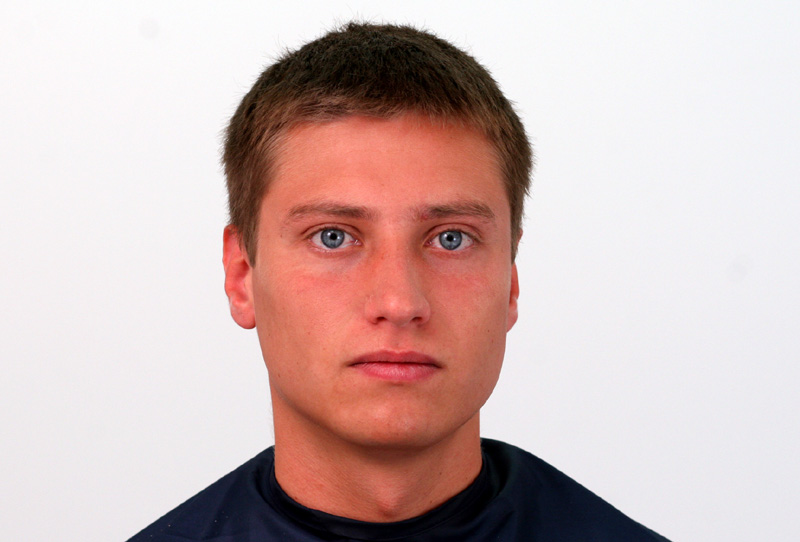

Supplement: Data Sheet 2 — WSEFEP - complete pictures dataset. [file DataSheet2.ZIP › PO_0015-lo.jpg]

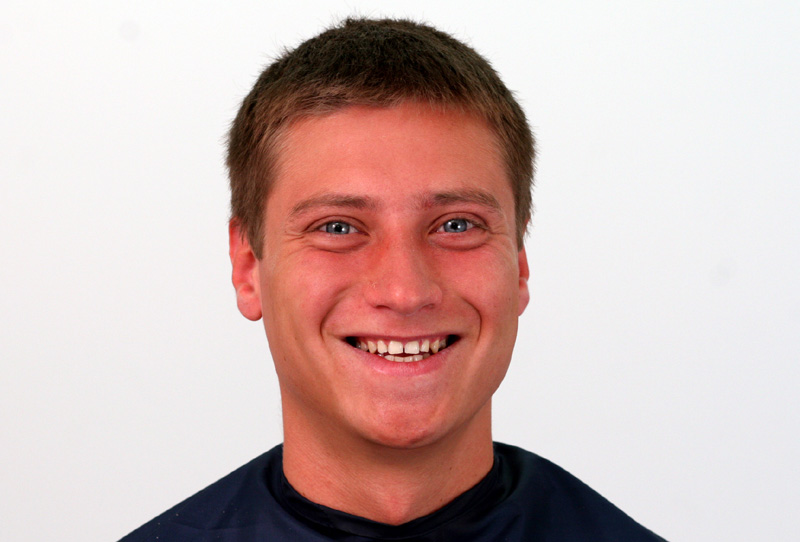

Supplement: Data Sheet 2 — WSEFEP - complete pictures dataset. [file DataSheet2.ZIP › PO_0124-lo.jpg]

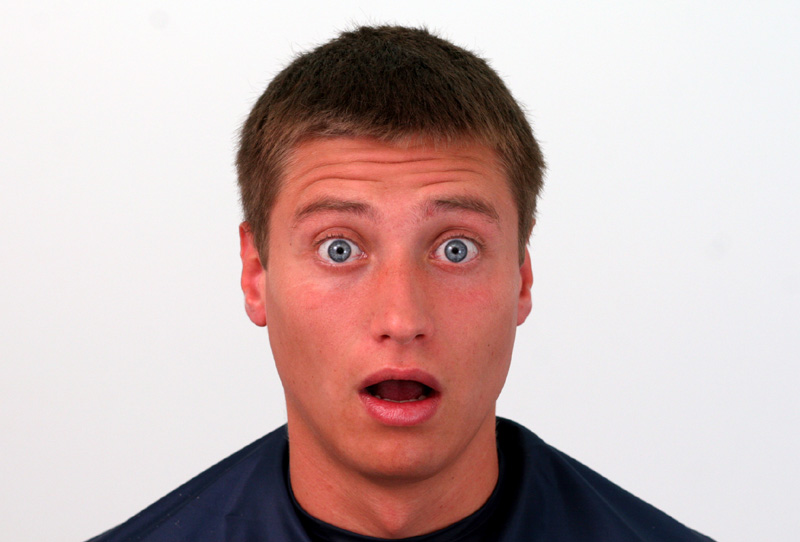

Supplement: Data Sheet 2 — WSEFEP - complete pictures dataset. [file DataSheet2.ZIP › PO_0553-lo.jpg]

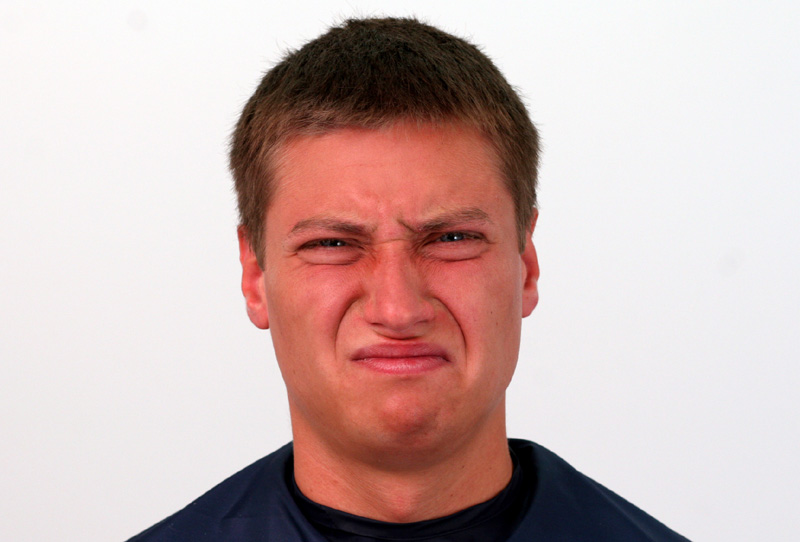

Supplement: Data Sheet 2 — WSEFEP - complete pictures dataset. [file DataSheet2.ZIP › PO_0673-lo.jpg]

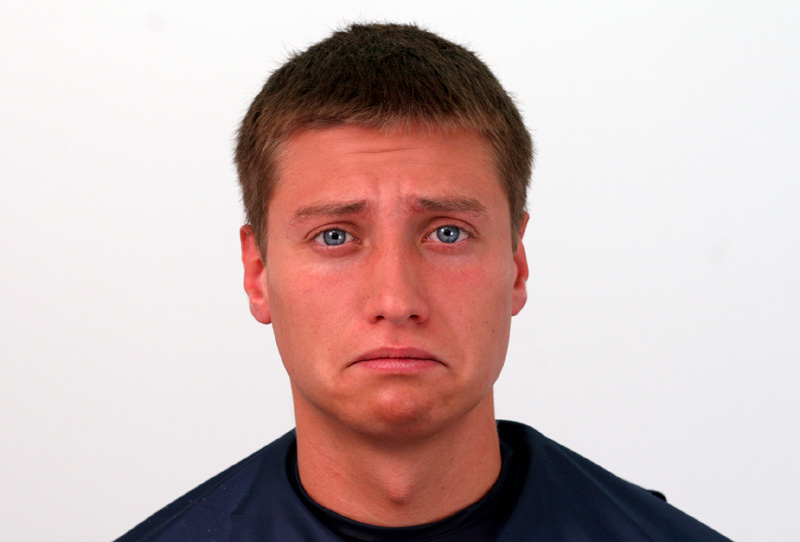

Supplement: Data Sheet 2 — WSEFEP - complete pictures dataset. [file DataSheet2.ZIP › PO_0843-lo.jpg]

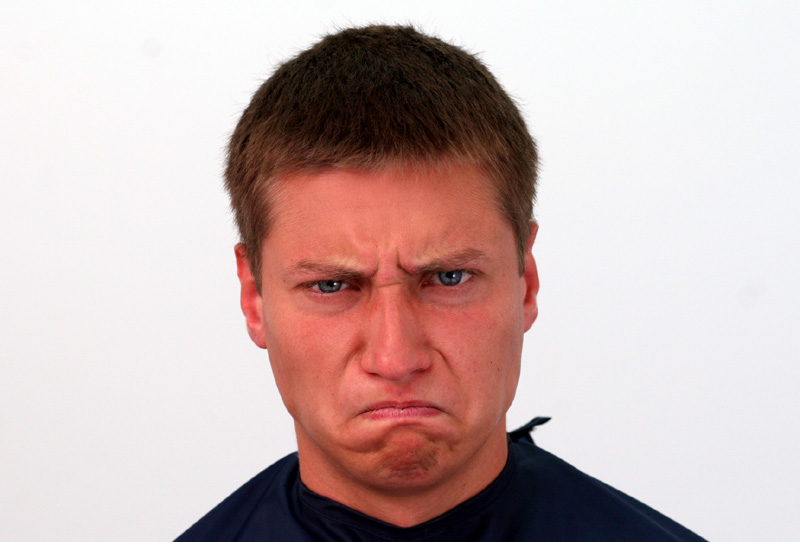

Supplement: Data Sheet 2 — WSEFEP - complete pictures dataset. [file DataSheet2.ZIP › PO_0951-lo.jpg]

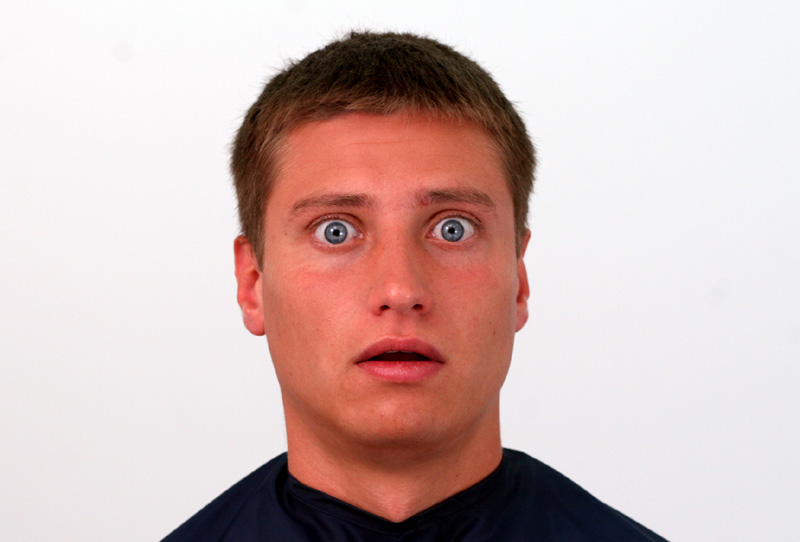

Supplement: Data Sheet 2 — WSEFEP - complete pictures dataset. [file DataSheet2.ZIP › PO_1030-lo.jpg]

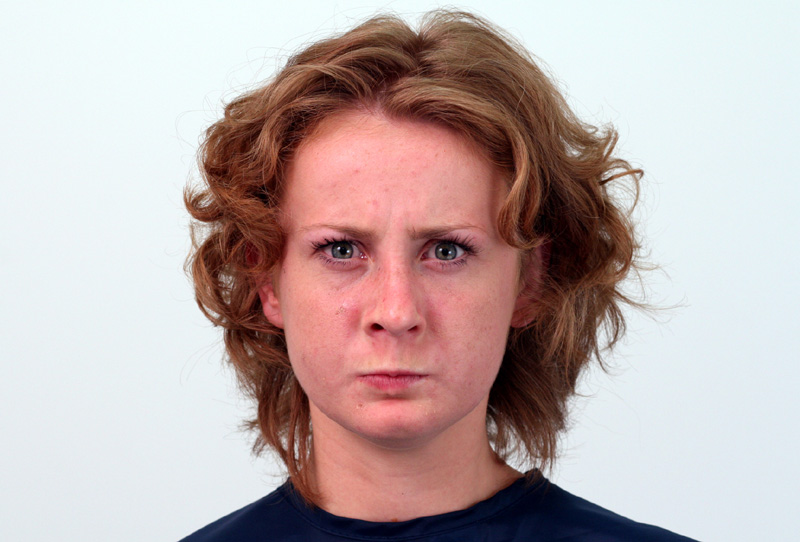

Supplement: Data Sheet 2 — WSEFEP - complete pictures dataset. [file DataSheet2.ZIP › PS_0100-lo.jpg]

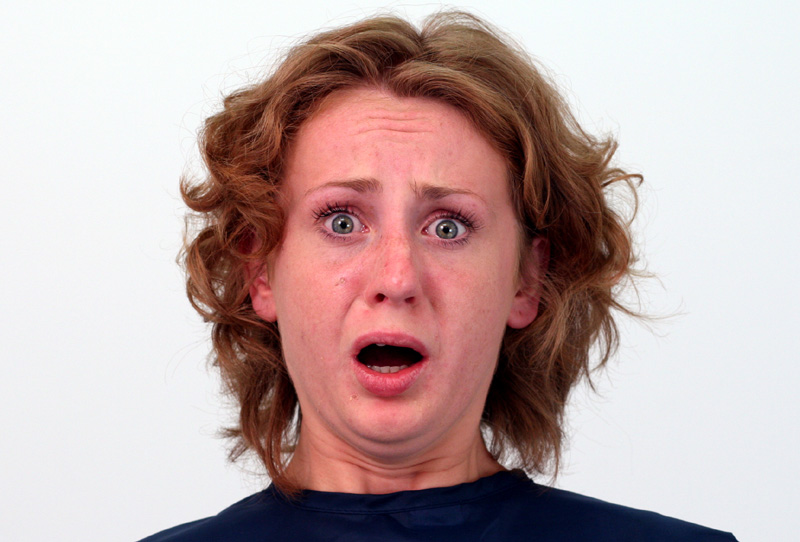

Supplement: Data Sheet 2 — WSEFEP - complete pictures dataset. [file DataSheet2.ZIP › PS_0157-lo.jpg]

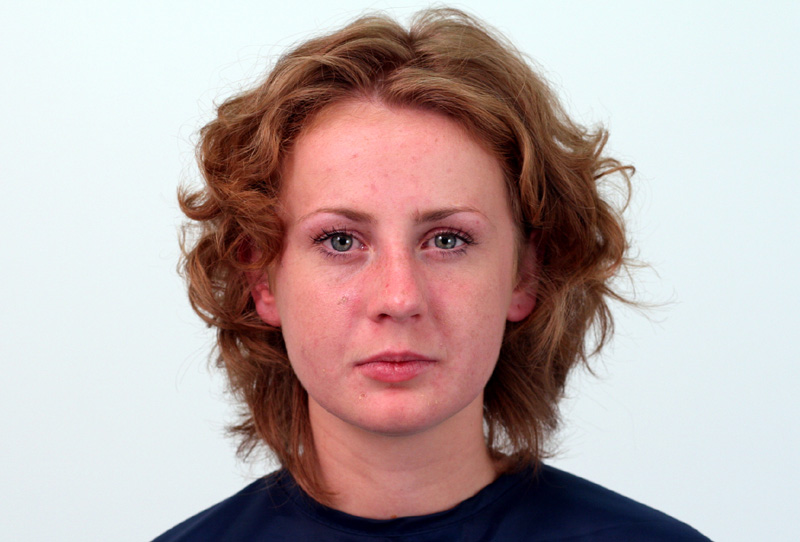

Supplement: Data Sheet 2 — WSEFEP - complete pictures dataset. [file DataSheet2.ZIP › PS_0216-lo.jpg]

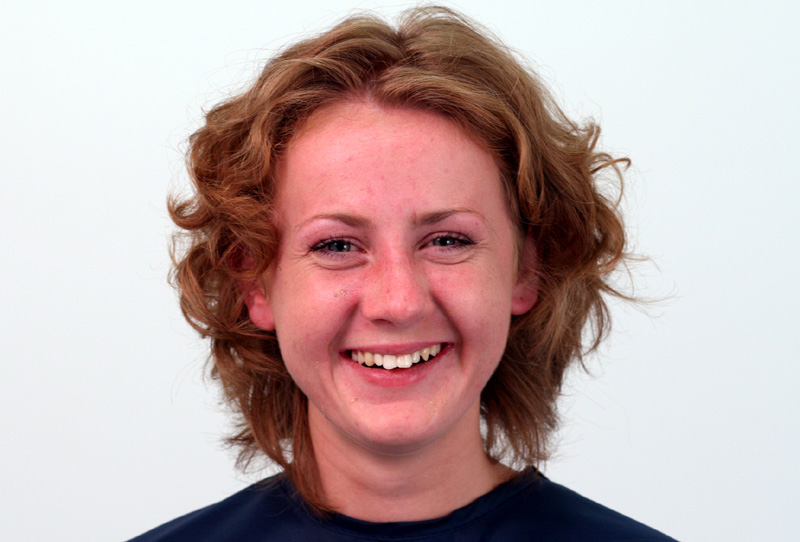

Supplement: Data Sheet 2 — WSEFEP - complete pictures dataset. [file DataSheet2.ZIP › PS_0236-lo.jpg]

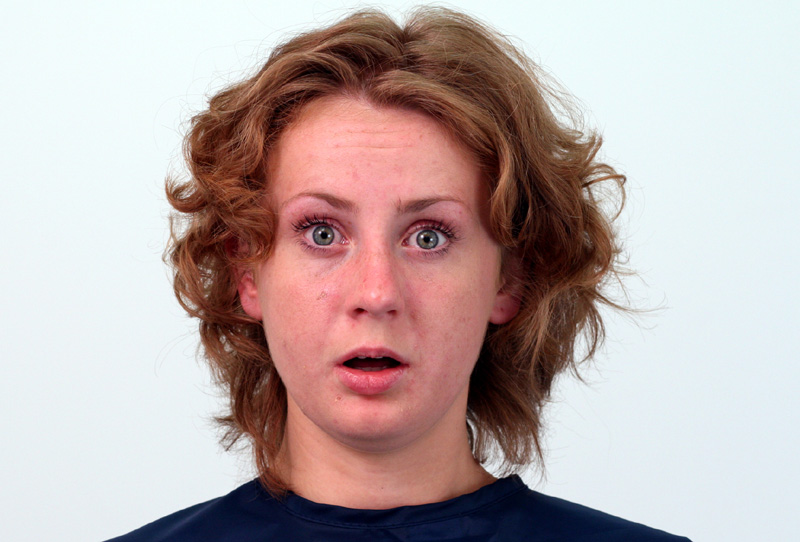

Supplement: Data Sheet 2 — WSEFEP - complete pictures dataset. [file DataSheet2.ZIP › PS_0282-lo.jpg]

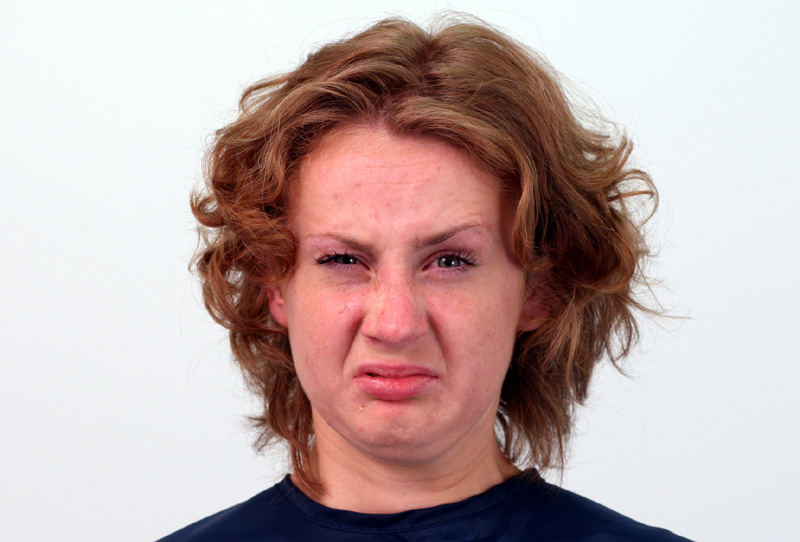

Supplement: Data Sheet 2 — WSEFEP - complete pictures dataset. [file DataSheet2.ZIP › PS_0719-lo.jpg]

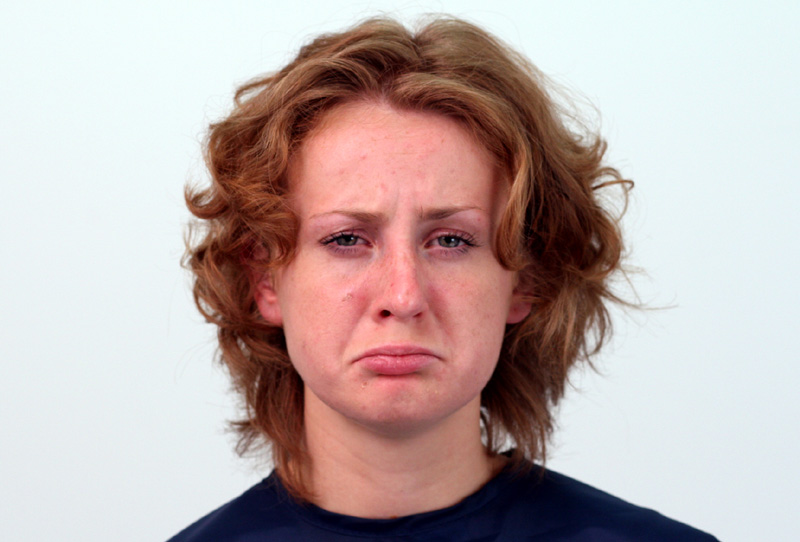

Supplement: Data Sheet 2 — WSEFEP - complete pictures dataset. [file DataSheet2.ZIP › PS_0746-lo.jpg]

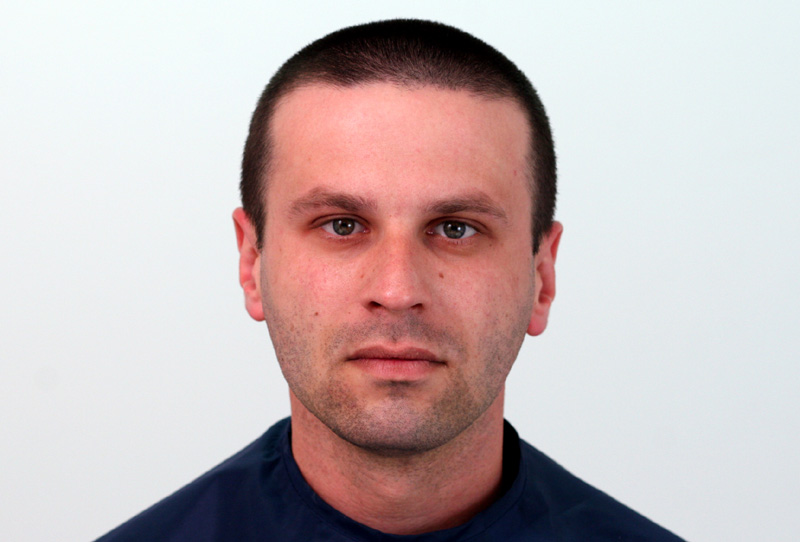

Supplement: Data Sheet 2 — WSEFEP - complete pictures dataset. [file DataSheet2.ZIP › RA_0057-lo.jpg]

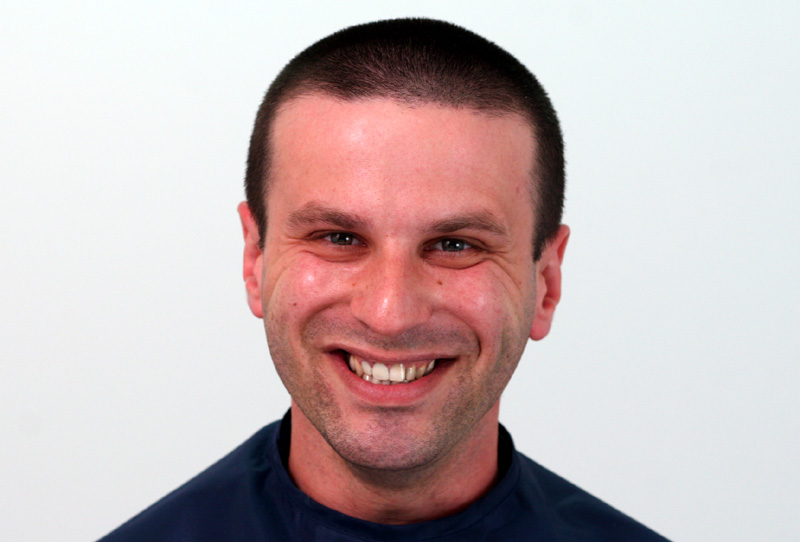

Supplement: Data Sheet 2 — WSEFEP - complete pictures dataset. [file DataSheet2.ZIP › RA_0317-lo.jpg]

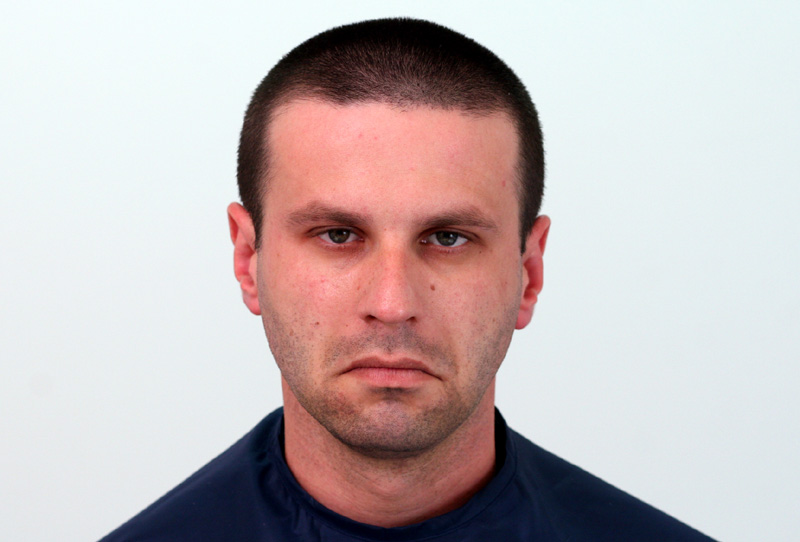

Supplement: Data Sheet 2 — WSEFEP - complete pictures dataset. [file DataSheet2.ZIP › RA_1215-lo.jpg]

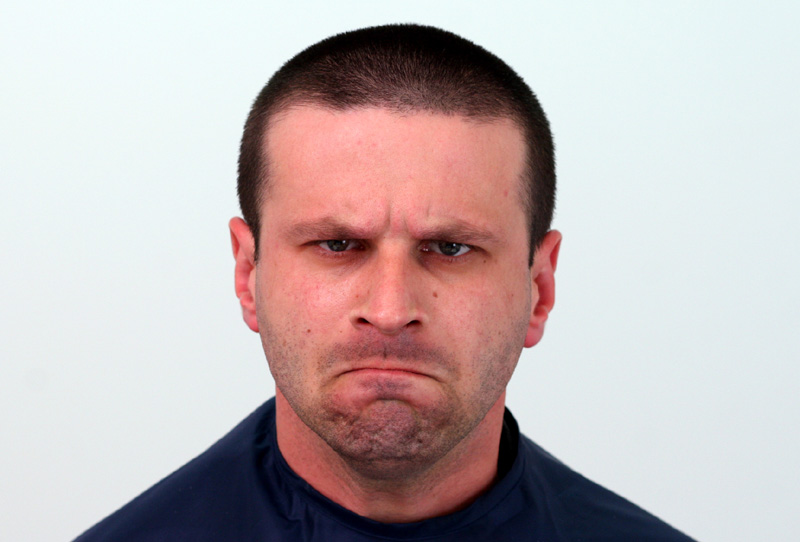

Supplement: Data Sheet 2 — WSEFEP - complete pictures dataset. [file DataSheet2.ZIP › RA_1786-lo.jpg]

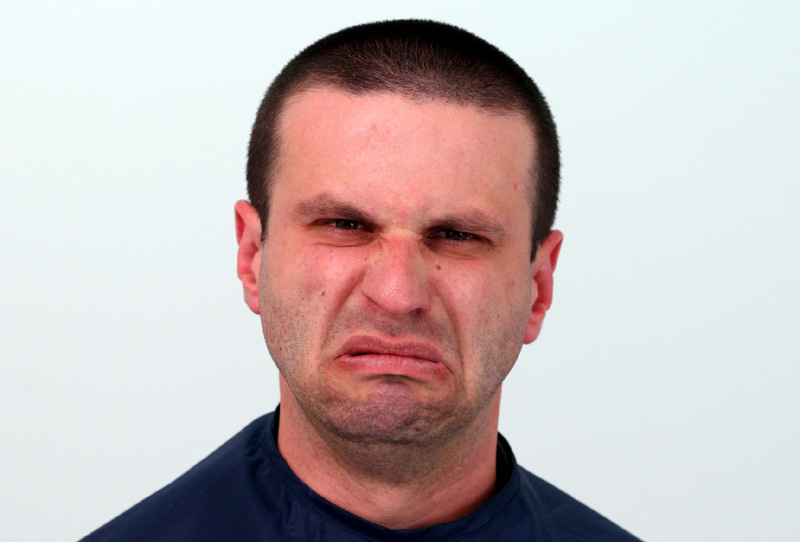

Supplement: Data Sheet 2 — WSEFEP - complete pictures dataset. [file DataSheet2.ZIP › RA_2267-lo.jpg]

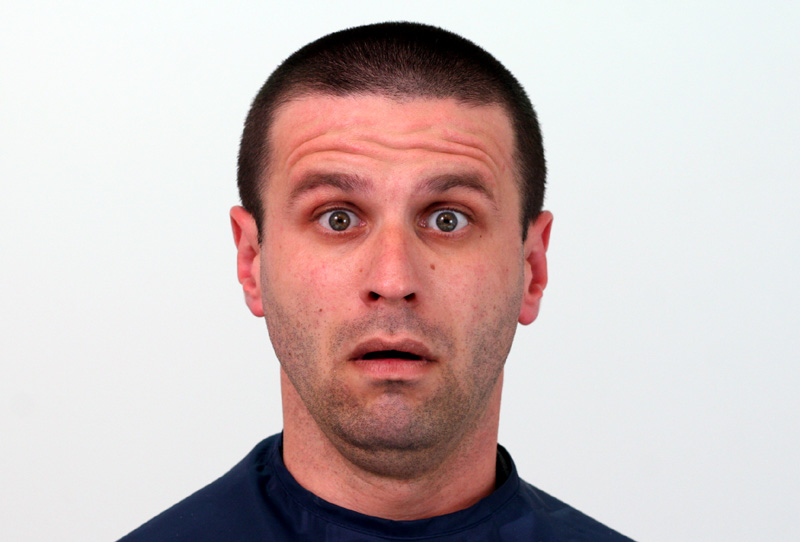

Supplement: Data Sheet 2 — WSEFEP - complete pictures dataset. [file DataSheet2.ZIP › RA_2800-lo.jpg]

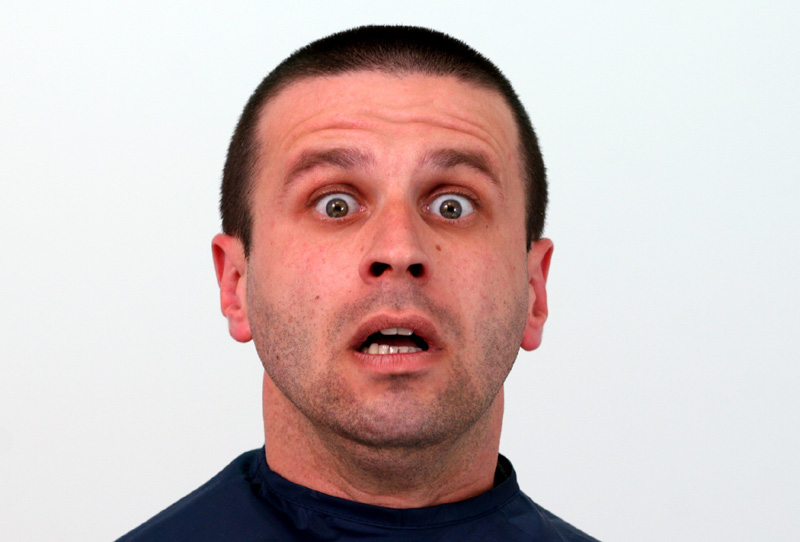

Supplement: Data Sheet 2 — WSEFEP - complete pictures dataset. [file DataSheet2.ZIP › RA_3483-lo.jpg]

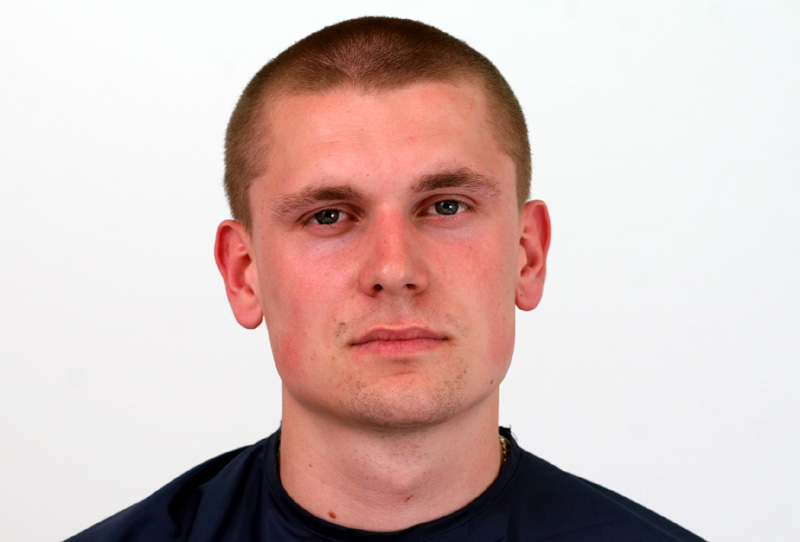

Supplement: Data Sheet 2 — WSEFEP - complete pictures dataset. [file DataSheet2.ZIP › RB_0006-lo.jpg]

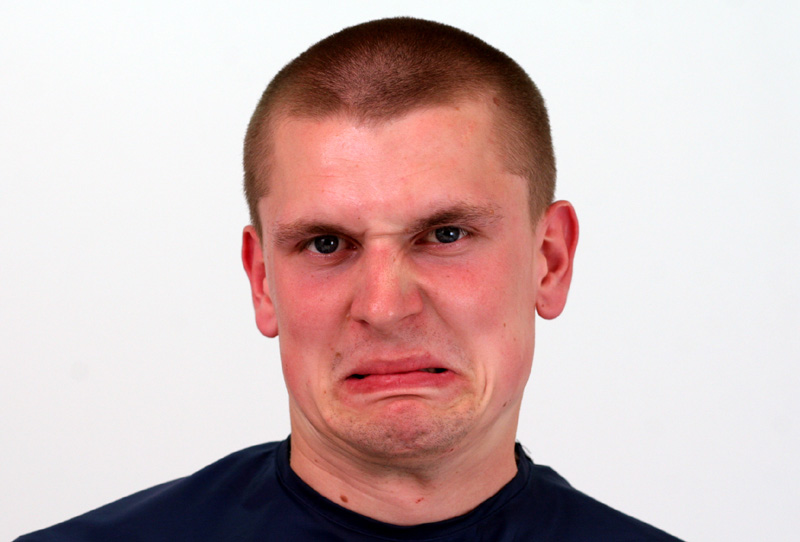

Supplement: Data Sheet 2 — WSEFEP - complete pictures dataset. [file DataSheet2.ZIP › RB_0167-lo.jpg]

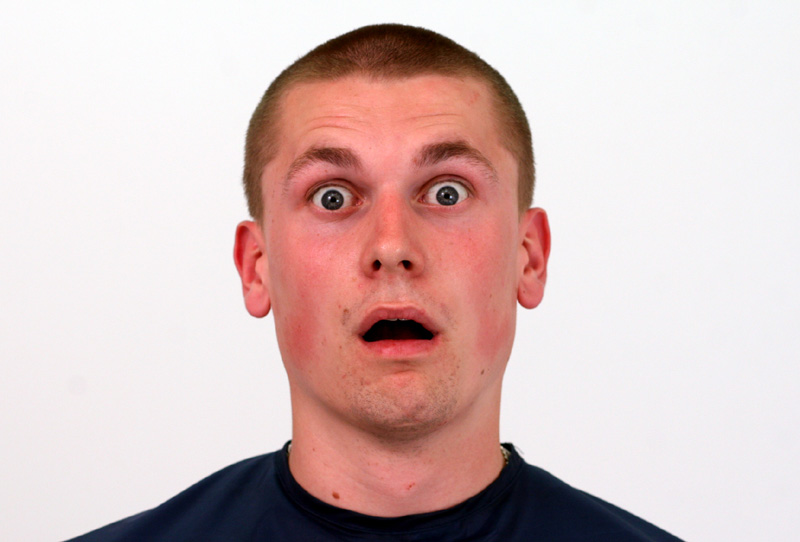

Supplement: Data Sheet 2 — WSEFEP - complete pictures dataset. [file DataSheet2.ZIP › RB_0255-lo.jpg]

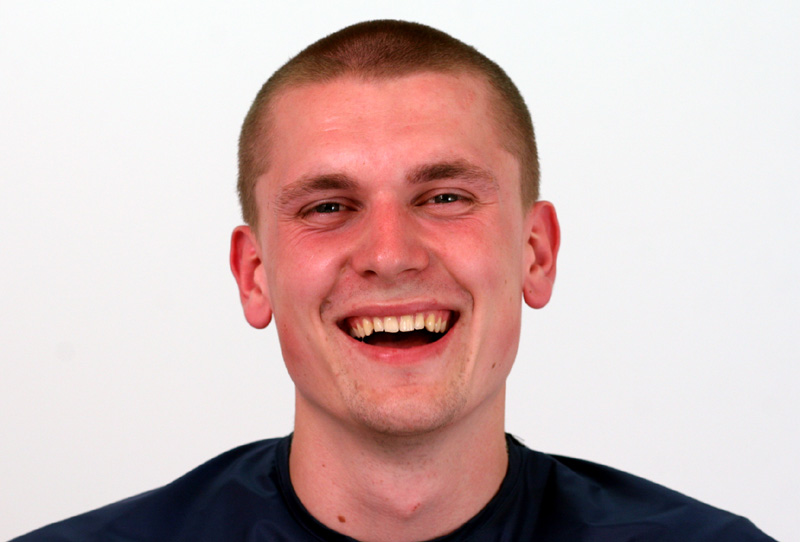

Supplement: Data Sheet 2 — WSEFEP - complete pictures dataset. [file DataSheet2.ZIP › RB_0329-lo.jpg]

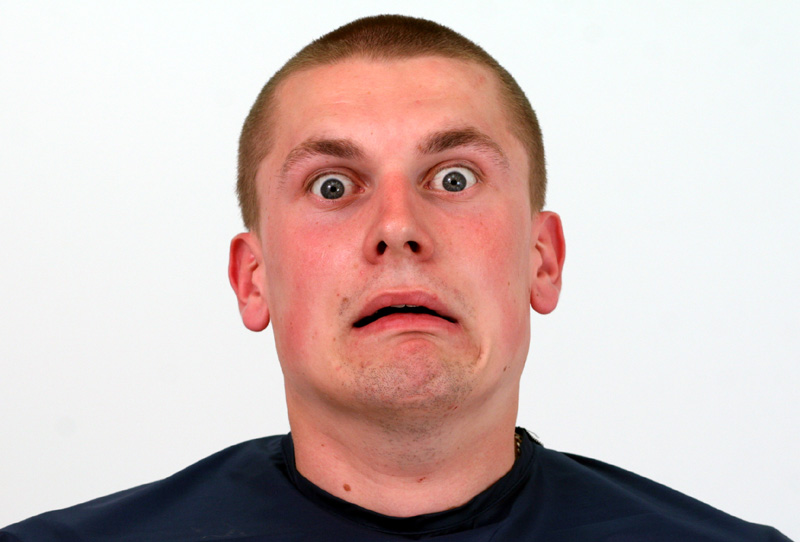

Supplement: Data Sheet 2 — WSEFEP - complete pictures dataset. [file DataSheet2.ZIP › RB_0392-lo.jpg]

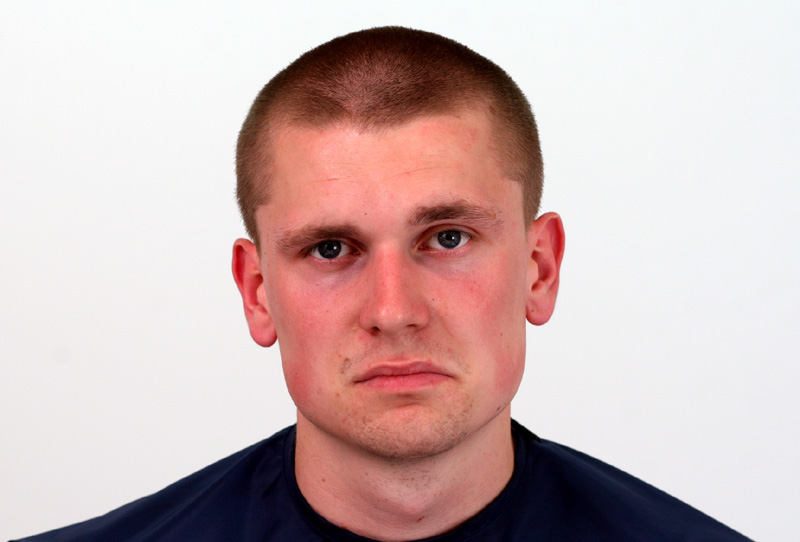

Supplement: Data Sheet 2 — WSEFEP - complete pictures dataset. [file DataSheet2.ZIP › RB_0458-lo.jpg]

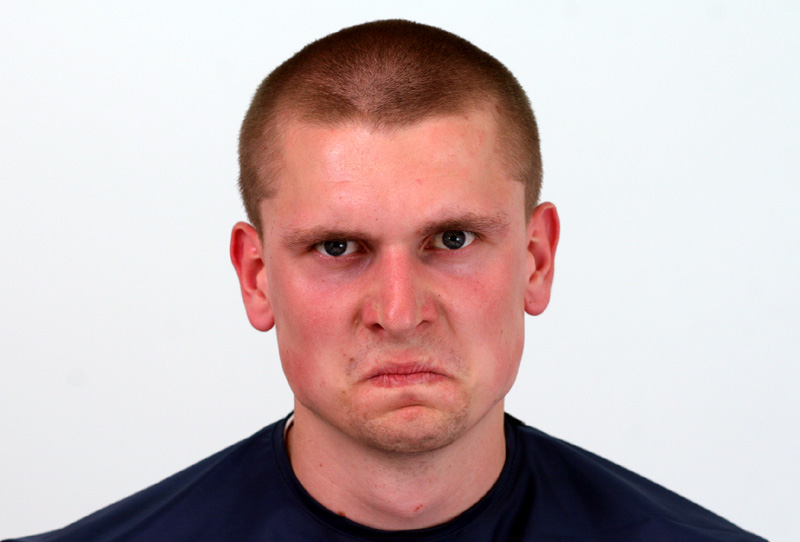

Supplement: Data Sheet 2 — WSEFEP - complete pictures dataset. [file DataSheet2.ZIP › RB_0586-lo.jpg]

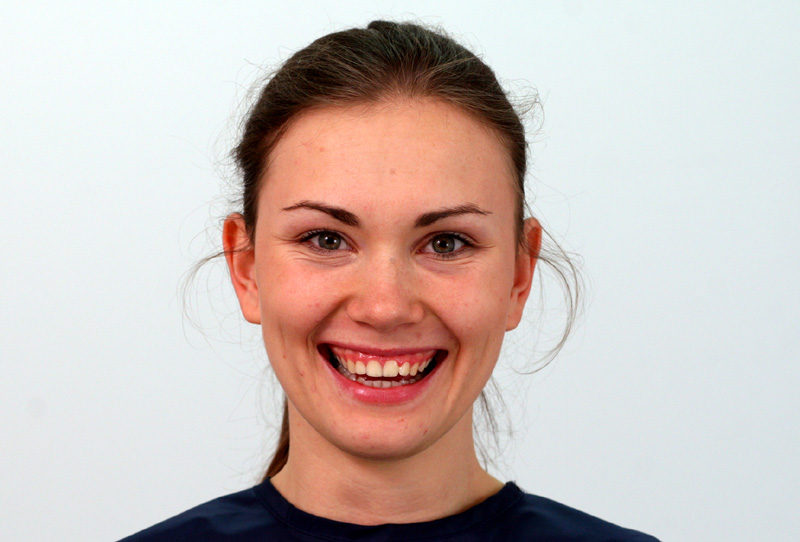

Supplement: Data Sheet 2 — WSEFEP - complete pictures dataset. [file DataSheet2.ZIP › SO_0028-lo.jpg]

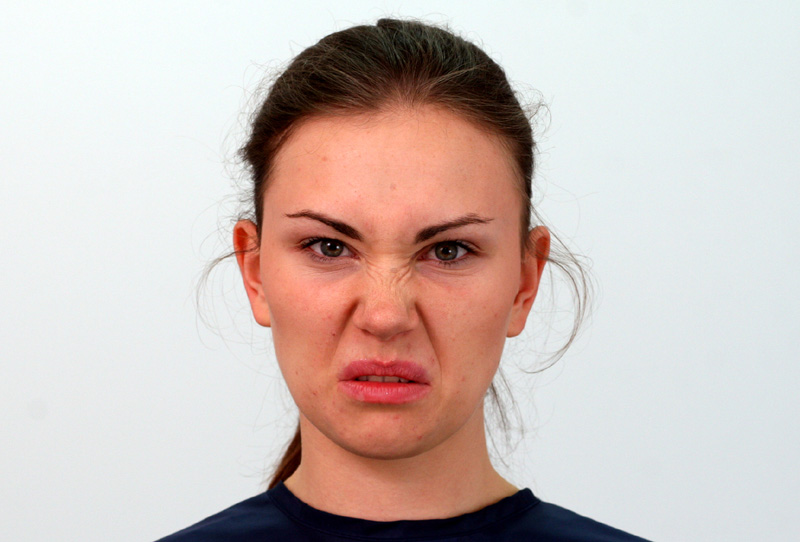

Supplement: Data Sheet 2 — WSEFEP - complete pictures dataset. [file DataSheet2.ZIP › SO_0053-lo.jpg]

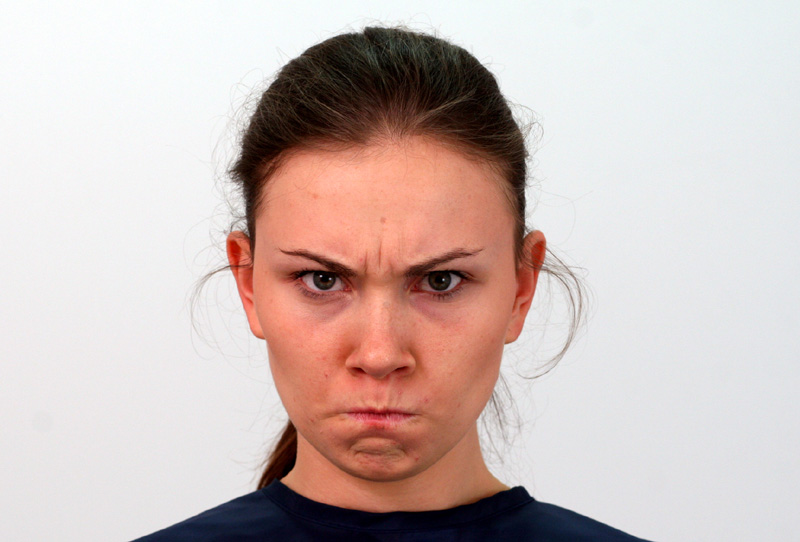

Supplement: Data Sheet 2 — WSEFEP - complete pictures dataset. [file DataSheet2.ZIP › SO_0071-lo.jpg]

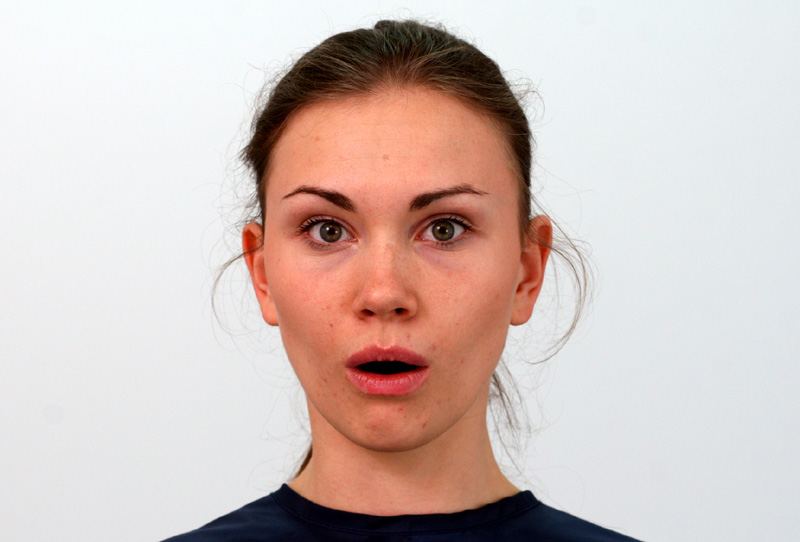

Supplement: Data Sheet 2 — WSEFEP - complete pictures dataset. [file DataSheet2.ZIP › SO_0223-lo.jpg]

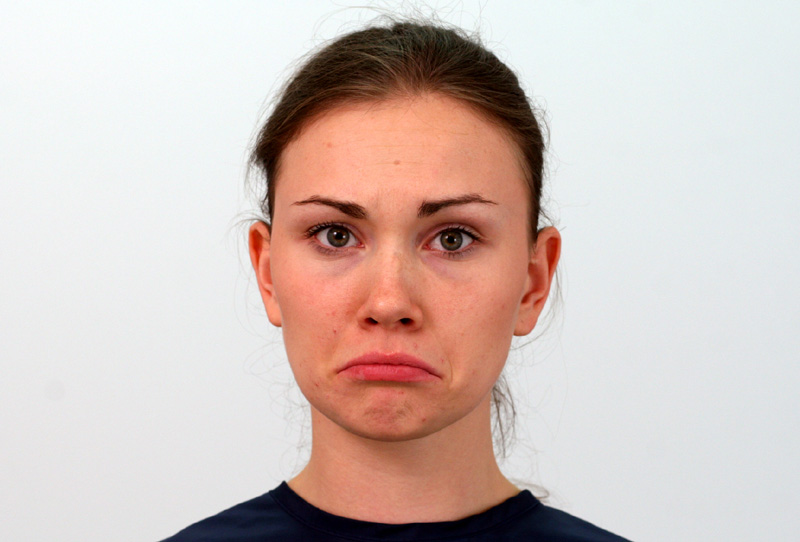

Supplement: Data Sheet 2 — WSEFEP - complete pictures dataset. [file DataSheet2.ZIP › SO_0893-lo.jpg]

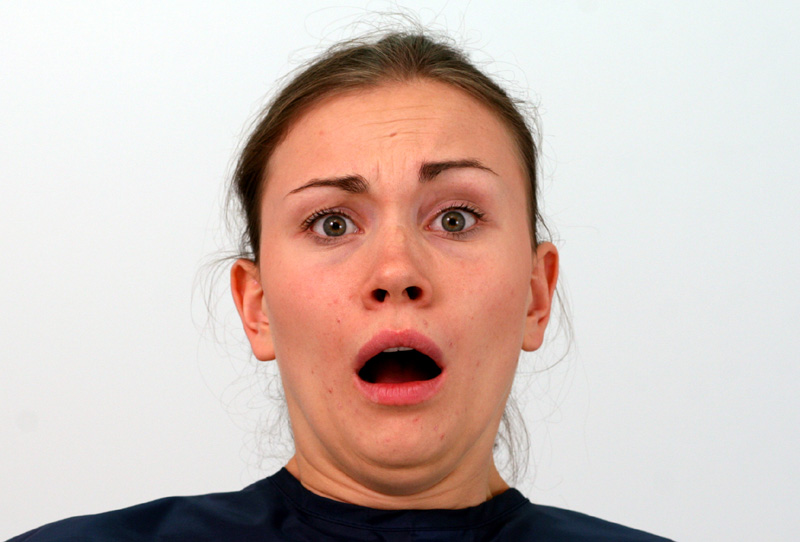

Supplement: Data Sheet 2 — WSEFEP - complete pictures dataset. [file DataSheet2.ZIP › SO_1515-lo.jpg]

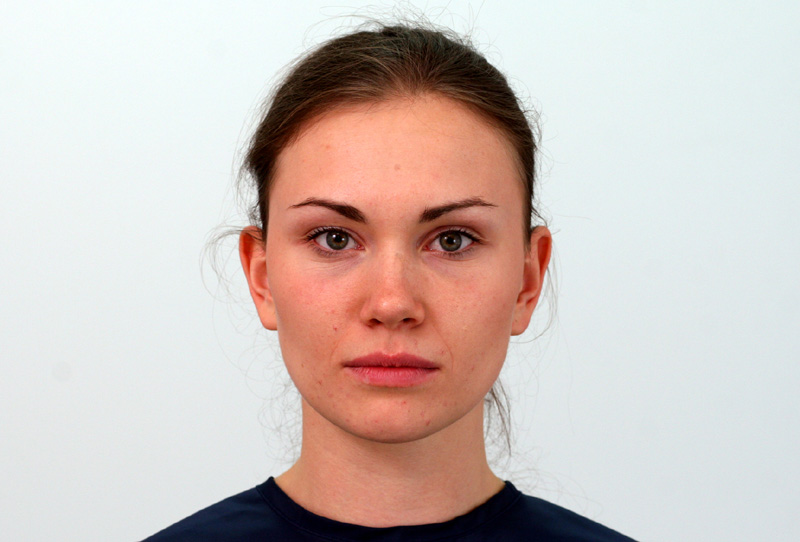

Supplement: Data Sheet 2 — WSEFEP - complete pictures dataset. [file DataSheet2.ZIP › SO_2188-lo.jpg]

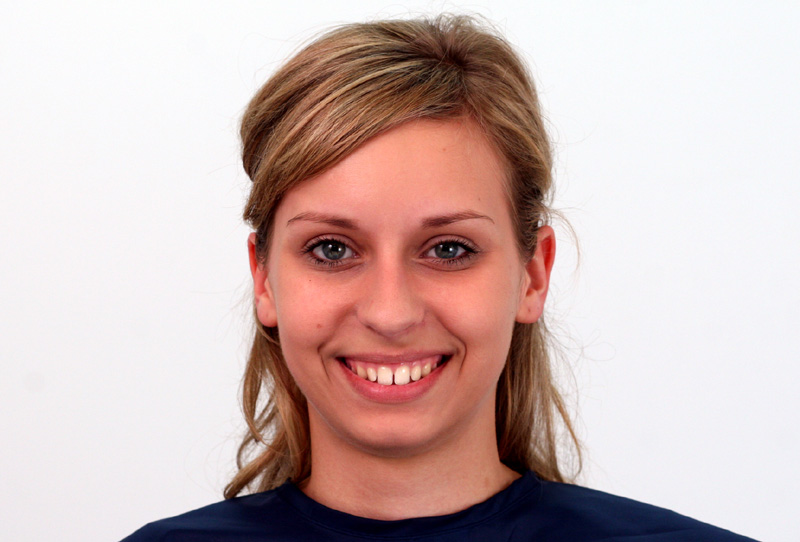

Supplement: Data Sheet 2 — WSEFEP - complete pictures dataset. [file DataSheet2.ZIP › SS_0018-lo.jpg]

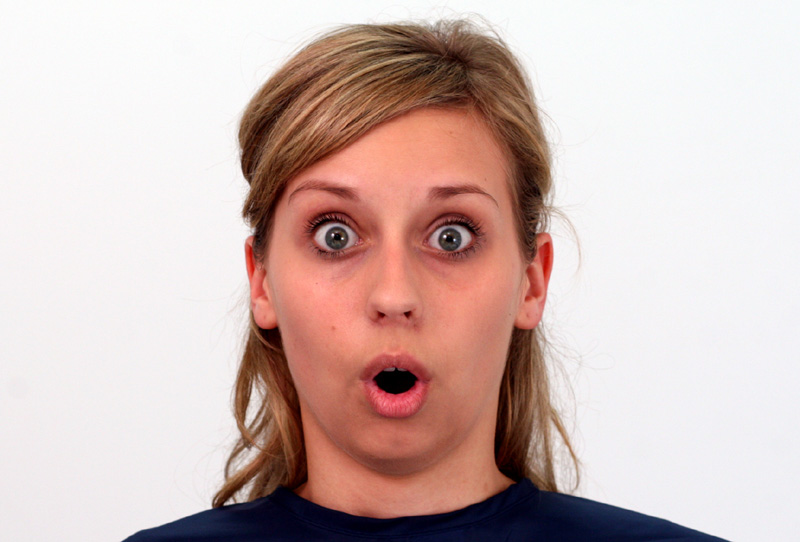

Supplement: Data Sheet 2 — WSEFEP - complete pictures dataset. [file DataSheet2.ZIP › SS_0032-lo.jpg]

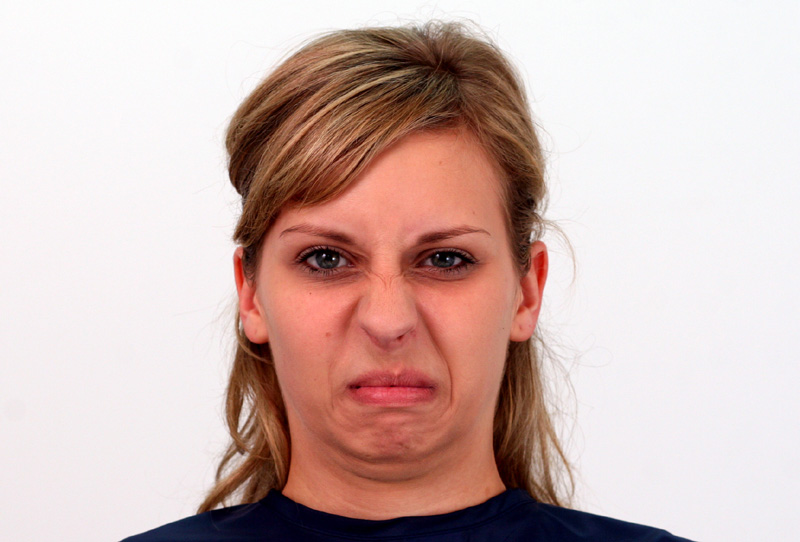

Supplement: Data Sheet 2 — WSEFEP - complete pictures dataset. [file DataSheet2.ZIP › SS_0084-lo.jpg]

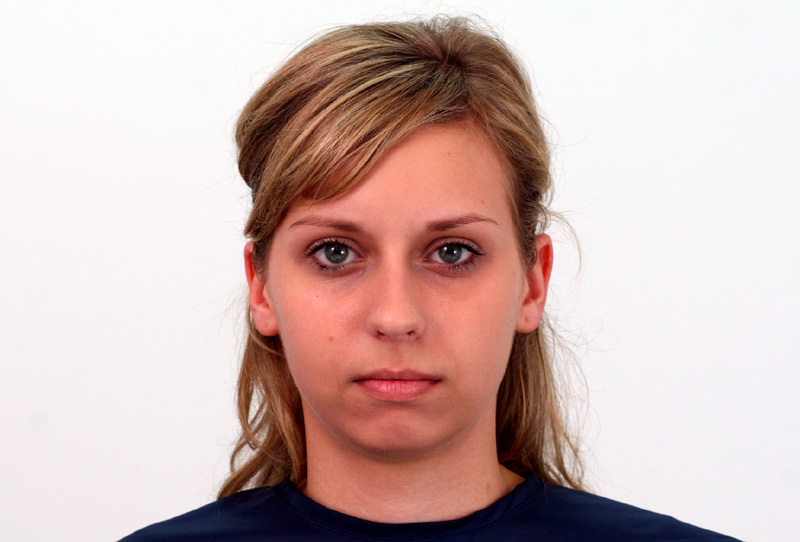

Supplement: Data Sheet 2 — WSEFEP - complete pictures dataset. [file DataSheet2.ZIP › SS_0151-lo.jpg]
