# Supplementary figures and images for: Warsaw set of emotional facial expression pictures: a validation study of facial display photographs (part 1 of 2)
Source: Front Psychol. 2015 Jan 5;5:1516. doi: 10.3389/fpsyg.2014.01516 (PMC4283518; doi:10.3389/fpsyg.2014.01516)

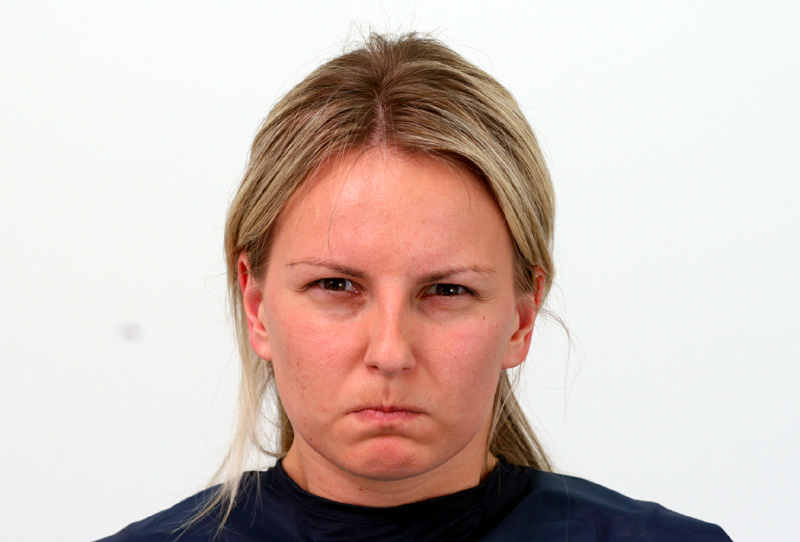

Supplement: Data Sheet 2 — WSEFEP - complete pictures dataset. [file DataSheet2.ZIP › AD_9681-lo.jpg]

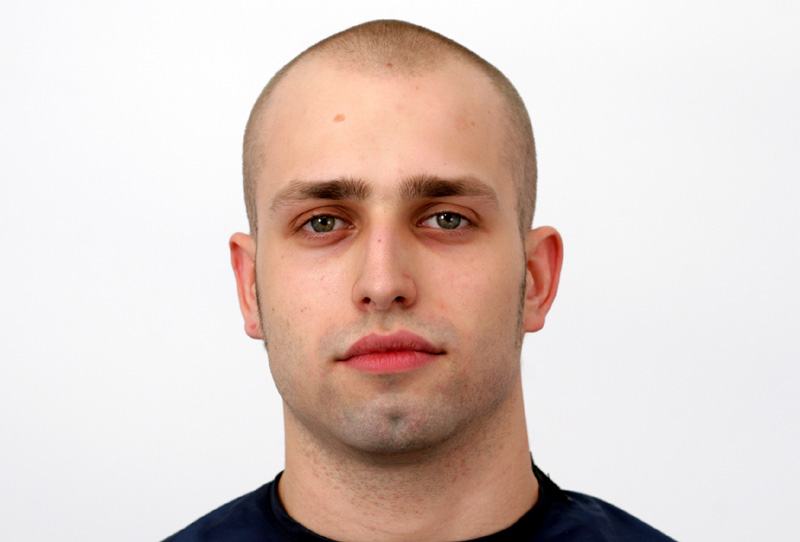

Supplement: Data Sheet 2 — WSEFEP - complete pictures dataset. [file DataSheet2.ZIP › AG_0011-lo.jpg]

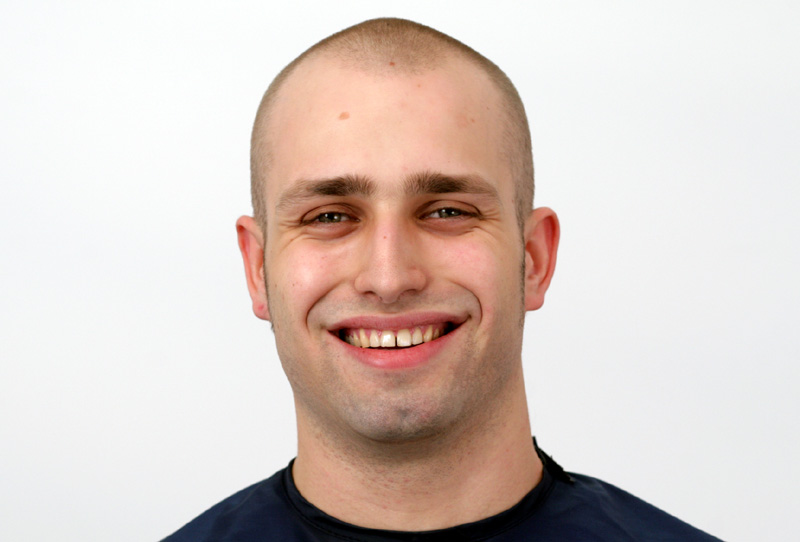

Supplement: Data Sheet 2 — WSEFEP - complete pictures dataset. [file DataSheet2.ZIP › AG_0086-lo.jpg]

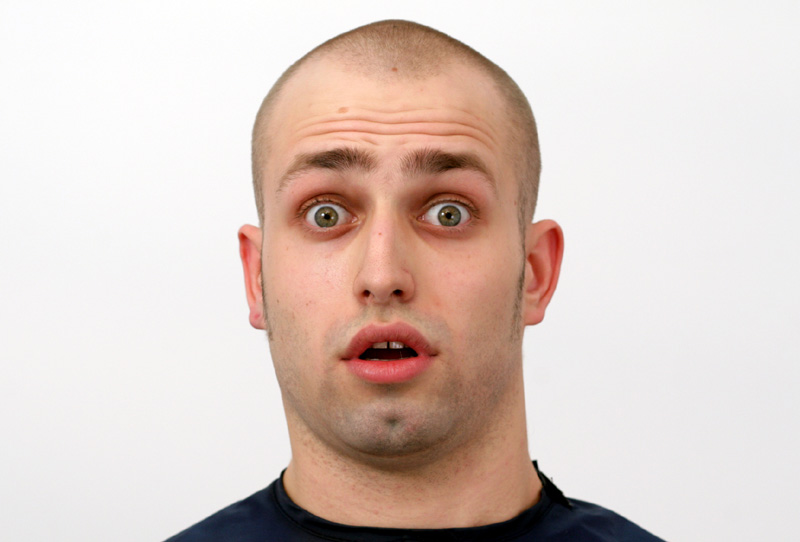

Supplement: Data Sheet 2 — WSEFEP - complete pictures dataset. [file DataSheet2.ZIP › AG_0283-lo.jpg]

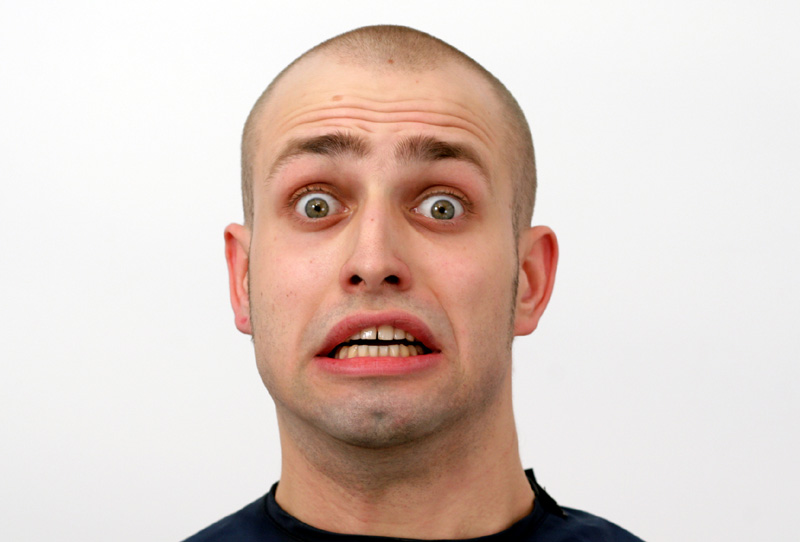

Supplement: Data Sheet 2 — WSEFEP - complete pictures dataset. [file DataSheet2.ZIP › AG_0424-lo.jpg]

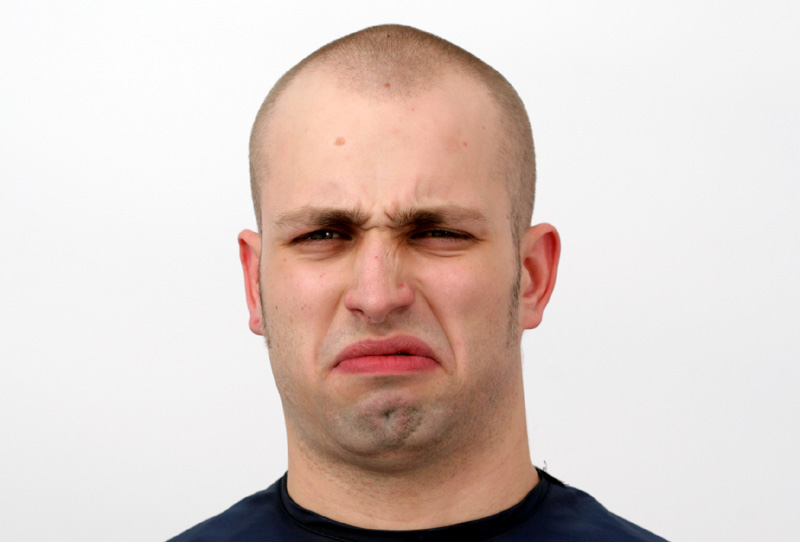

Supplement: Data Sheet 2 — WSEFEP - complete pictures dataset. [file DataSheet2.ZIP › AG_0666-lo.jpg]

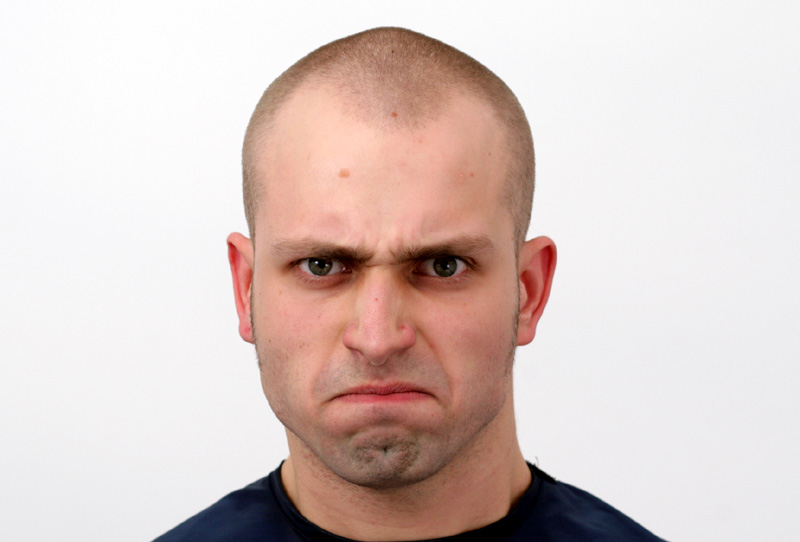

Supplement: Data Sheet 2 — WSEFEP - complete pictures dataset. [file DataSheet2.ZIP › AG_1314-lo.jpg]

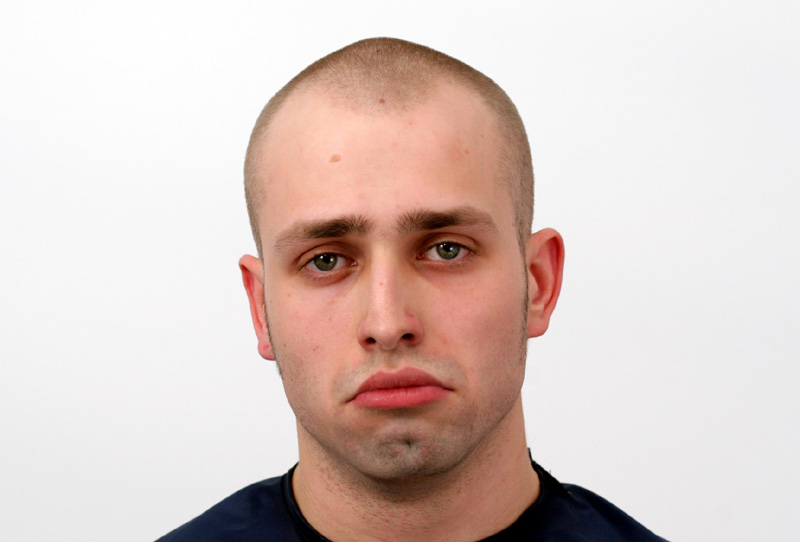

Supplement: Data Sheet 2 — WSEFEP - complete pictures dataset. [file DataSheet2.ZIP › AG_1460-lo.jpg]

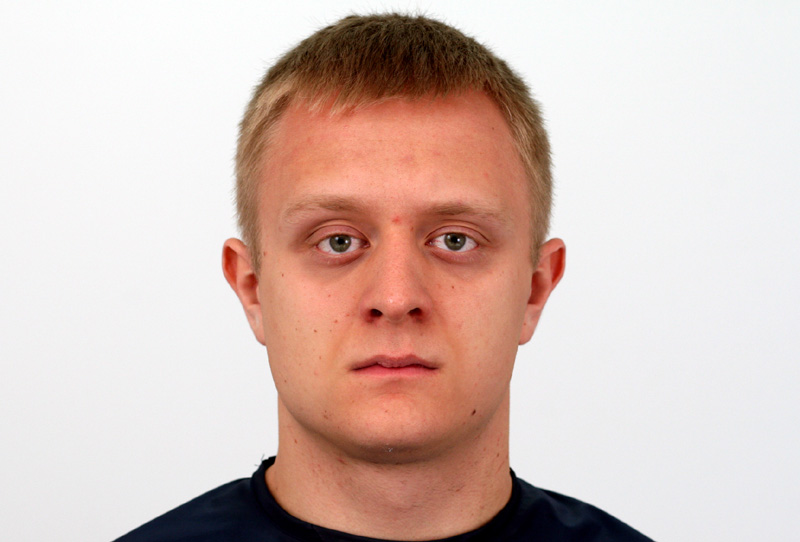

Supplement: Data Sheet 2 — WSEFEP - complete pictures dataset. [file DataSheet2.ZIP › DC_0014-lo.jpg]

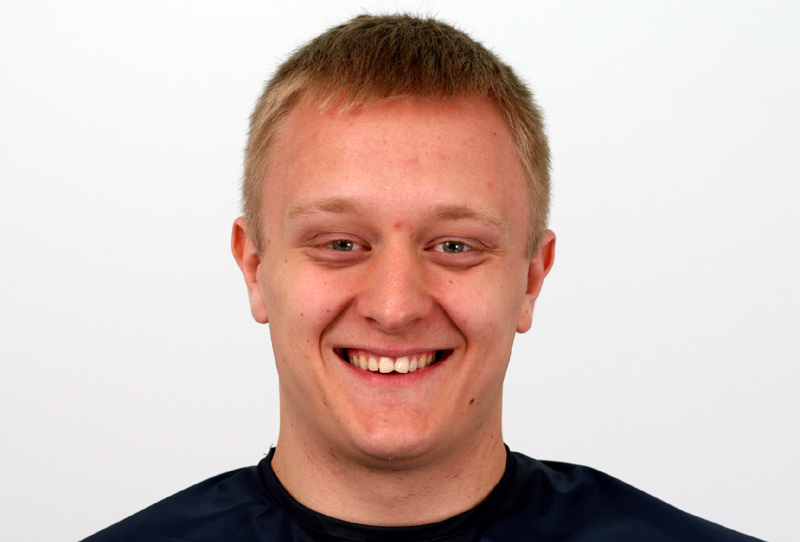

Supplement: Data Sheet 2 — WSEFEP - complete pictures dataset. [file DataSheet2.ZIP › DC_0139-lo.jpg]

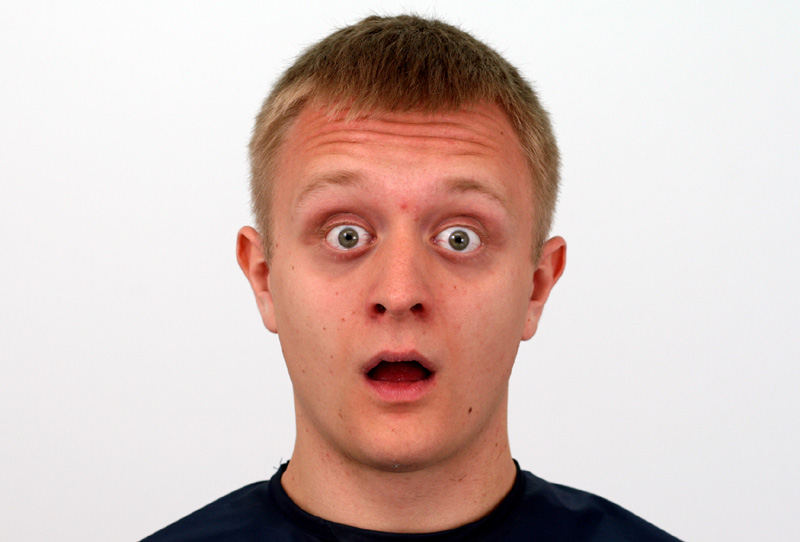

Supplement: Data Sheet 2 — WSEFEP - complete pictures dataset. [file DataSheet2.ZIP › DC_0272-lo.jpg]

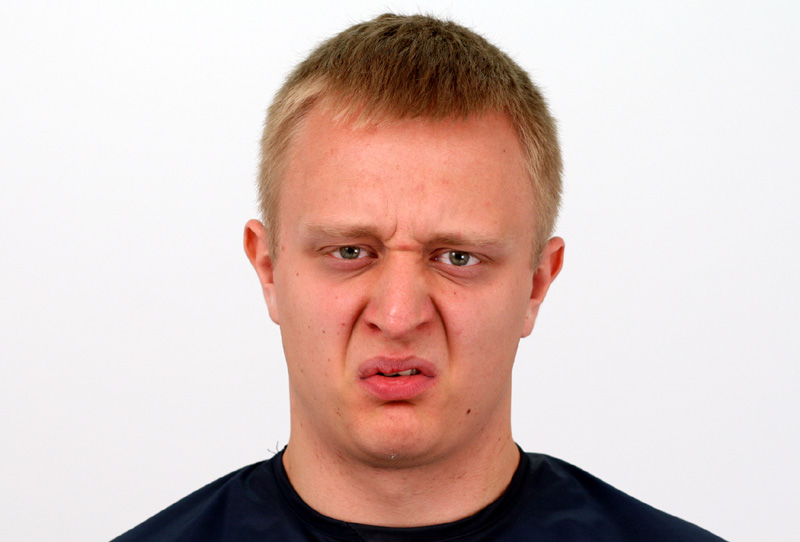

Supplement: Data Sheet 2 — WSEFEP - complete pictures dataset. [file DataSheet2.ZIP › DC_0616-lo.jpg]

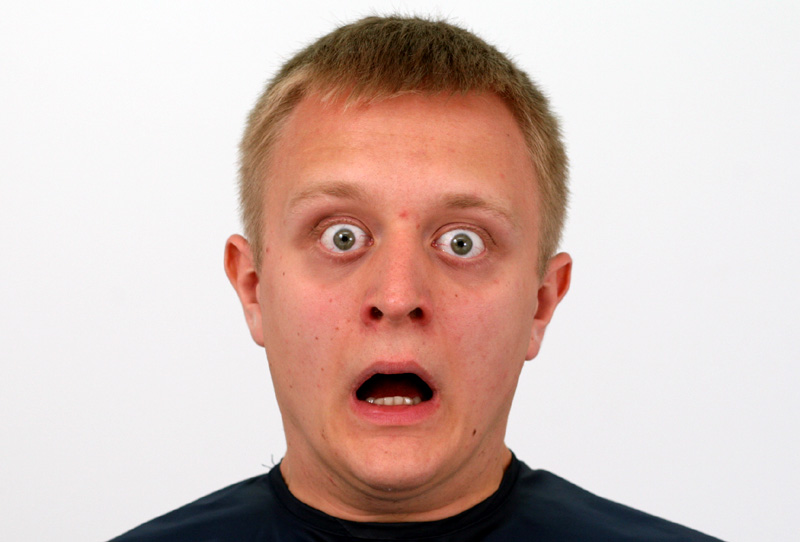

Supplement: Data Sheet 2 — WSEFEP - complete pictures dataset. [file DataSheet2.ZIP › DC_0952-lo.jpg]

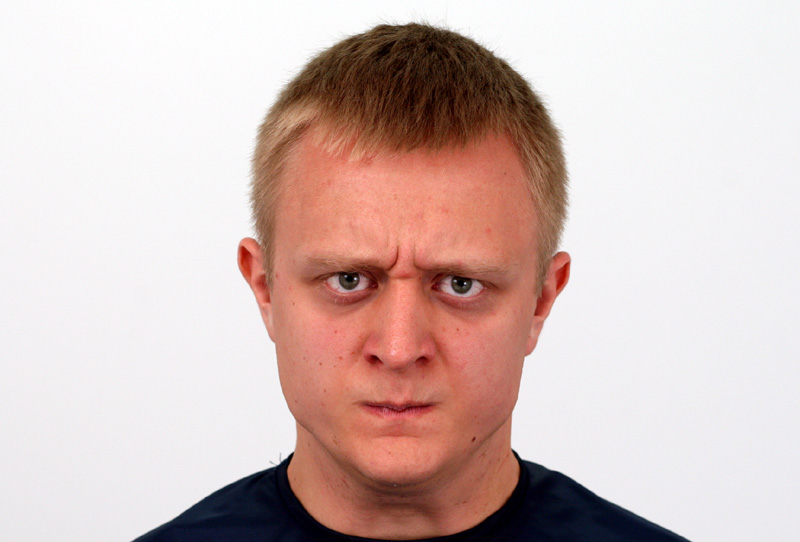

Supplement: Data Sheet 2 — WSEFEP - complete pictures dataset. [file DataSheet2.ZIP › DC_1317-lo.jpg]

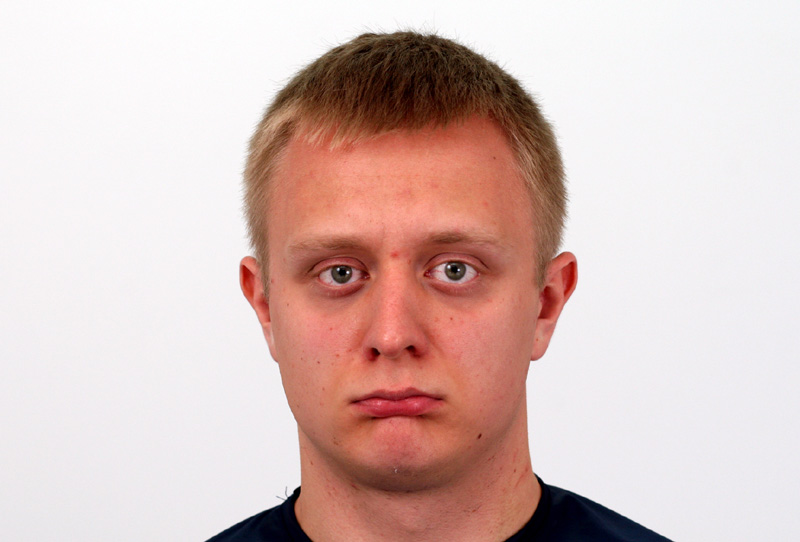

Supplement: Data Sheet 2 — WSEFEP - complete pictures dataset. [file DataSheet2.ZIP › DC_1399-lo.jpg]

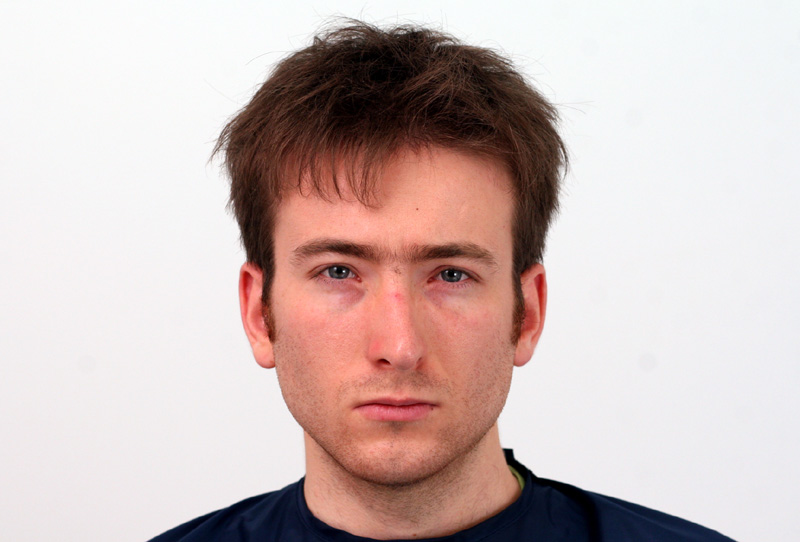

Supplement: Data Sheet 2 — WSEFEP - complete pictures dataset. [file DataSheet2.ZIP › HW_0006-lo.jpg]

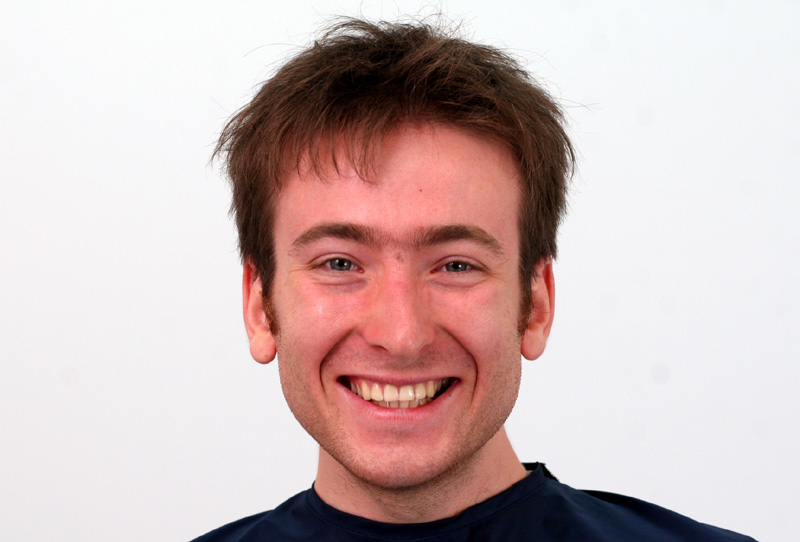

Supplement: Data Sheet 2 — WSEFEP - complete pictures dataset. [file DataSheet2.ZIP › HW_0068-lo.jpg]

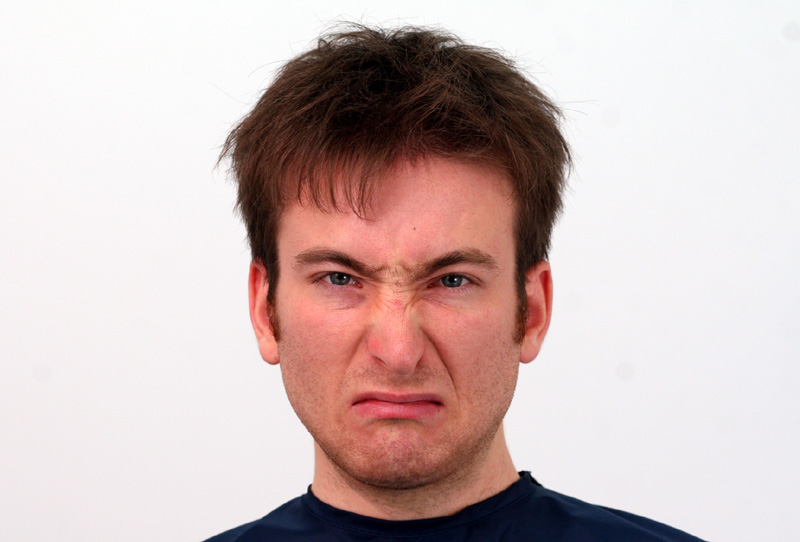

Supplement: Data Sheet 2 — WSEFEP - complete pictures dataset. [file DataSheet2.ZIP › HW_0452-lo.jpg]

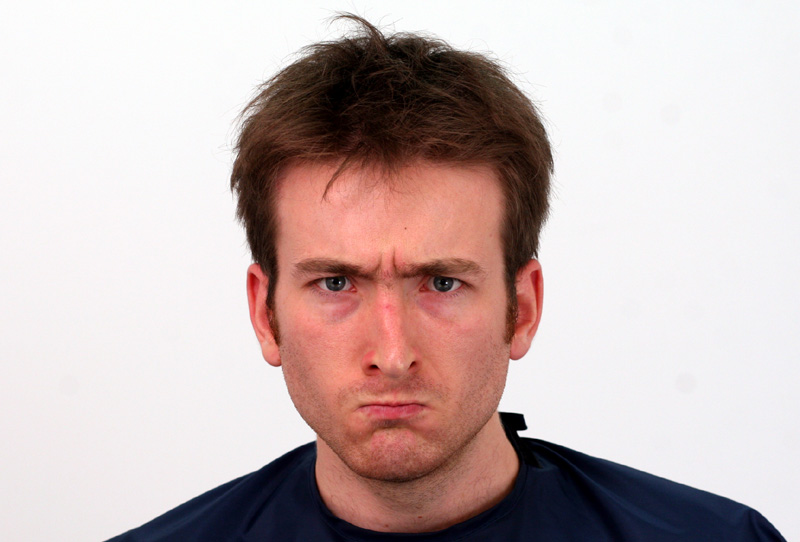

Supplement: Data Sheet 2 — WSEFEP - complete pictures dataset. [file DataSheet2.ZIP › HW_1612-lo.jpg]

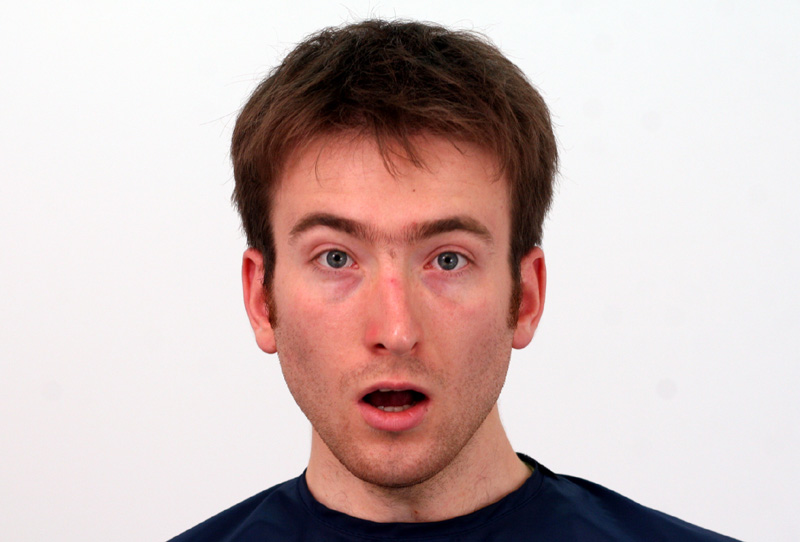

Supplement: Data Sheet 2 — WSEFEP - complete pictures dataset. [file DataSheet2.ZIP › HW_2057-lo.jpg]

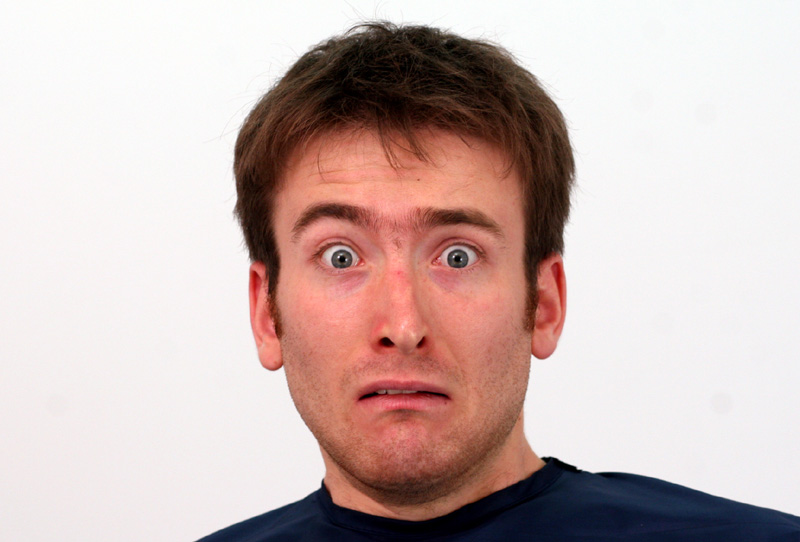

Supplement: Data Sheet 2 — WSEFEP - complete pictures dataset. [file DataSheet2.ZIP › HW_2219-lo.jpg]

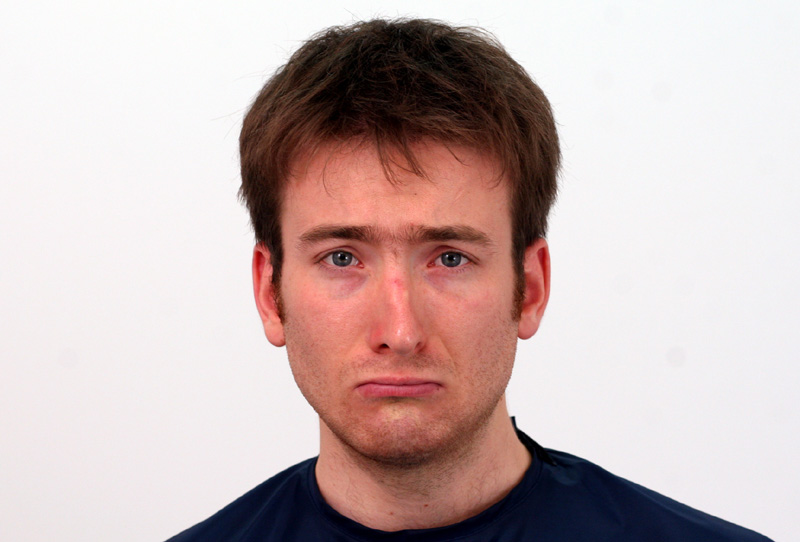

Supplement: Data Sheet 2 — WSEFEP - complete pictures dataset. [file DataSheet2.ZIP › HW_2478-lo.jpg]

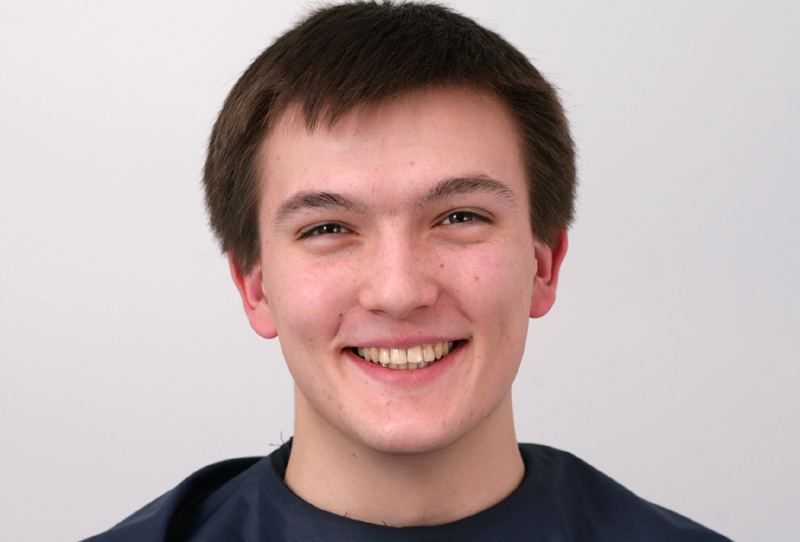

Supplement: Data Sheet 2 — WSEFEP - complete pictures dataset. [file DataSheet2.ZIP › JG_0024-lo.jpg]

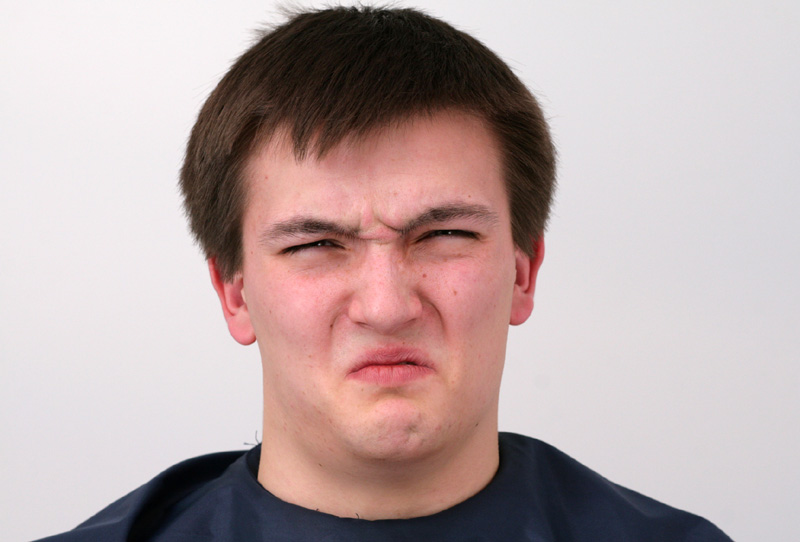

Supplement: Data Sheet 2 — WSEFEP - complete pictures dataset. [file DataSheet2.ZIP › JG_0134-lo.jpg]

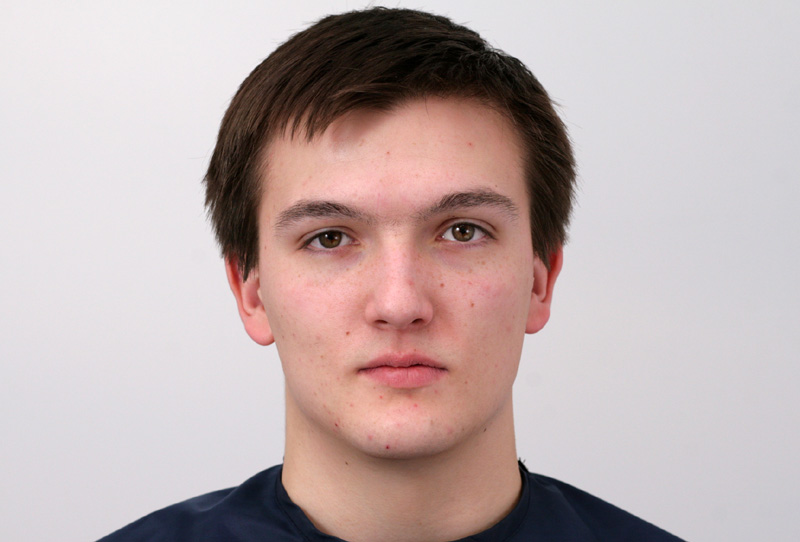

Supplement: Data Sheet 2 — WSEFEP - complete pictures dataset. [file DataSheet2.ZIP › JG_1226-lo.jpg]

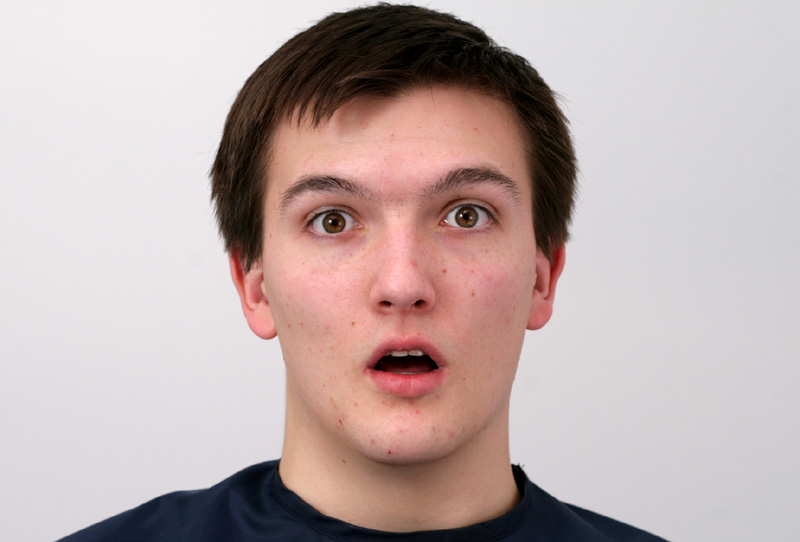

Supplement: Data Sheet 2 — WSEFEP - complete pictures dataset. [file DataSheet2.ZIP › JG_1329-lo.jpg]

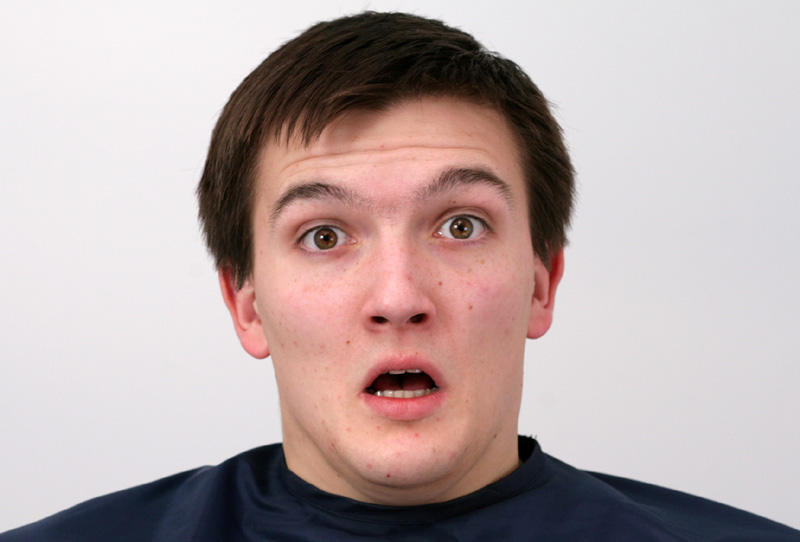

Supplement: Data Sheet 2 — WSEFEP - complete pictures dataset. [file DataSheet2.ZIP › JG_1465-lo.jpg]

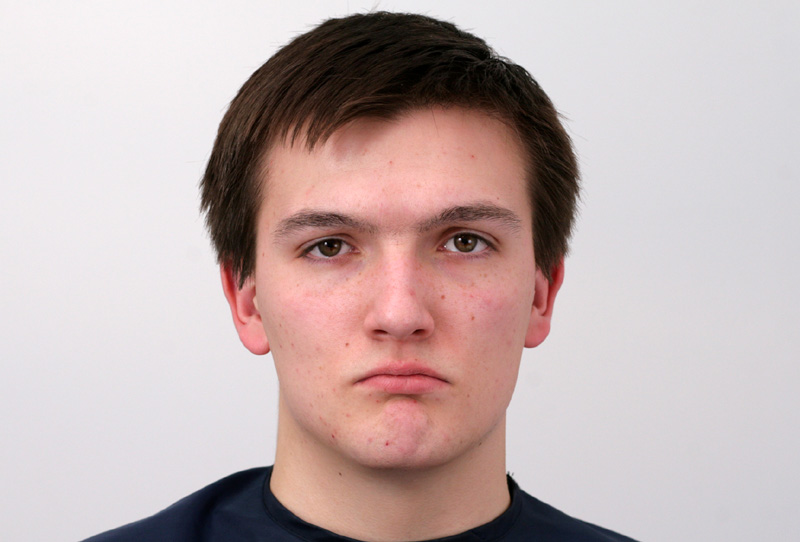

Supplement: Data Sheet 2 — WSEFEP - complete pictures dataset. [file DataSheet2.ZIP › JG_1632-lo.jpg]

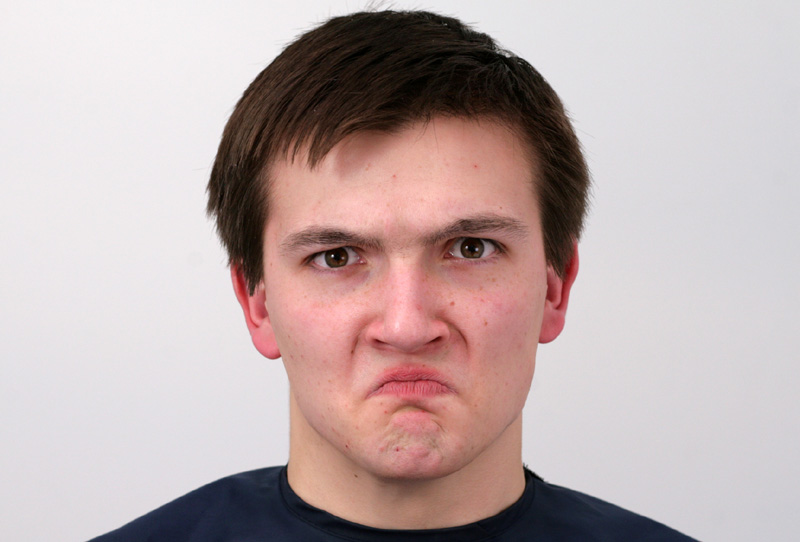

Supplement: Data Sheet 2 — WSEFEP - complete pictures dataset. [file DataSheet2.ZIP › JG_2291-lo.jpg]

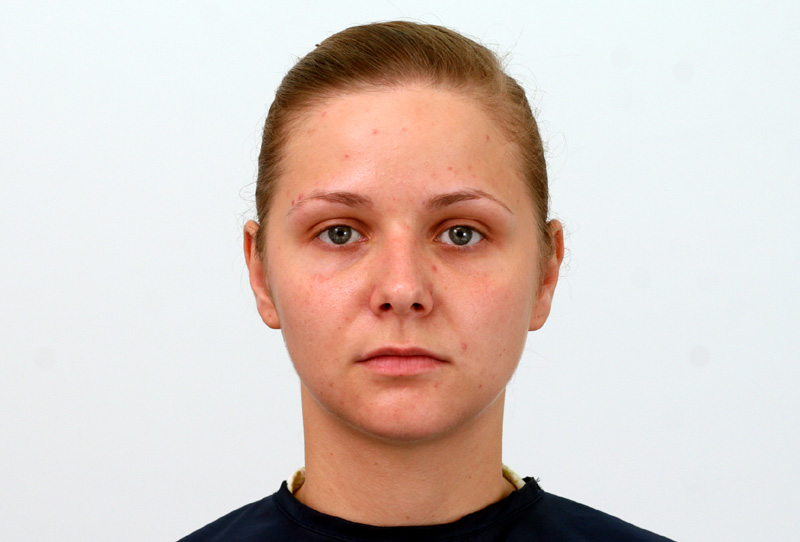

Supplement: Data Sheet 2 — WSEFEP - complete pictures dataset. [file DataSheet2.ZIP › JS_0008-lo.jpg]

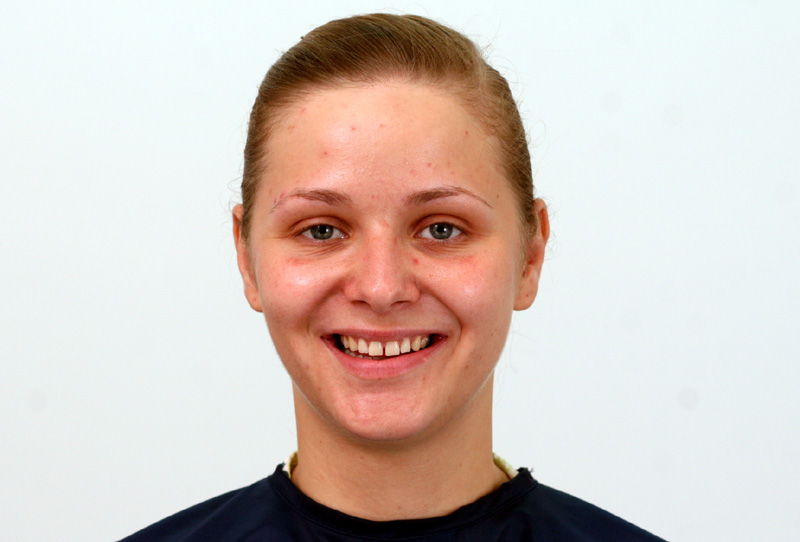

Supplement: Data Sheet 2 — WSEFEP - complete pictures dataset. [file DataSheet2.ZIP › JS_0281-lo.jpg]

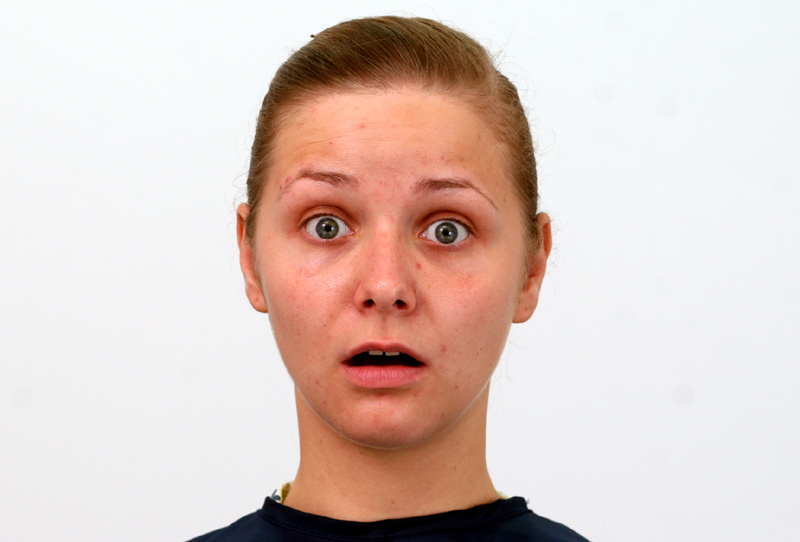

Supplement: Data Sheet 2 — WSEFEP - complete pictures dataset. [file DataSheet2.ZIP › JS_0449-lo.jpg]

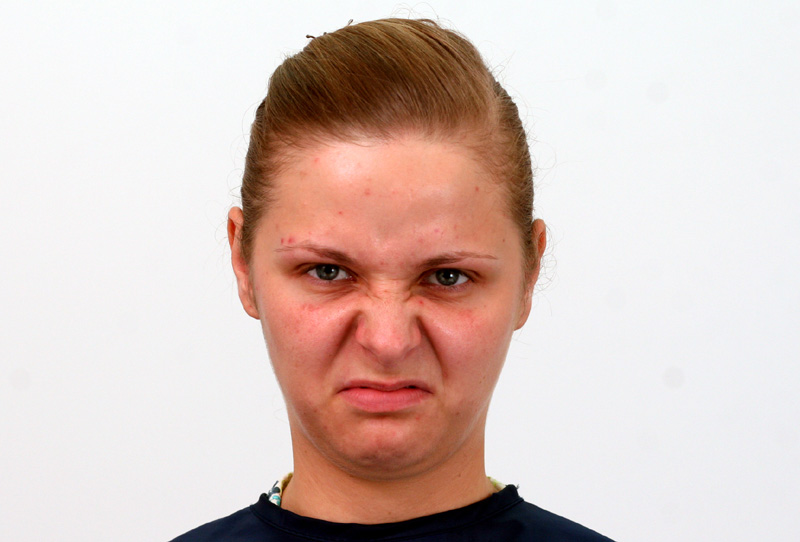

Supplement: Data Sheet 2 — WSEFEP - complete pictures dataset. [file DataSheet2.ZIP › JS_0744-lo.jpg]

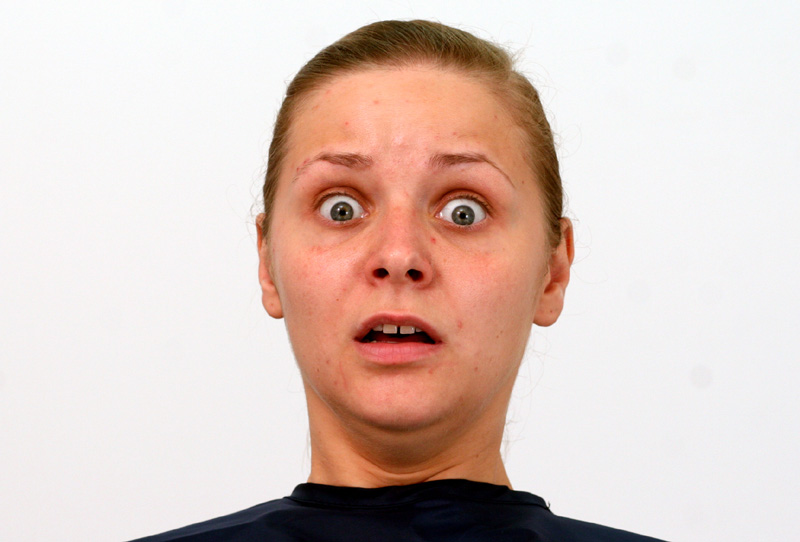

Supplement: Data Sheet 2 — WSEFEP - complete pictures dataset. [file DataSheet2.ZIP › JS_1601-lo.jpg]

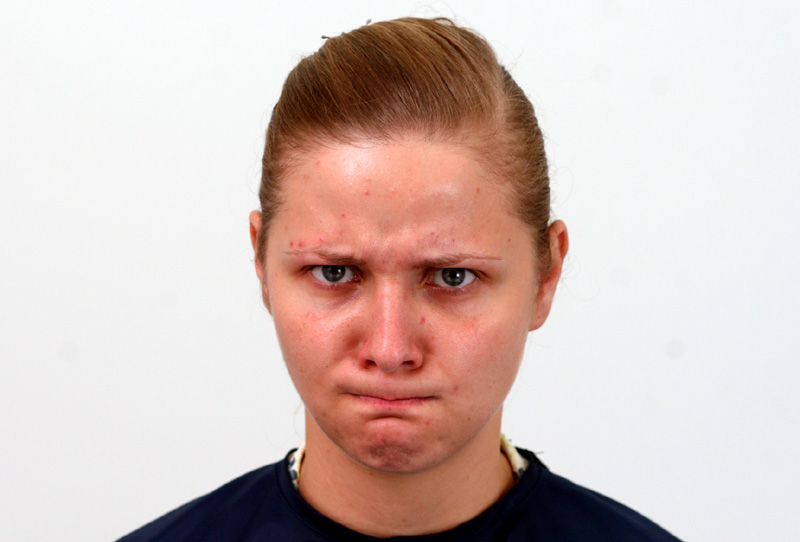

Supplement: Data Sheet 2 — WSEFEP - complete pictures dataset. [file DataSheet2.ZIP › JS_2296-lo.jpg]

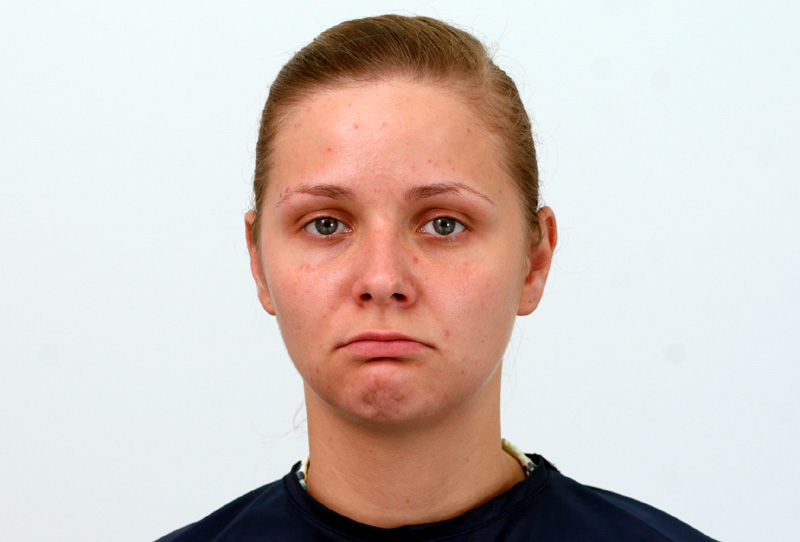

Supplement: Data Sheet 2 — WSEFEP - complete pictures dataset. [file DataSheet2.ZIP › JS_2987-lo.jpg]

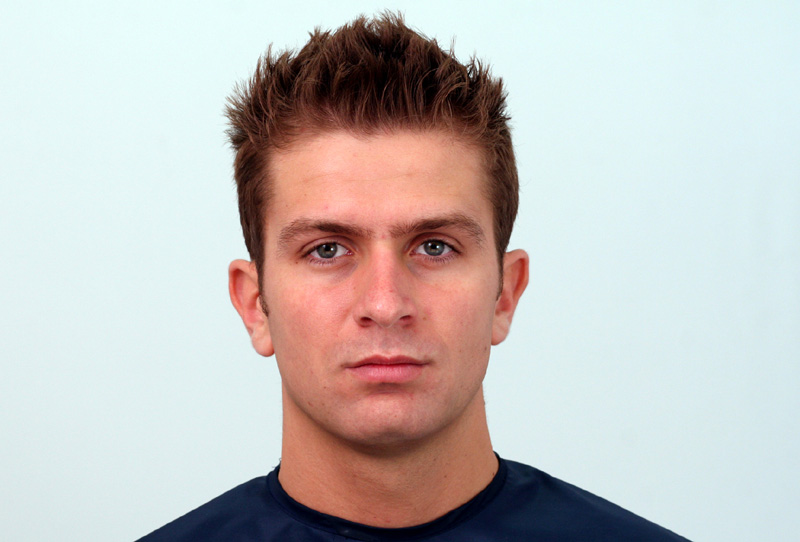

Supplement: Data Sheet 2 — WSEFEP - complete pictures dataset. [file DataSheet2.ZIP › KA_0003-lo.jpg]

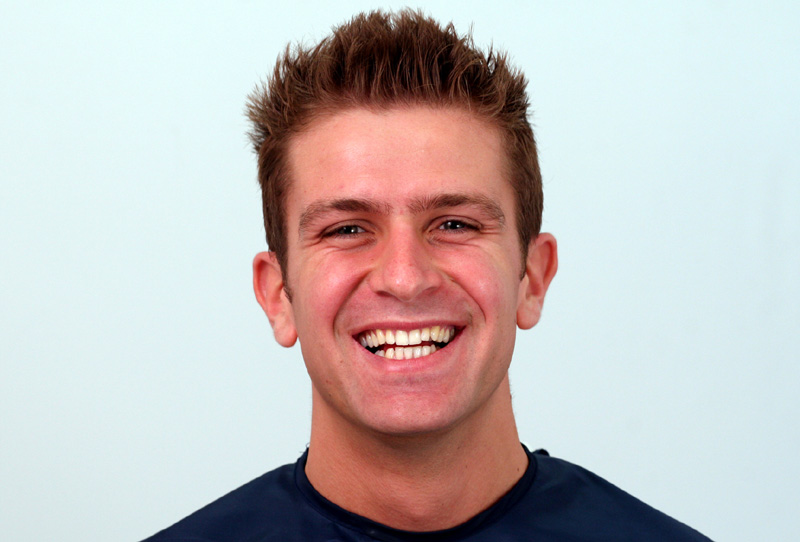

Supplement: Data Sheet 2 — WSEFEP - complete pictures dataset. [file DataSheet2.ZIP › KA_0043-lo.jpg]

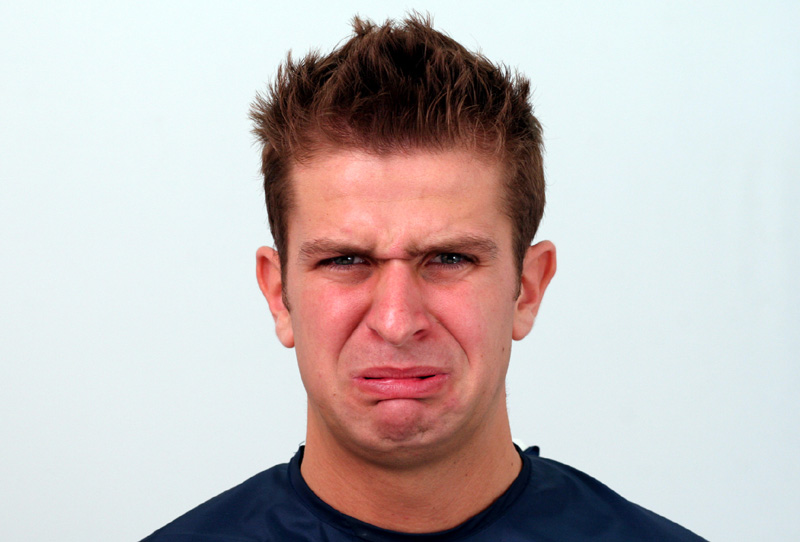

Supplement: Data Sheet 2 — WSEFEP - complete pictures dataset. [file DataSheet2.ZIP › KA_0535-lo.jpg]

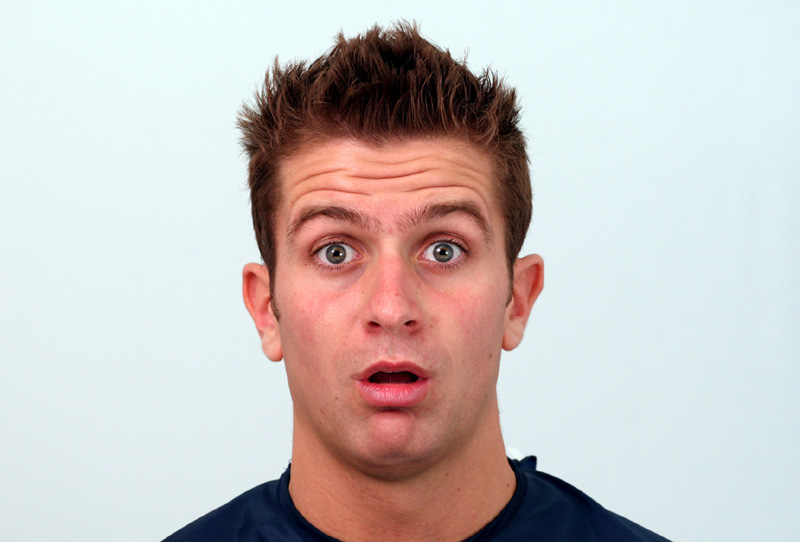

Supplement: Data Sheet 2 — WSEFEP - complete pictures dataset. [file DataSheet2.ZIP › KA_0884-lo.jpg]

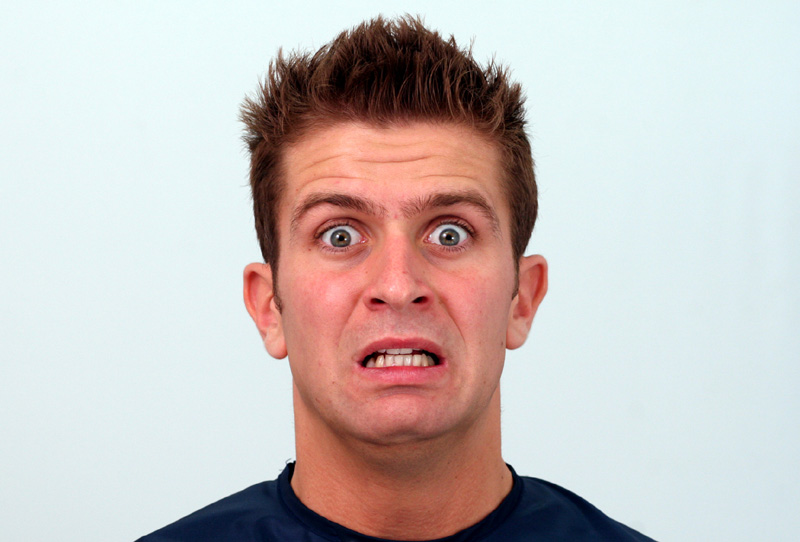

Supplement: Data Sheet 2 — WSEFEP - complete pictures dataset. [file DataSheet2.ZIP › KA_1134-lo.jpg]

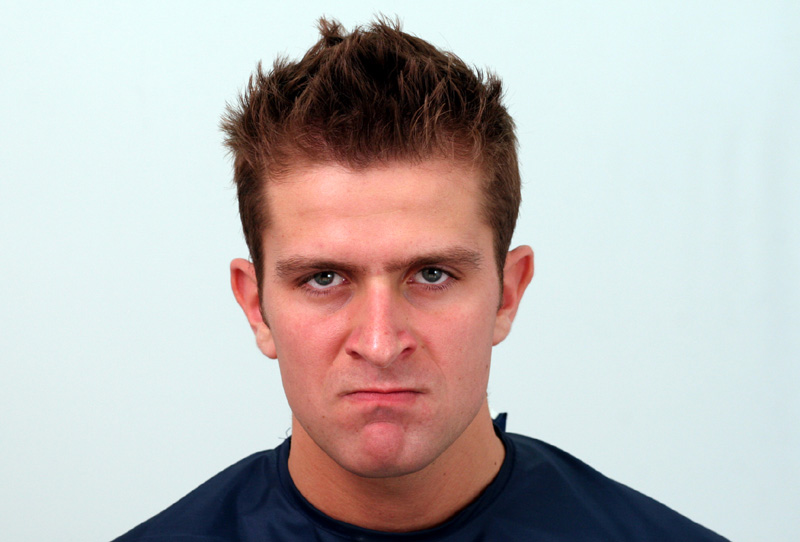

Supplement: Data Sheet 2 — WSEFEP - complete pictures dataset. [file DataSheet2.ZIP › KA_1616-lo.jpg]

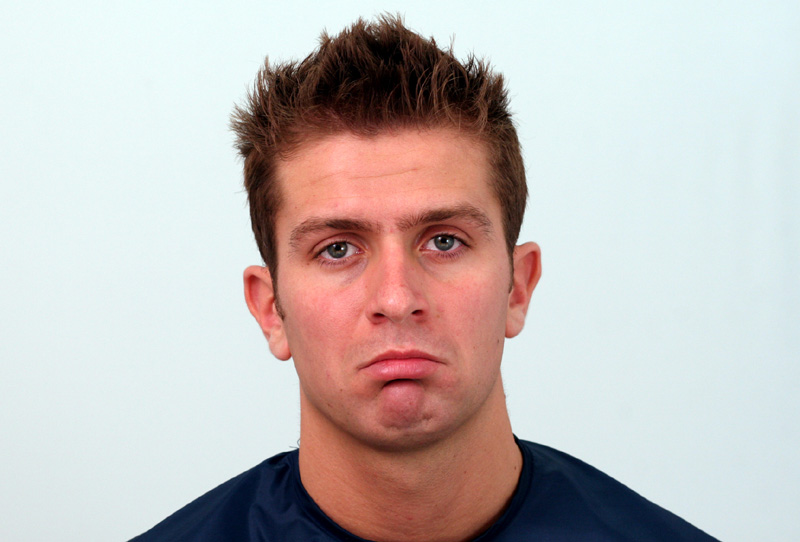

Supplement: Data Sheet 2 — WSEFEP - complete pictures dataset. [file DataSheet2.ZIP › KA_2396-lo.jpg]

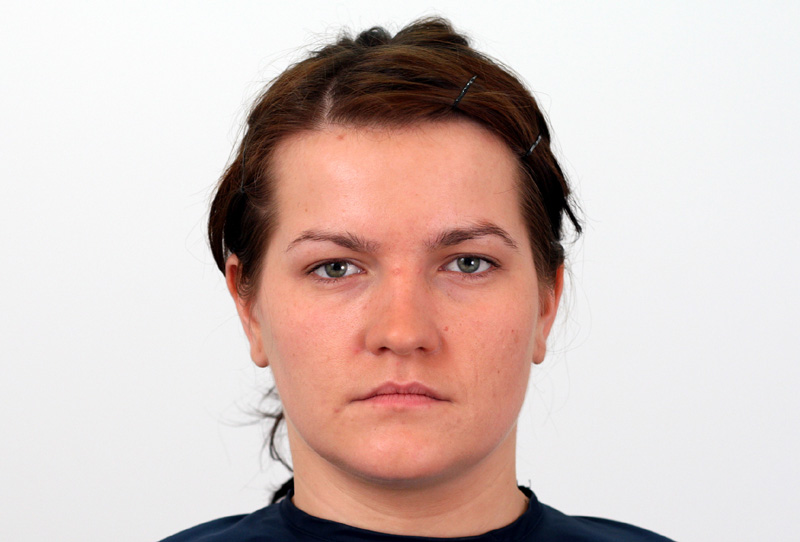

Supplement: Data Sheet 2 — WSEFEP - complete pictures dataset. [file DataSheet2.ZIP › KL_0024-lo.jpg]

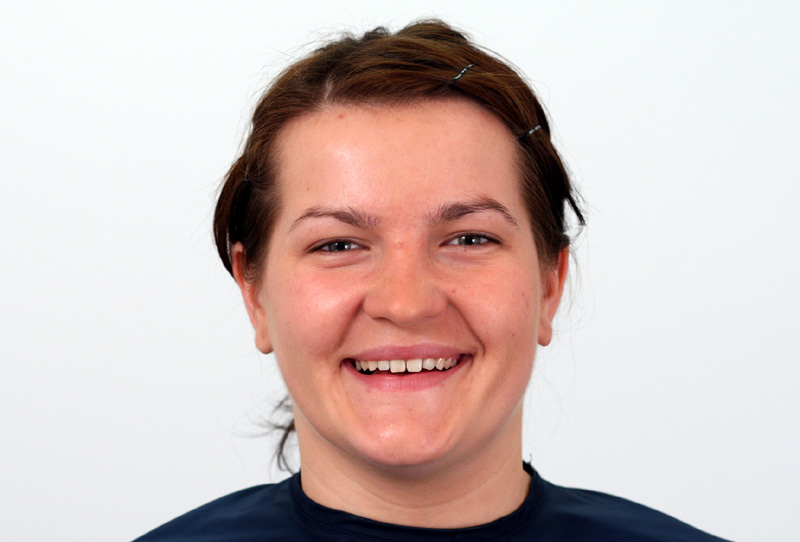

Supplement: Data Sheet 2 — WSEFEP - complete pictures dataset. [file DataSheet2.ZIP › KL_0092-lo.jpg]

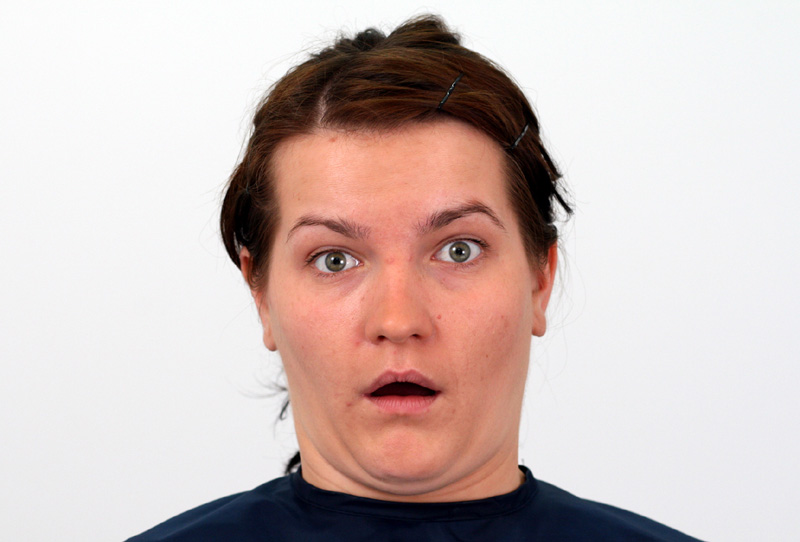

Supplement: Data Sheet 2 — WSEFEP - complete pictures dataset. [file DataSheet2.ZIP › KL_0324-lo.jpg]

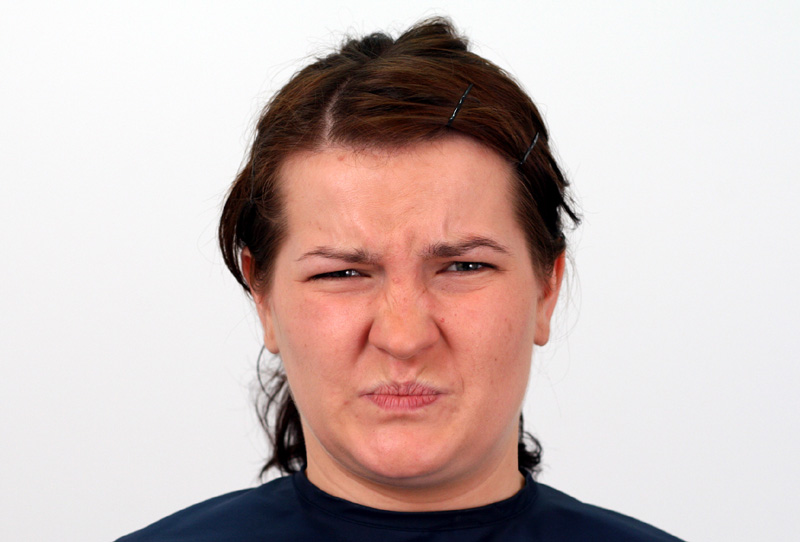

Supplement: Data Sheet 2 — WSEFEP - complete pictures dataset. [file DataSheet2.ZIP › KL_0697-lo.jpg]

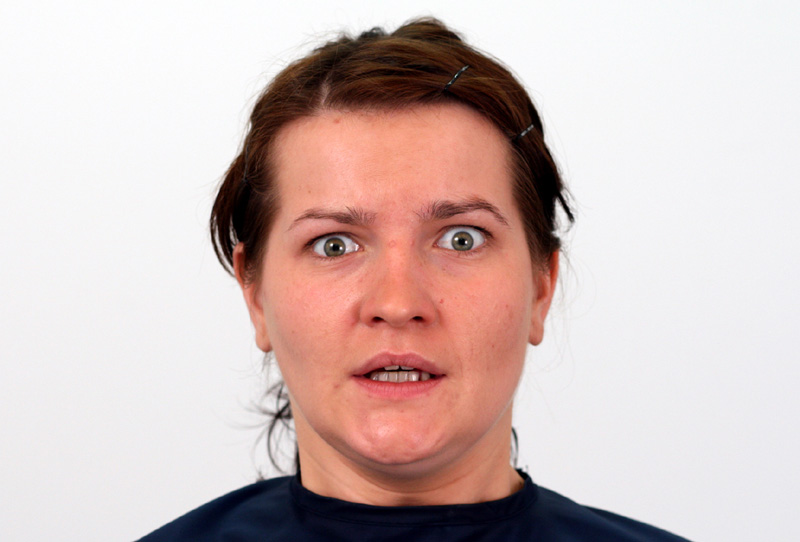

Supplement: Data Sheet 2 — WSEFEP - complete pictures dataset. [file DataSheet2.ZIP › KL_0900-lo.jpg]

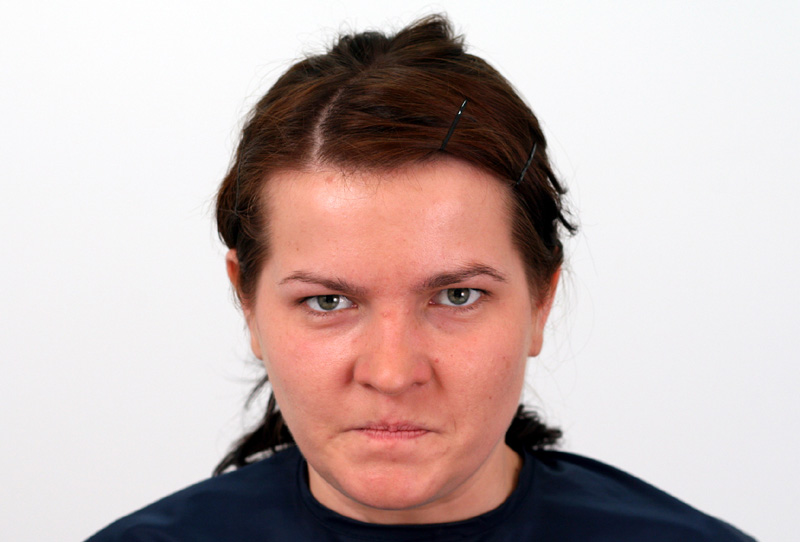

Supplement: Data Sheet 2 — WSEFEP - complete pictures dataset. [file DataSheet2.ZIP › KL_1182-lo.jpg]

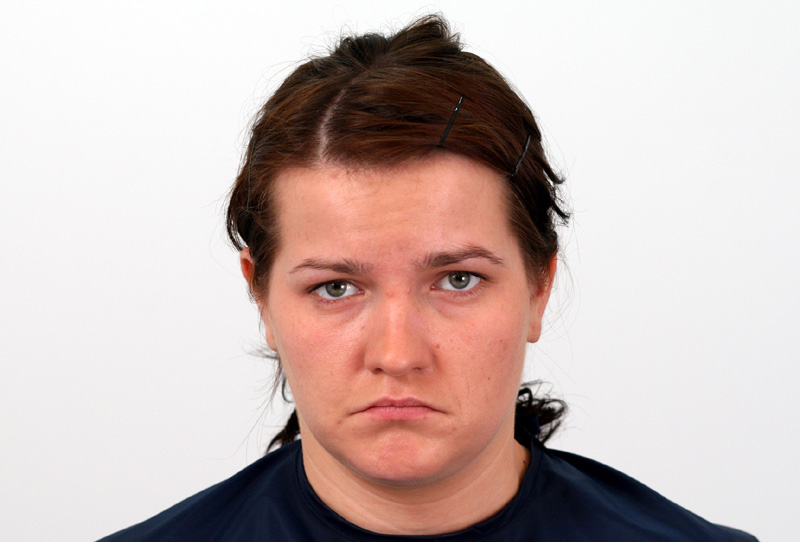

Supplement: Data Sheet 2 — WSEFEP - complete pictures dataset. [file DataSheet2.ZIP › KL_1438-lo.jpg]

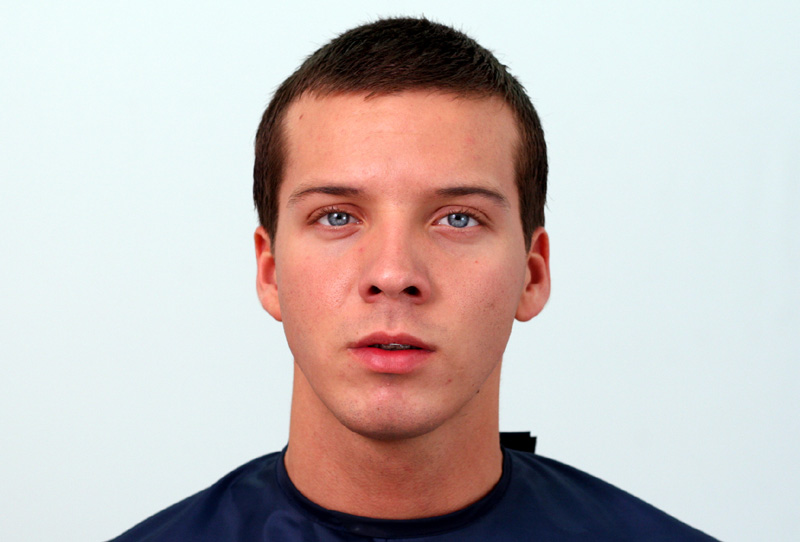

Supplement: Data Sheet 2 — WSEFEP - complete pictures dataset. [file DataSheet2.ZIP › KM_0017-lo.jpg]

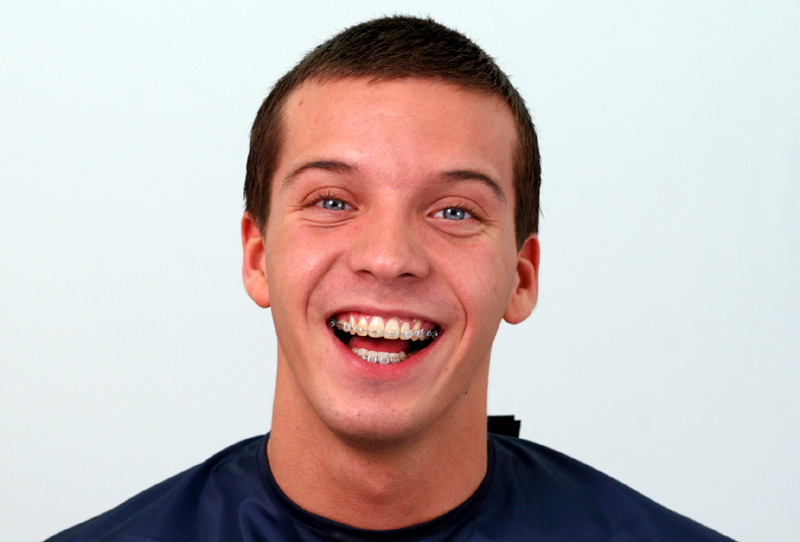

Supplement: Data Sheet 2 — WSEFEP - complete pictures dataset. [file DataSheet2.ZIP › KM_0137-lo.jpg]

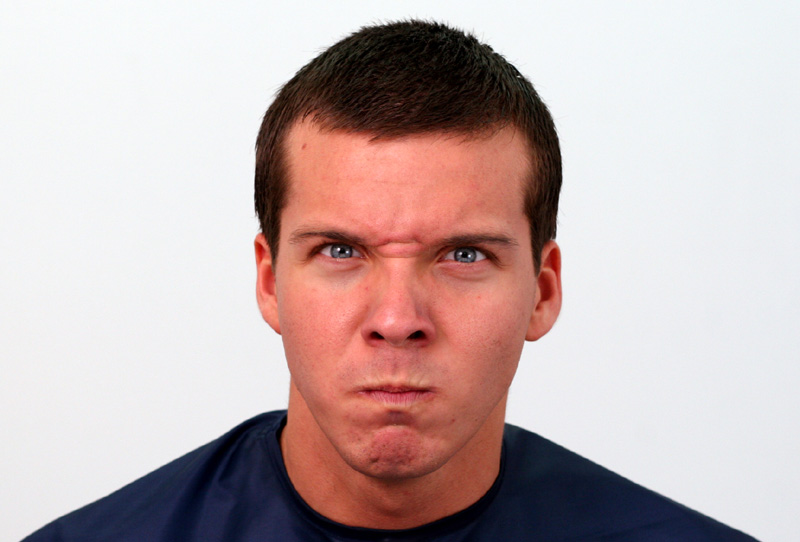

Supplement: Data Sheet 2 — WSEFEP - complete pictures dataset. [file DataSheet2.ZIP › KM_0620-lo.jpg]

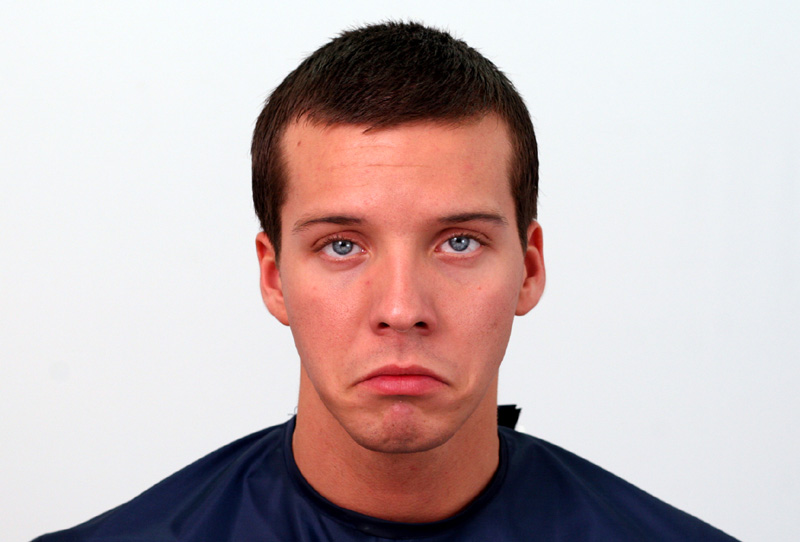

Supplement: Data Sheet 2 — WSEFEP - complete pictures dataset. [file DataSheet2.ZIP › KM_1295-lo.jpg]

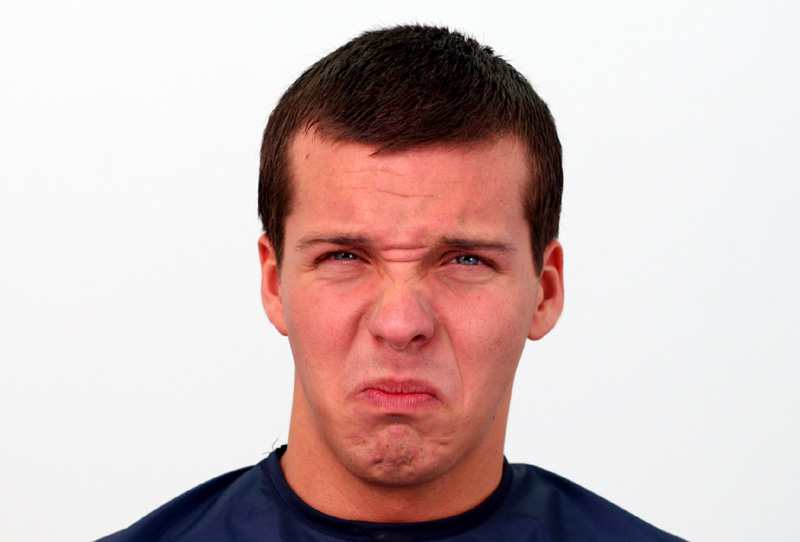

Supplement: Data Sheet 2 — WSEFEP - complete pictures dataset. [file DataSheet2.ZIP › KM_1583-lo.jpg]

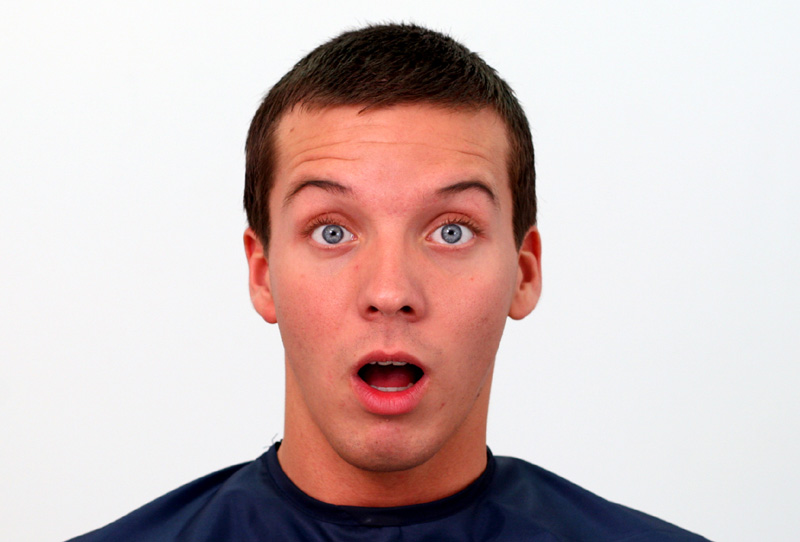

Supplement: Data Sheet 2 — WSEFEP - complete pictures dataset. [file DataSheet2.ZIP › KM_1797-lo.jpg]

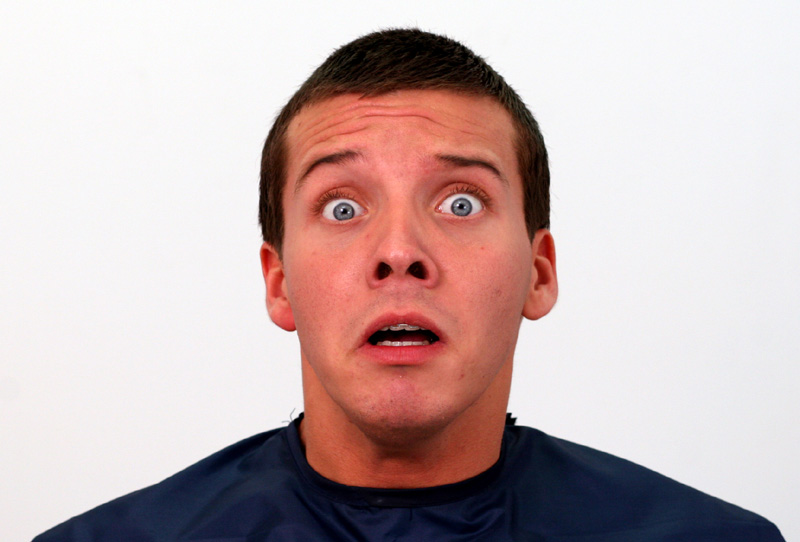

Supplement: Data Sheet 2 — WSEFEP - complete pictures dataset. [file DataSheet2.ZIP › KM_1980-lo.jpg]

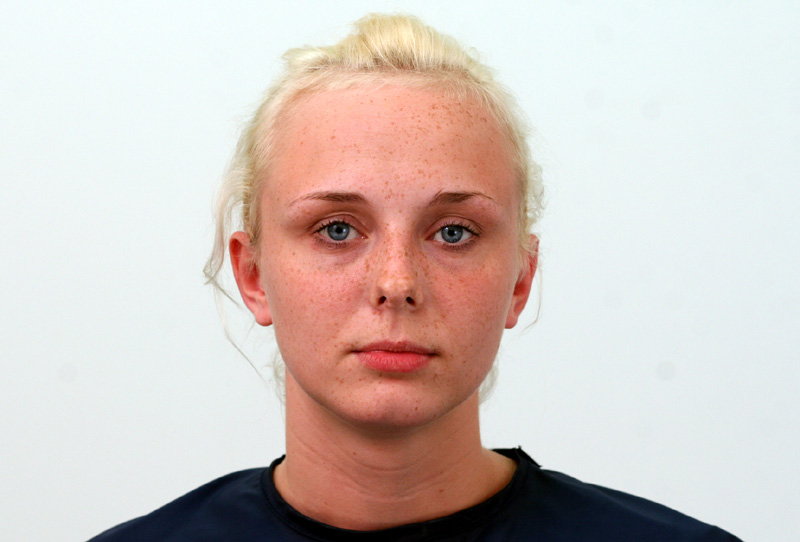

Supplement: Data Sheet 2 — WSEFEP - complete pictures dataset. [file DataSheet2.ZIP › KO_0031-lo.jpg]

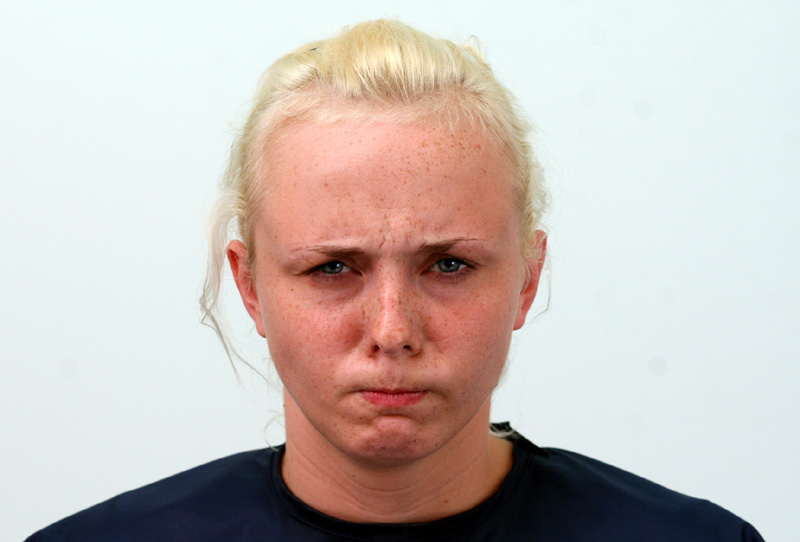

Supplement: Data Sheet 2 — WSEFEP - complete pictures dataset. [file DataSheet2.ZIP › KO_0251-lo.jpg]

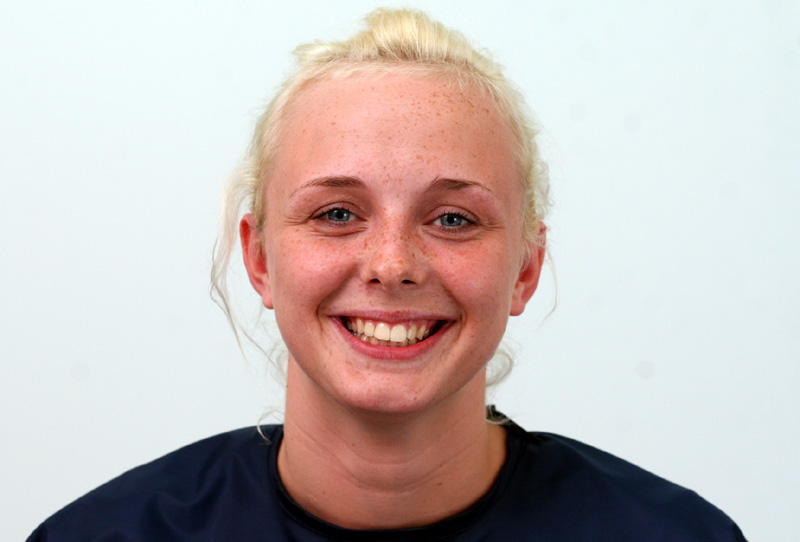

Supplement: Data Sheet 2 — WSEFEP - complete pictures dataset. [file DataSheet2.ZIP › KO_0277-lo.jpg]

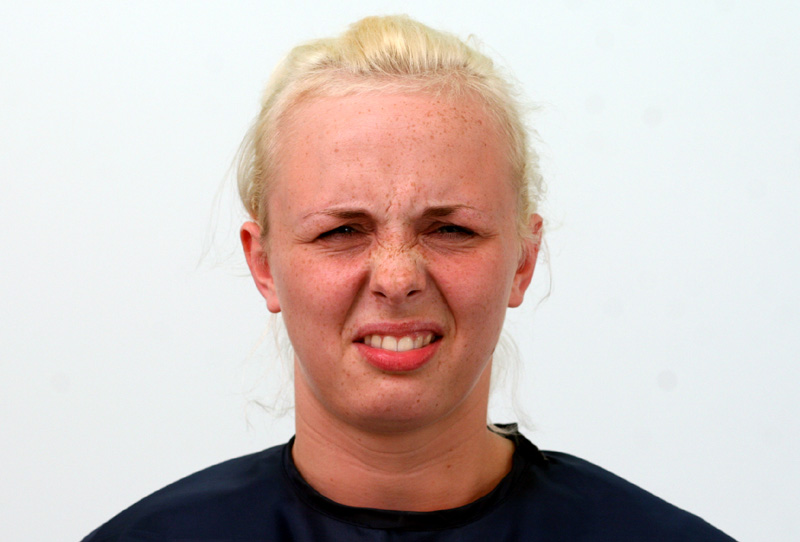

Supplement: Data Sheet 2 — WSEFEP - complete pictures dataset. [file DataSheet2.ZIP › KO_0484-lo.jpg]

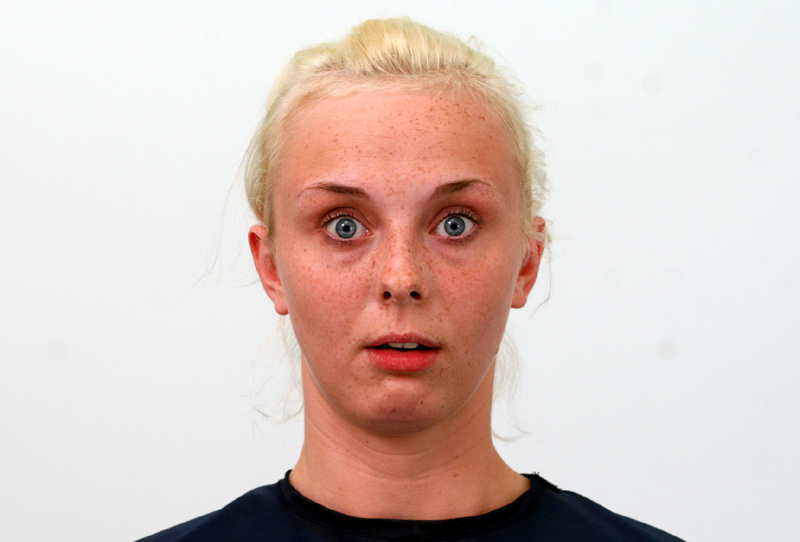

Supplement: Data Sheet 2 — WSEFEP - complete pictures dataset. [file DataSheet2.ZIP › KO_0624-lo.jpg]

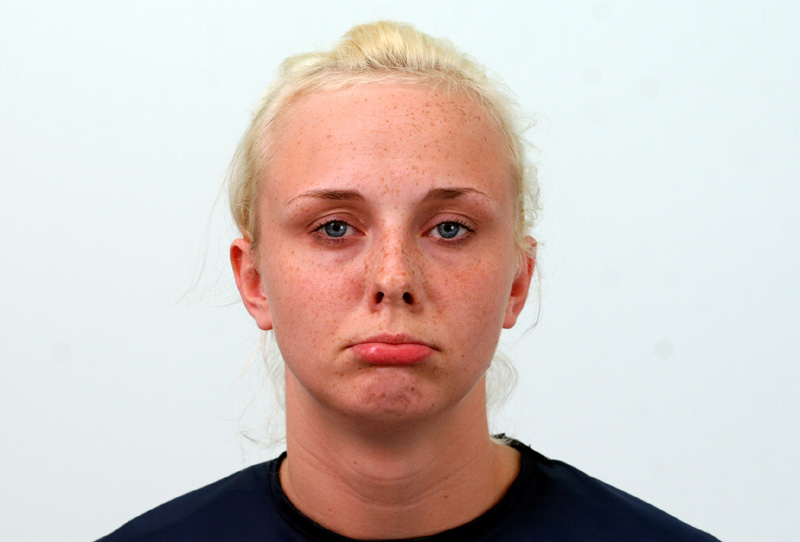

Supplement: Data Sheet 2 — WSEFEP - complete pictures dataset. [file DataSheet2.ZIP › KO_0665-lo.jpg]

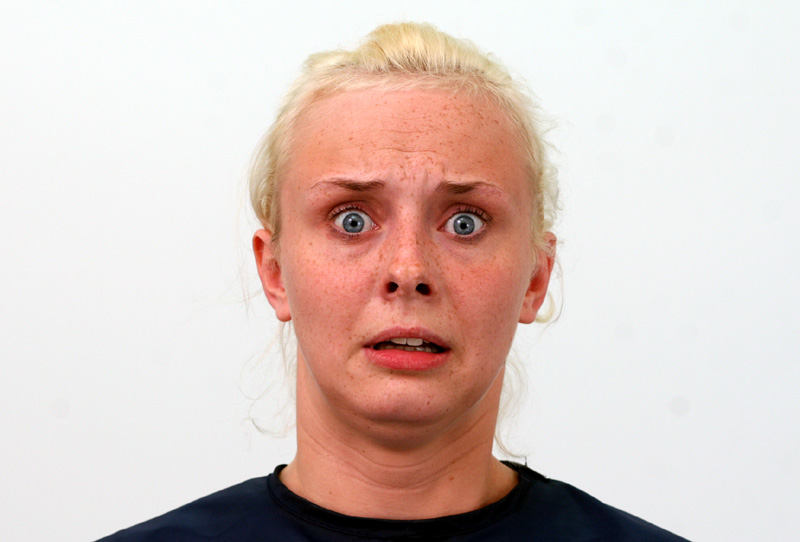

Supplement: Data Sheet 2 — WSEFEP - complete pictures dataset. [file DataSheet2.ZIP › KO_1082-lo.jpg]

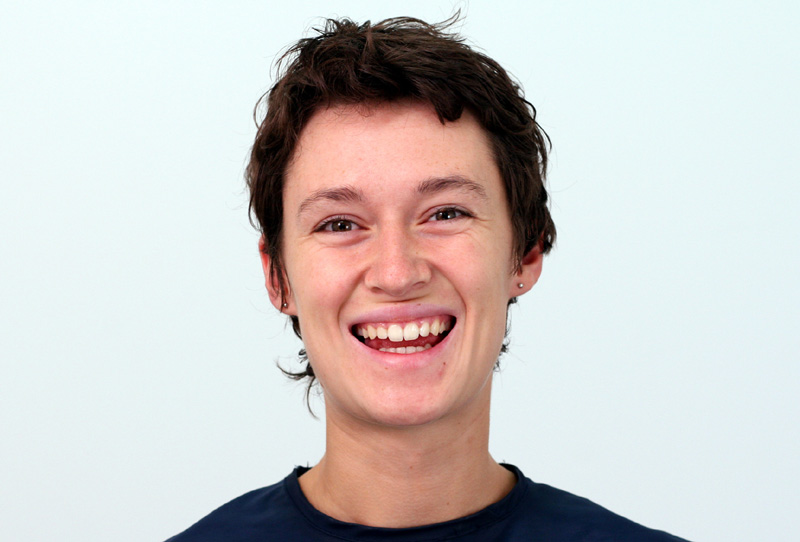

Supplement: Data Sheet 2 — WSEFEP - complete pictures dataset. [file DataSheet2.ZIP › KP_0051-lo.jpg]

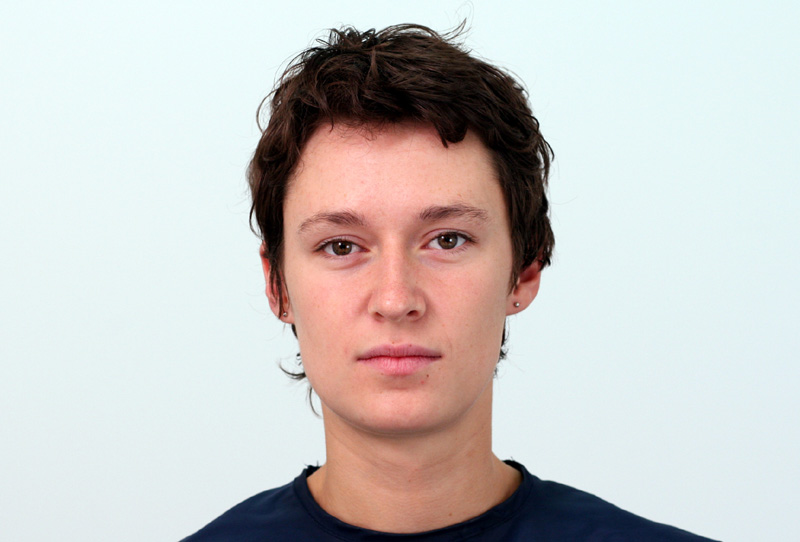

Supplement: Data Sheet 2 — WSEFEP - complete pictures dataset. [file DataSheet2.ZIP › KP_0082-lo.jpg]

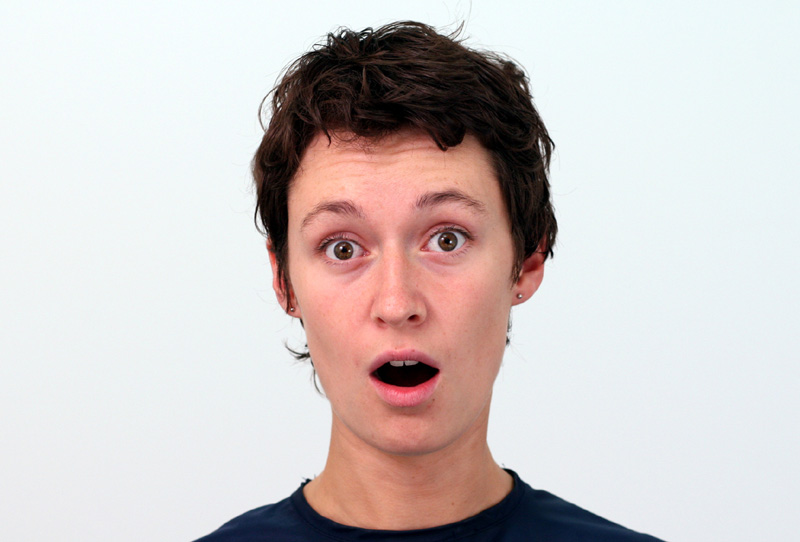

Supplement: Data Sheet 2 — WSEFEP - complete pictures dataset. [file DataSheet2.ZIP › KP_0225-lo.jpg]

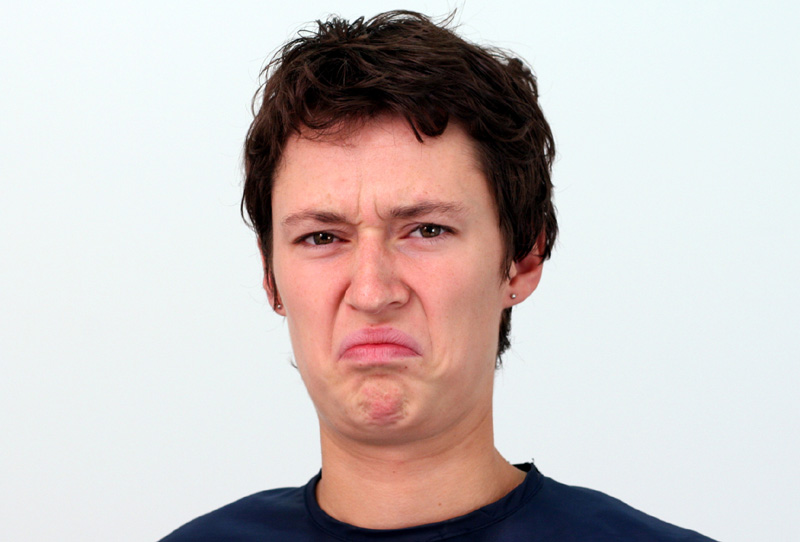

Supplement: Data Sheet 2 — WSEFEP - complete pictures dataset. [file DataSheet2.ZIP › KP_0351-lo.jpg]

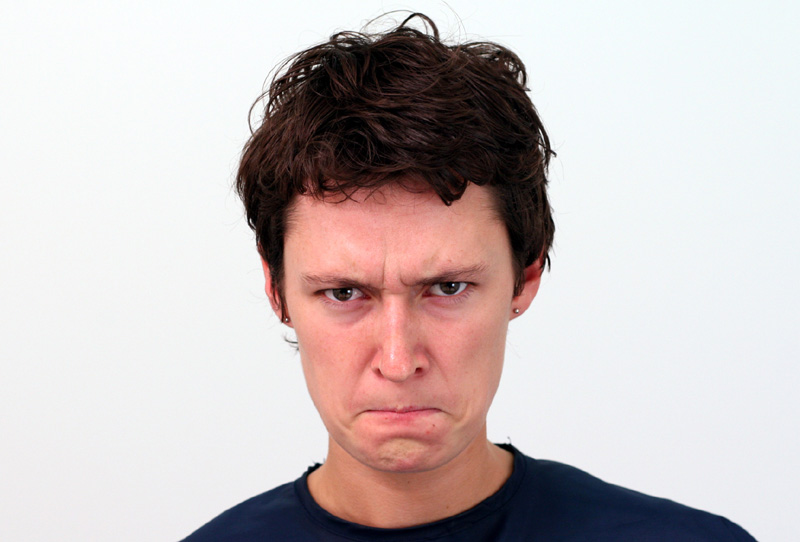

Supplement: Data Sheet 2 — WSEFEP - complete pictures dataset. [file DataSheet2.ZIP › KP_0760-lo.jpg]

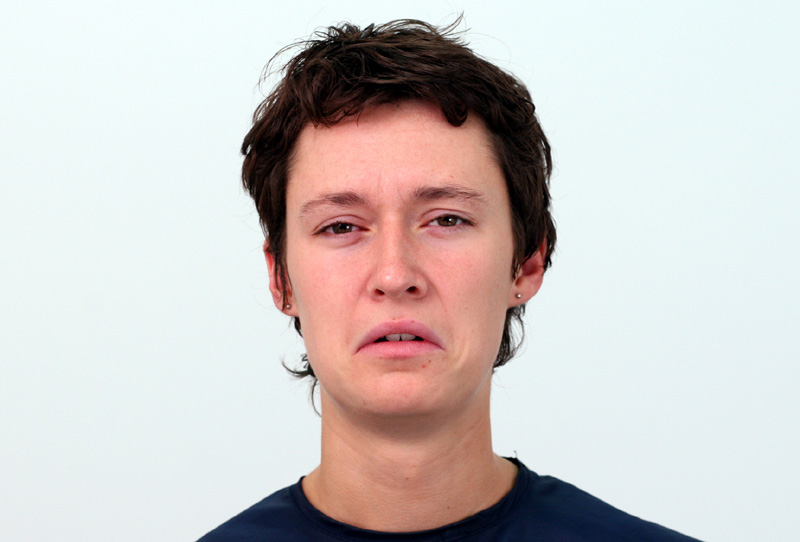

Supplement: Data Sheet 2 — WSEFEP - complete pictures dataset. [file DataSheet2.ZIP › KP_0991-lo.jpg]

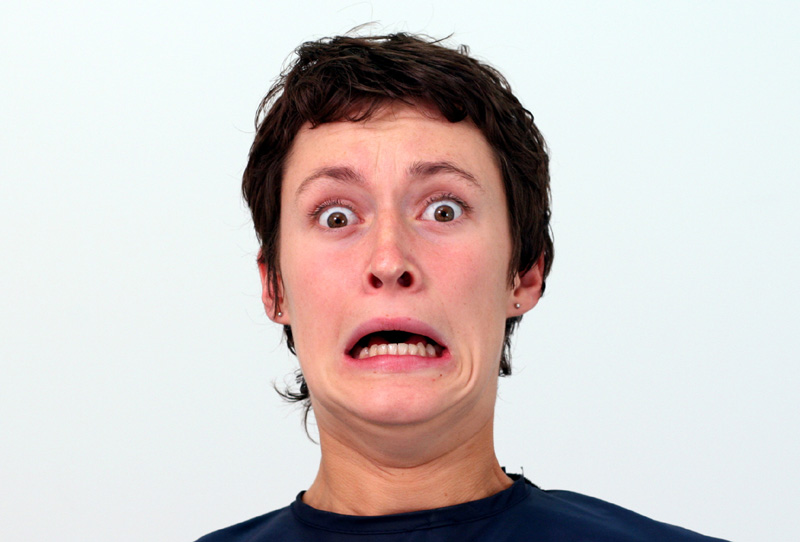

Supplement: Data Sheet 2 — WSEFEP - complete pictures dataset. [file DataSheet2.ZIP › KP_1148-lo.jpg]

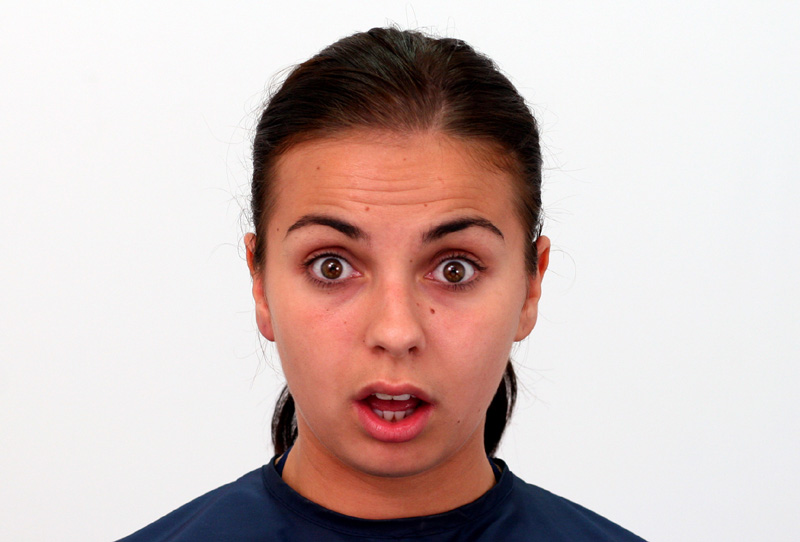

Supplement: Data Sheet 2 — WSEFEP - complete pictures dataset. [file DataSheet2.ZIP › KS_0252-lo.jpg]

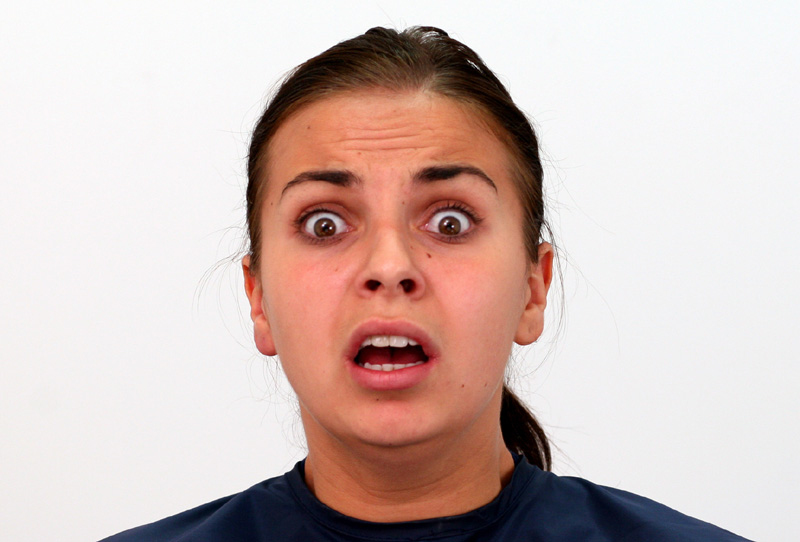

Supplement: Data Sheet 2 — WSEFEP - complete pictures dataset. [file DataSheet2.ZIP › KS_0624-lo.jpg]

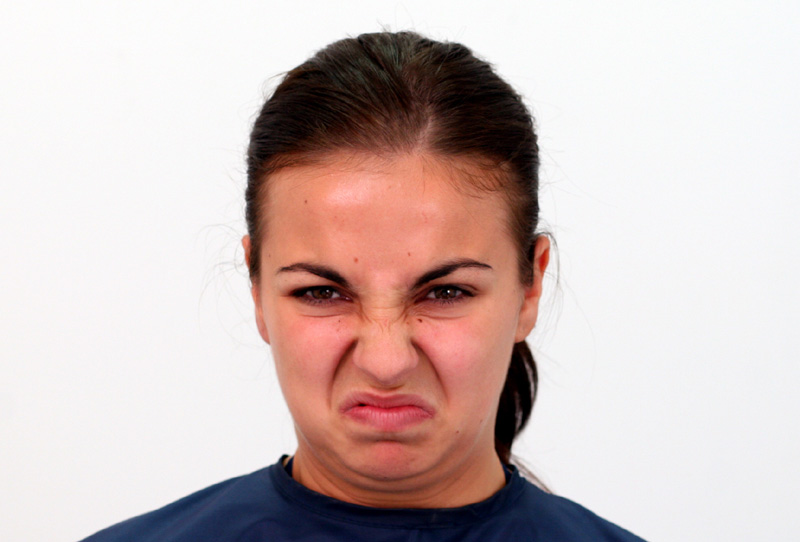

Supplement: Data Sheet 2 — WSEFEP - complete pictures dataset. [file DataSheet2.ZIP › KS_0993-lo.jpg]

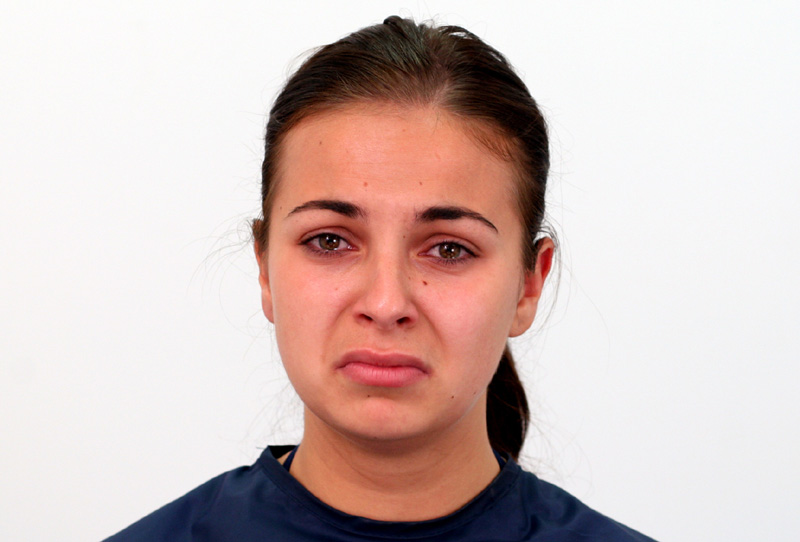

Supplement: Data Sheet 2 — WSEFEP - complete pictures dataset. [file DataSheet2.ZIP › KS_2052-lo.jpg]

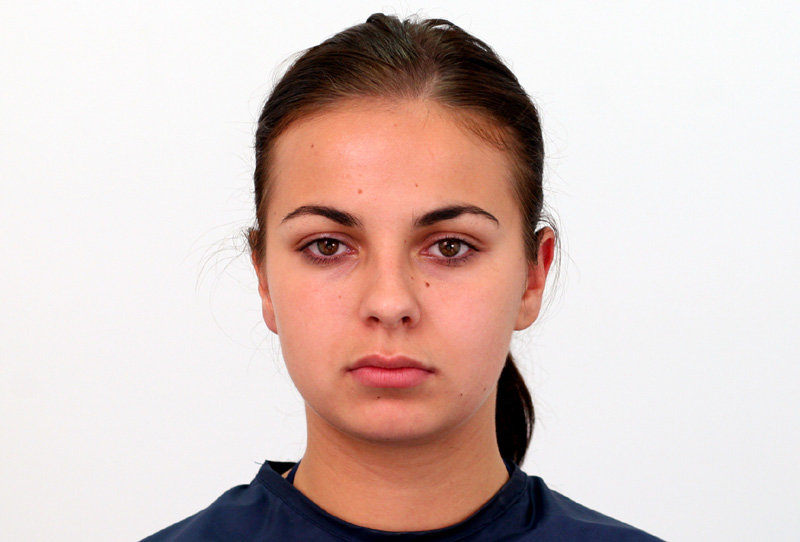

Supplement: Data Sheet 2 — WSEFEP - complete pictures dataset. [file DataSheet2.ZIP › KS_2161-lo.jpg]

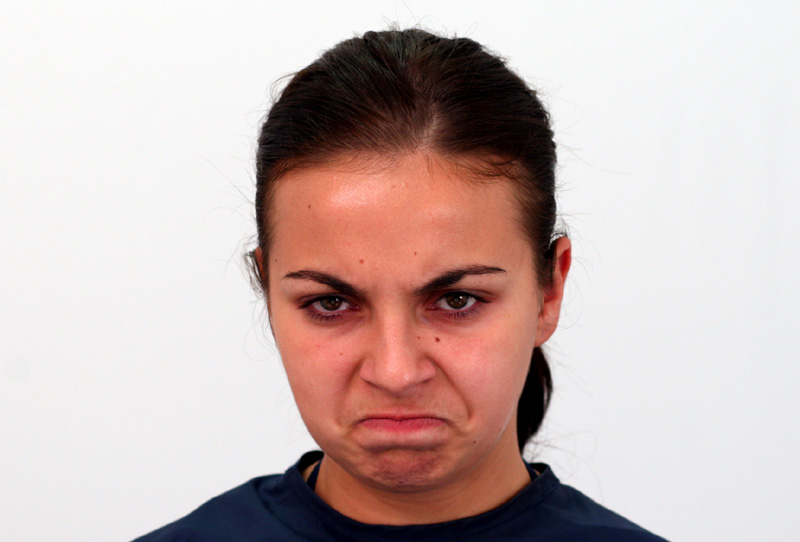

Supplement: Data Sheet 2 — WSEFEP - complete pictures dataset. [file DataSheet2.ZIP › KS_3640-lo.jpg]

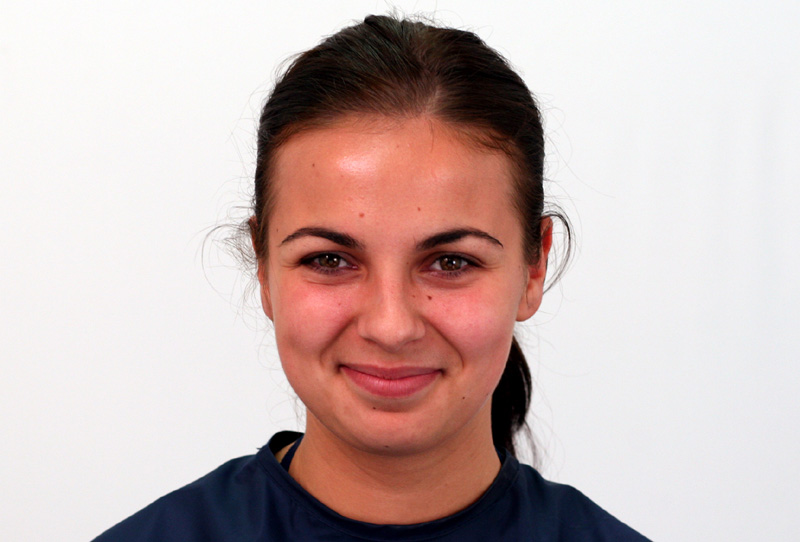

Supplement: Data Sheet 2 — WSEFEP - complete pictures dataset. [file DataSheet2.ZIP › KS_4222-lo.jpg]

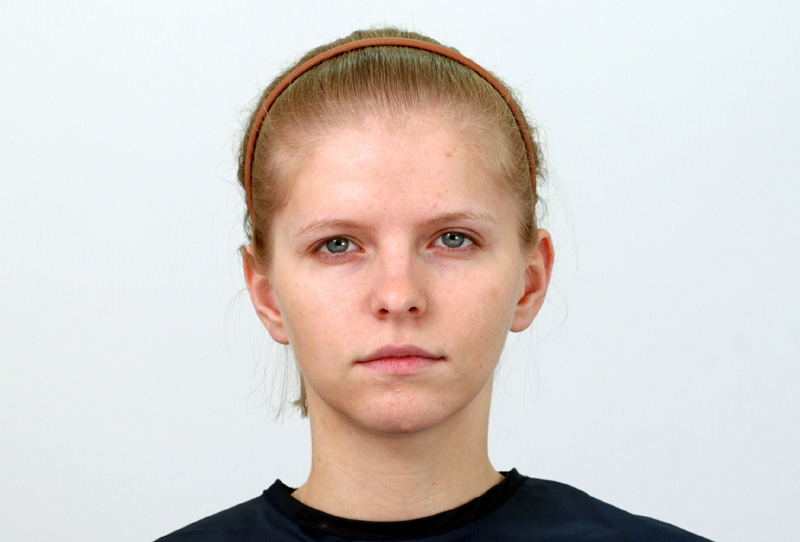

Supplement: Data Sheet 2 — WSEFEP - complete pictures dataset. [file DataSheet2.ZIP › MB_0026-lo.jpg]

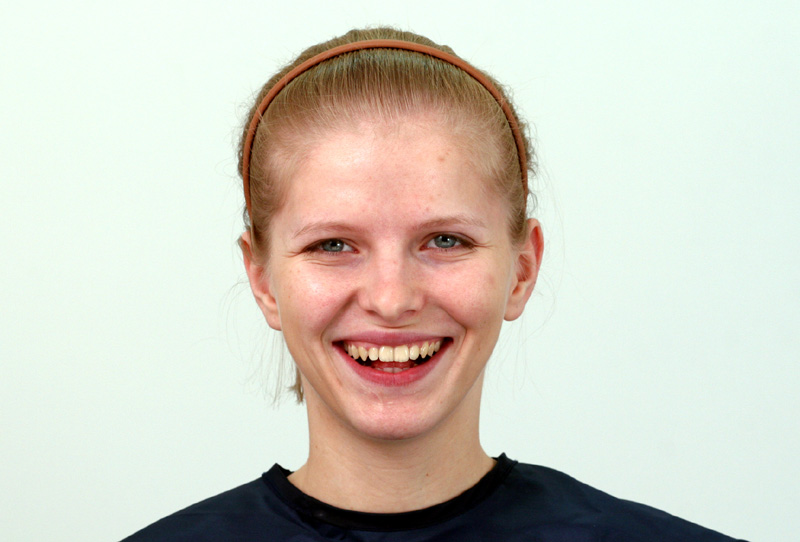

Supplement: Data Sheet 2 — WSEFEP - complete pictures dataset. [file DataSheet2.ZIP › MB_0048-lo.jpg]

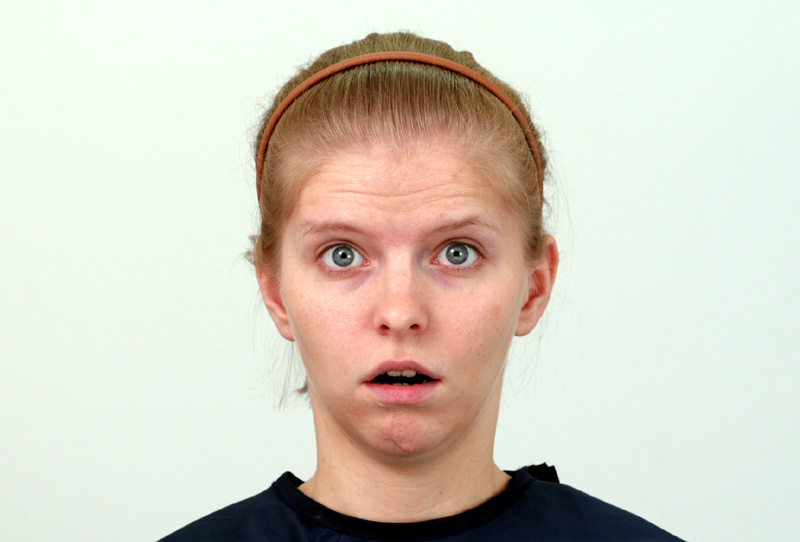

Supplement: Data Sheet 2 — WSEFEP - complete pictures dataset. [file DataSheet2.ZIP › MB_0400-lo.jpg]

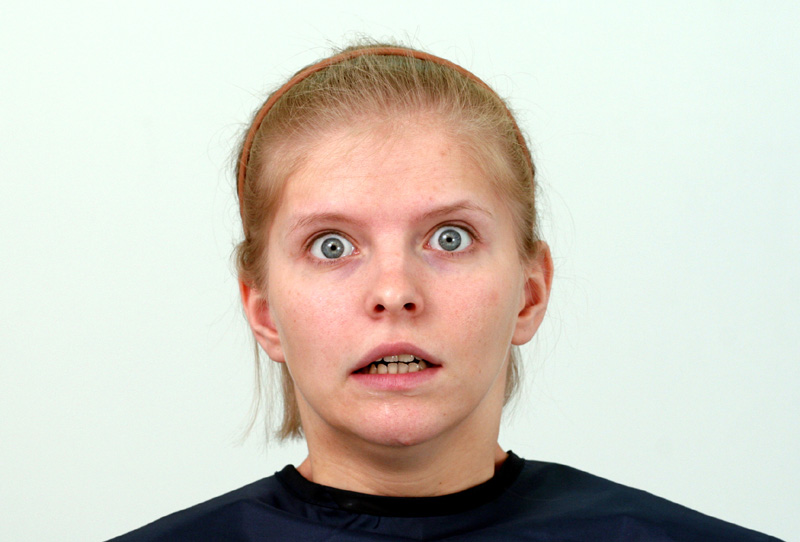

Supplement: Data Sheet 2 — WSEFEP - complete pictures dataset. [file DataSheet2.ZIP › MB_1031-lo.jpg]

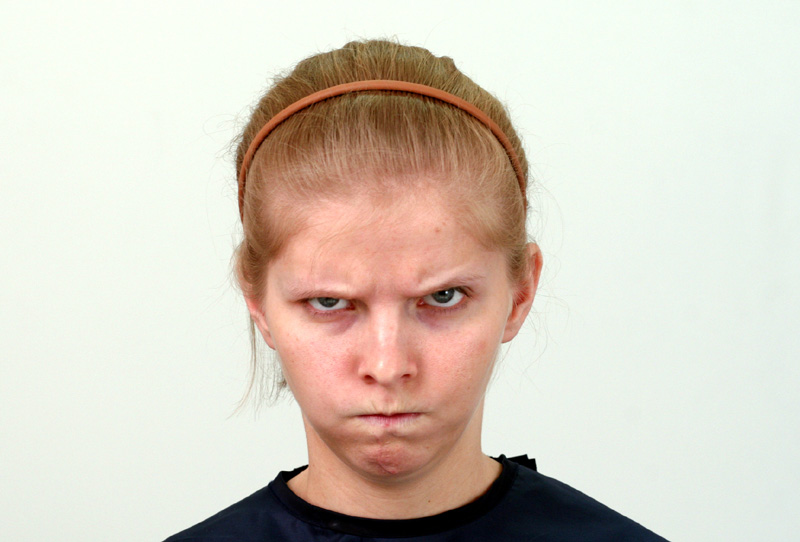

Supplement: Data Sheet 2 — WSEFEP - complete pictures dataset. [file DataSheet2.ZIP › MB_1210-lo.jpg]

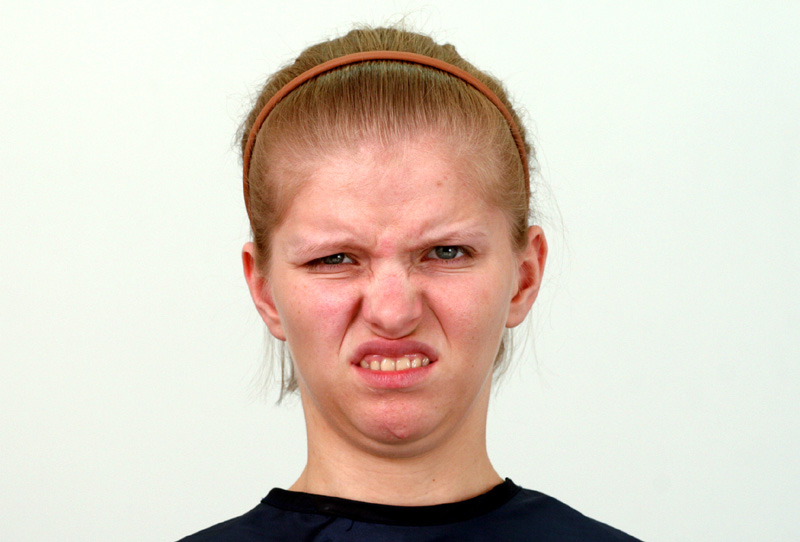

Supplement: Data Sheet 2 — WSEFEP - complete pictures dataset. [file DataSheet2.ZIP › MB_2133-lo.jpg]

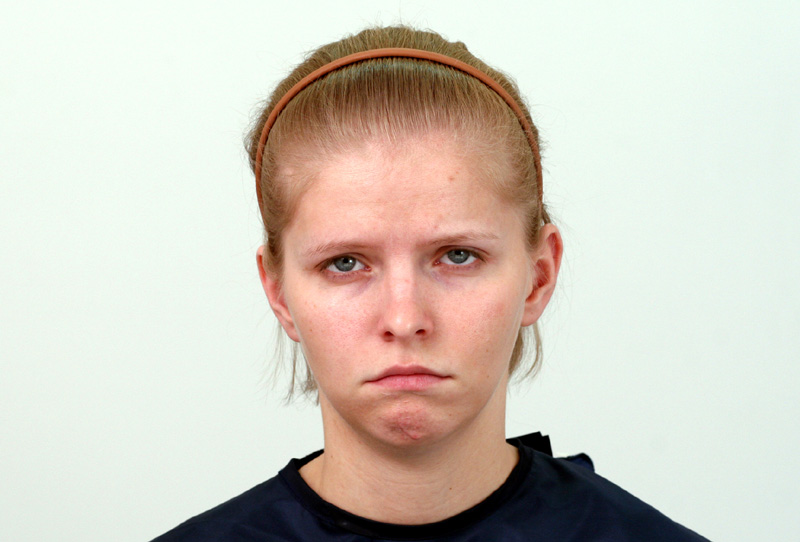

Supplement: Data Sheet 2 — WSEFEP - complete pictures dataset. [file DataSheet2.ZIP › MB_2362-lo.jpg]

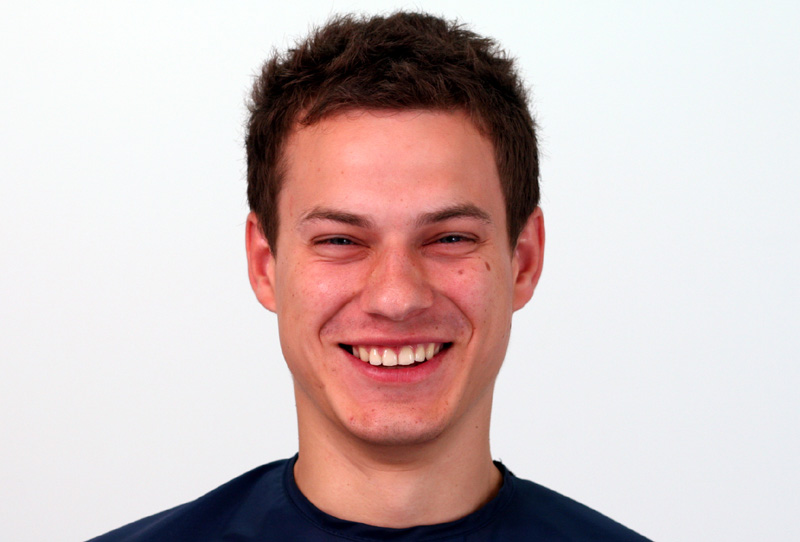

Supplement: Data Sheet 2 — WSEFEP - complete pictures dataset. [file DataSheet2.ZIP › MG_0330-lo.jpg]

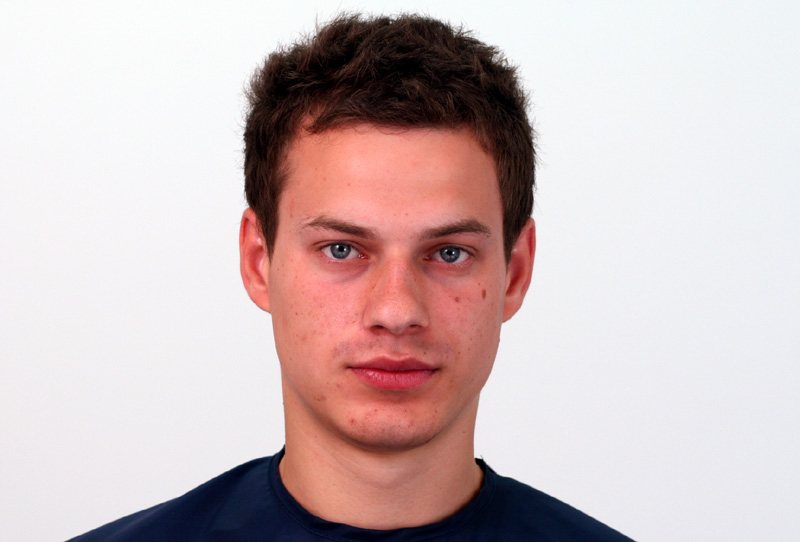

Supplement: Data Sheet 2 — WSEFEP - complete pictures dataset. [file DataSheet2.ZIP › MG_0345-lo.jpg]

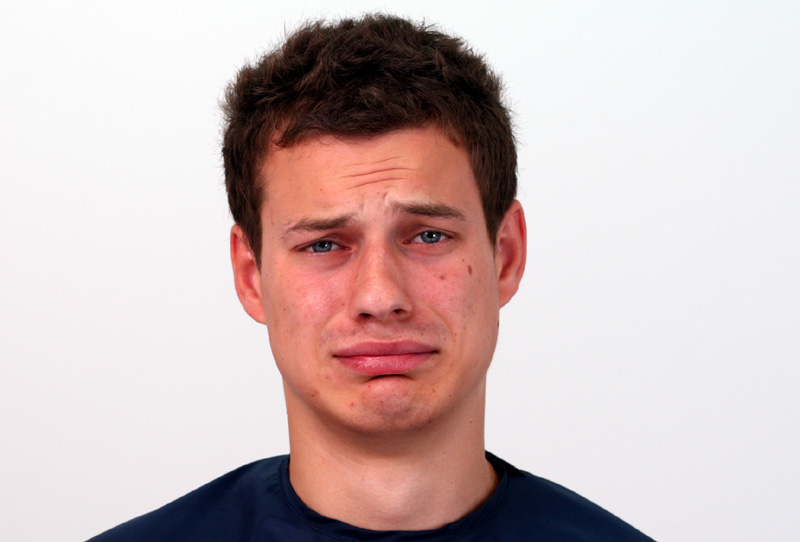

Supplement: Data Sheet 2 — WSEFEP - complete pictures dataset. [file DataSheet2.ZIP › MG_0754-lo.jpg]

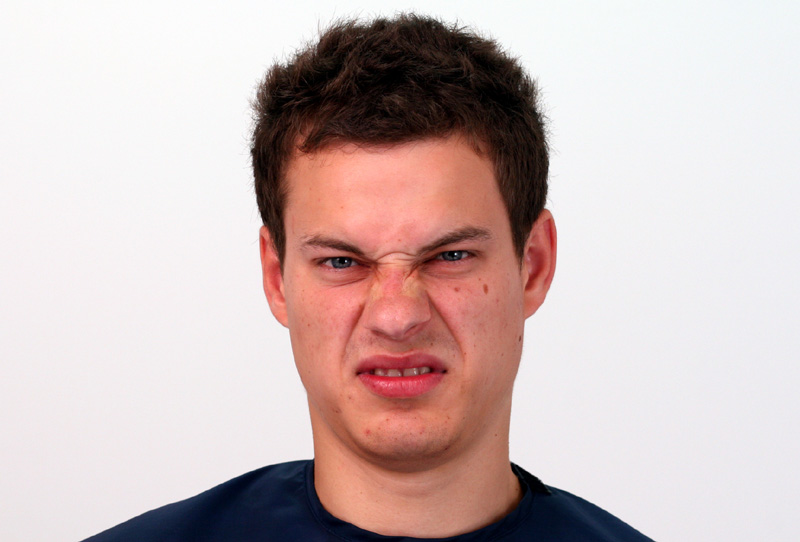

Supplement: Data Sheet 2 — WSEFEP - complete pictures dataset. [file DataSheet2.ZIP › MG_0928-lo.jpg]

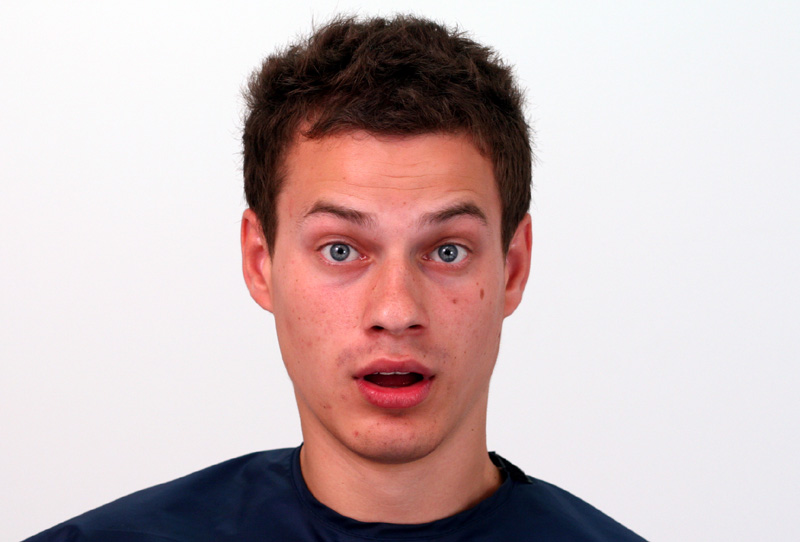

Supplement: Data Sheet 2 — WSEFEP - complete pictures dataset. [file DataSheet2.ZIP › MG_1069-lo.jpg]

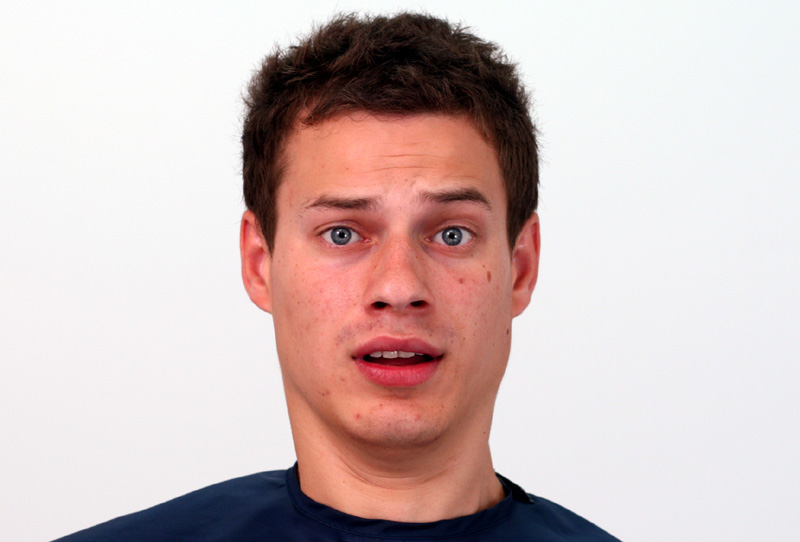

Supplement: Data Sheet 2 — WSEFEP - complete pictures dataset. [file DataSheet2.ZIP › MG_1280-lo.jpg]

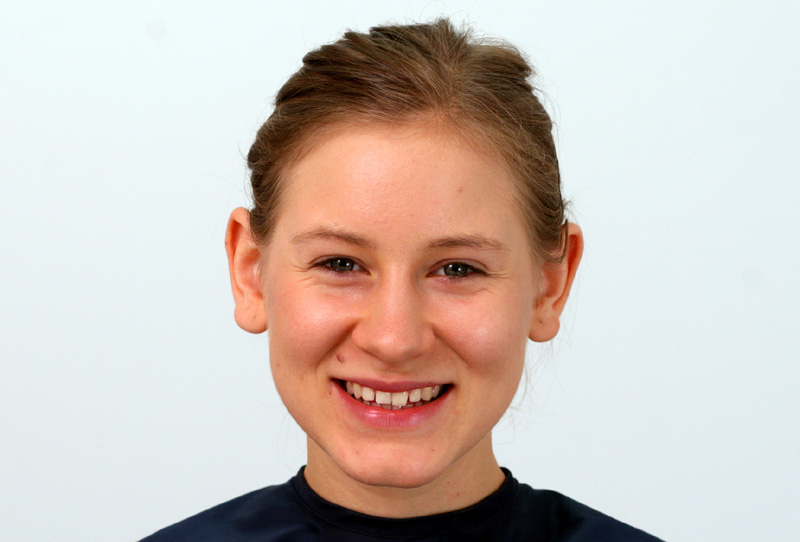

Supplement: Data Sheet 2 — WSEFEP - complete pictures dataset. [file DataSheet2.ZIP › MJ_0066-lo.jpg]

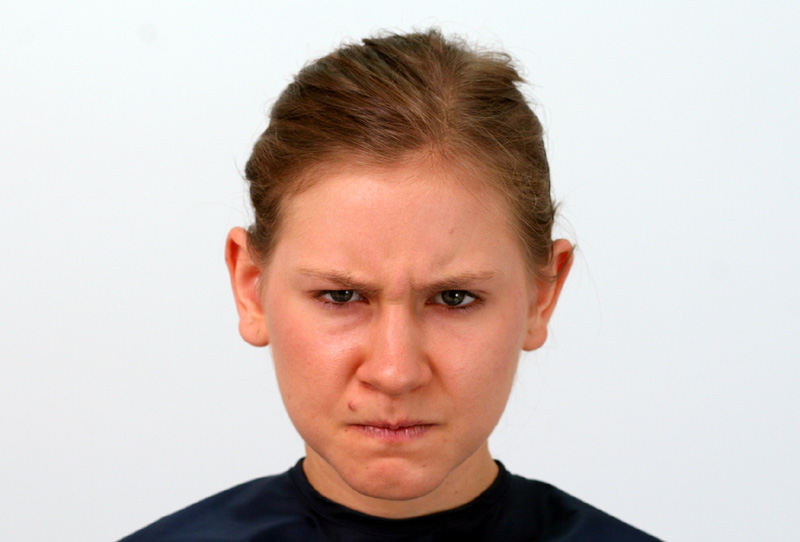

Supplement: Data Sheet 2 — WSEFEP - complete pictures dataset. [file DataSheet2.ZIP › MJ_0152-lo.jpg]

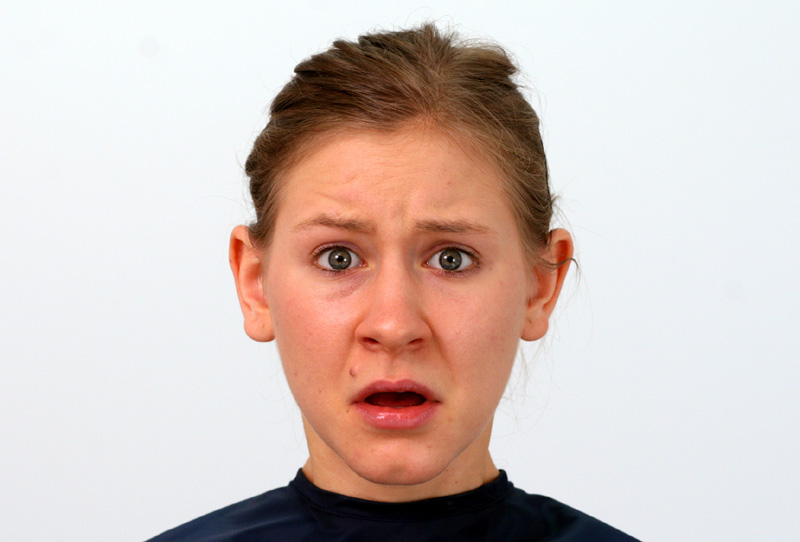

Supplement: Data Sheet 2 — WSEFEP - complete pictures dataset. [file DataSheet2.ZIP › MJ_0271-lo.jpg]

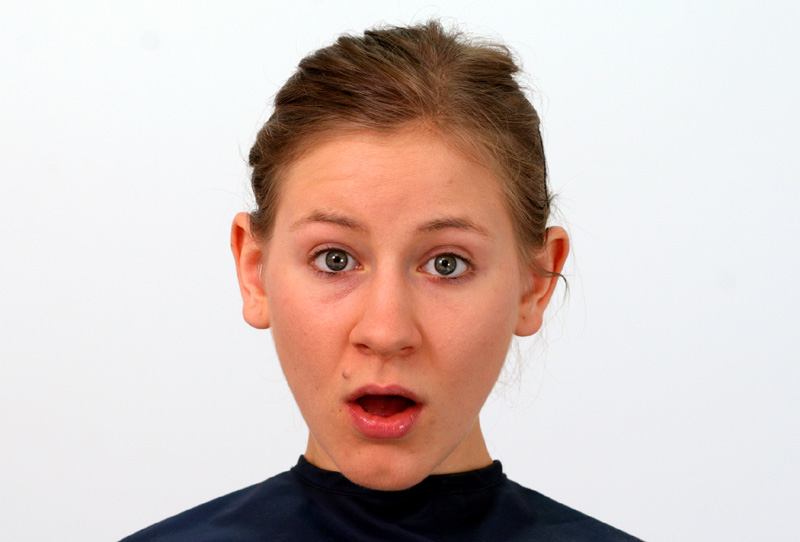

Supplement: Data Sheet 2 — WSEFEP - complete pictures dataset. [file DataSheet2.ZIP › MJ_0332-lo.jpg]

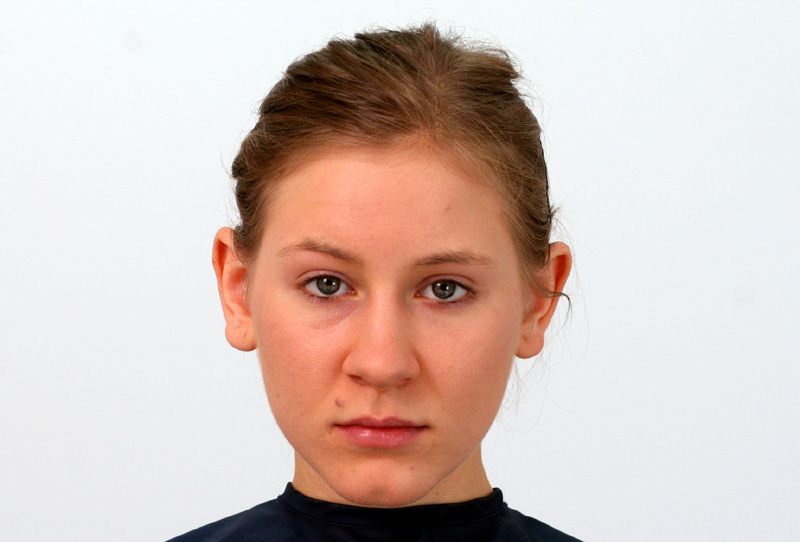

Supplement: Data Sheet 2 — WSEFEP - complete pictures dataset. [file DataSheet2.ZIP › MJ_0346-lo.jpg]

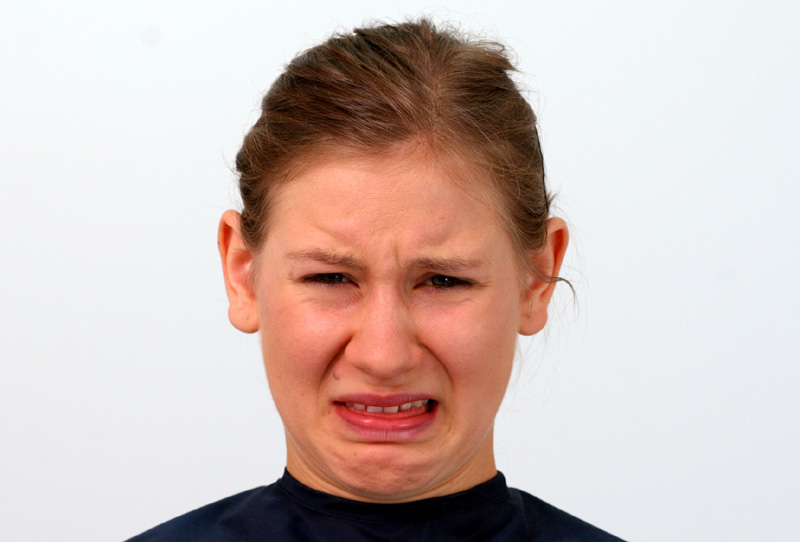

Supplement: Data Sheet 2 — WSEFEP - complete pictures dataset. [file DataSheet2.ZIP › MJ_0370-lo.jpg]

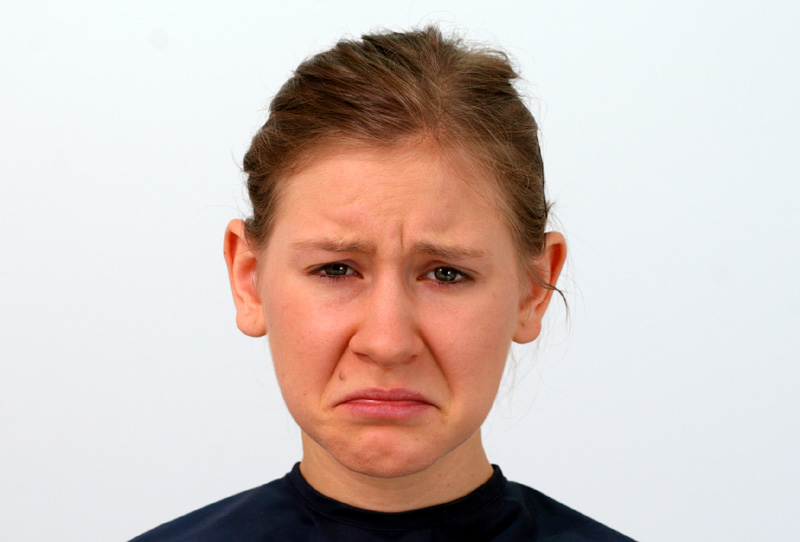

Supplement: Data Sheet 2 — WSEFEP - complete pictures dataset. [file DataSheet2.ZIP › MJ_0484-lo.jpg]

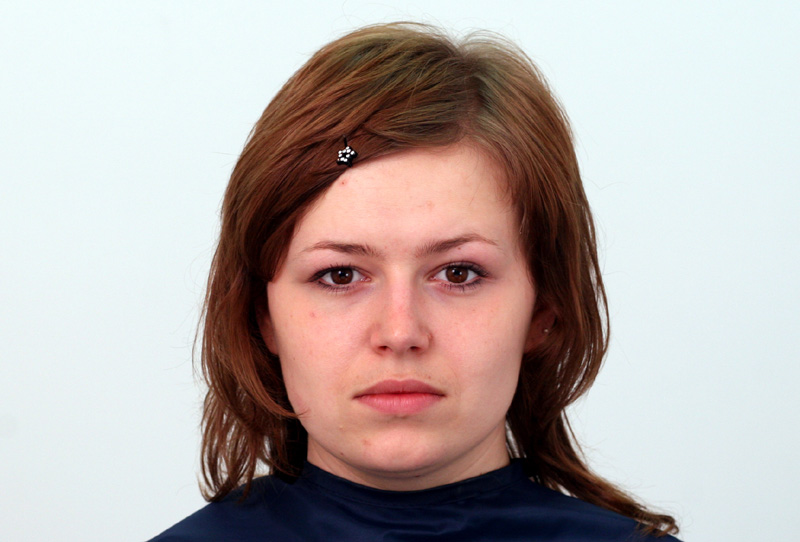

Supplement: Data Sheet 2 — WSEFEP - complete pictures dataset. [file DataSheet2.ZIP › MK1_0007-lo.jpg]

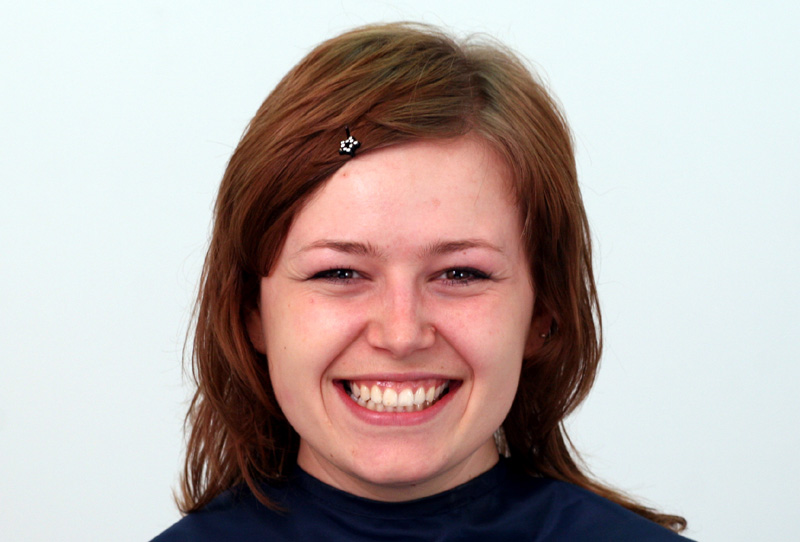

Supplement: Data Sheet 2 — WSEFEP - complete pictures dataset. [file DataSheet2.ZIP › MK1_0087-lo.jpg]
